# Widespread changes in sexual behaviour in eastern and southern Africa: Challenges to achieving global HIV targets? Longitudinal analyses of nationally representative surveys

**DOI:** 10.1002/jia2.25329

**Published:** 2019-06-21

**Authors:** Robin Schaefer, Simon Gregson, Clemens Benedikt

**Affiliations:** ^1^ MRC Centre for Global Infectious Disease Analysis Department of Infectious Disease Epidemiology Imperial College London London UK; ^2^ Biomedical Research and Training Institute Harare Zimbabwe; ^3^ Independent consultant Innsbruck Austria

**Keywords:** sexual behaviour, condoms, temporal trends, eastern and southern Africa, sexually transmitted infections, HIV prevention

## Abstract

**Introduction:**

Sexual behaviour change contributed to reductions in HIV incidence in eastern and southern Africa between 1990 and 2010. More recently, there are indications that non‐regular partnerships have increased. However, the effect of these increases on population‐level risks for HIV and other sexually transmitted infections could have been reduced by simultaneous increases in condom use. We describe recent trends in sexual behaviour and condom use within the region and assess their combined effects on population levels of sexual risk.

**Methods:**

Nationally representative Demographic and Health Survey data on sexually active males and females (15 to 49 years) were used for 11 eastern and southern African countries (≥3 surveys for each country; 1999 to 2016) to describe trends in sexual behaviour (multiple, non‐regular, and casual sexual partnerships; condom use; age at first sex). Logistic regressions tested for statistical significance of changes. Analyses were stratified by sex.

**Results:**

Recent increases in multiple, non‐regular, and/or casual partnerships can be found for males in 10 countries and, for females, in nine countries; five countries exhibited recent decreases in age of sexual debut. Reduction in sex without condoms with non‐regular partners was observed in six countries for males and eight for females. Changes in the proportion of the overall population reporting condomless sex with non‐regular partners varied between countries, with declines in six countries and increases in three.

**Conclusions:**

Extensive change in sexual behaviour occurred across eastern and southern Africa during the period of scale‐up of antiretroviral therapy programmes. This includes increasing multiple and non‐regular partnerships, but their potential effects on population‐level sexual risks were often offset by parallel increases in condom use. Strengthening condom programmes and reintegrating communication about behavioural dimensions into combination prevention programmes could help countries to meet international targets for reductions in HIV incidence.

## 1 Introduction

In 2017, more than half of the world's people living with HIV (PLHIV) lived in eastern and southern Africa (19.6 million), 800,000 people became newly HIV‐infected (44% of global infections), and 380,000 PLHIV died from AIDS‐related causes (40% of global deaths) 1. Eastern and southern Africa has also been the stage for some of the greatest accomplishments in the response to HIV/AIDS. Adult HIV prevalence declined by one quarter since peaking around 2000 and HIV incidence more than halved since the late 1990s 1. Empirical and modelling studies suggests that widespread change in sexual behaviour contributed to these declines, including delayed sexual debut, reductions in numbers of sexual partners and non‐regular partnerships, and – particularly later during the epidemic – increased condom use 2, 3, 4, 5, 6, 7.

Since antiretroviral therapy (ART) has become available in high‐income countries in the mid‐1990s, extensive increases in potentially risky sexual behaviour among men who have sex with men (MSM) have been observed 8. The availability of ART may have created optimistic beliefs regarding the severity of HIV infections and, given the evidence that virally suppressed PLHIV cannot transmit HIV (Undetectable=Untransmissable) 9, risks of transmission during condomless sex, leading to disinhibition of sexual behaviour 10, 11, 12, 13. Associations between ART‐related optimistic beliefs and potentially risky sexual behaviour among MSM in high‐income countries have been found in empirical studies 13, although causality and extent of such treatment optimism contributing to changes in sexual behaviour among MSM are debated 14.

In low‐ and middle‐income countries, ART has become increasingly available since the early 2000s, and, in eastern and southern Africa, 66% of PLHIV are now on treatment 1. Fears that PLHIV would engage in more potentially risky behaviour after treatment initiation are unfounded by empirical evidence 15, 16. However, it is unclear how far increasing ART availability and decreasing HIV‐linked mortality have impacted beliefs about HIV in low‐ and middle‐income countries and led to changes in sexual behaviour in the general population. Similar to findings among MSM in high‐income countries, treatment optimism could lead to rises in sexual behaviours associated with risks for HIV and other sexually transmitted infections (STIs) (including multiple, non‐regular and casual partnerships 17, 18, 19). Early mathematical modelling studies, conducted when ART first became more widely available in developing countries, indicated that disinhibition of sexual behaviour could lead to increases in HIV incidence 20, 21, although modelling studies of the impact of ART on HIV incidence tend to assume no changes in sexual behaviour 22. Few empirical studies have investigated changes in sexual behaviour in relation to optimistic beliefs about HIV severity or transmission in developing countries 13. A limited number of studies in eastern and southern Africa found optimistic beliefs about HIV due to ART associated with risky sexual behaviour 23, 24, 25. A review and analysis of nationally representative data from sub‐Saharan Africa found increasing multiple and transactional sexual partnerships but also decreasing condomless sexual intercourse 26. It is therefore unclear whether and to what extent changes in sexual behaviour have led to increased numbers of people exposed to elevated HIV/STI risks. In this article, we present analyses of repeated, nationally representative surveys from 11 countries in eastern and southern Africa to improve our understanding of the impact recent changes in sexual behaviour on population‐level patterns of sexual risk by (1) incorporating more recent data that may reflect the effects of higher levels of ART coverage achieved recently and (2) analysing a broader set of sexual behaviour measures, including measures of HIV/STI risks in the population as a whole.

## 2 Methods

### 2.1 Data and measures

Demographic and Health Survey (DHS) and AIDS Indicator Survey (AIS) data from countries in eastern and southern Africa (defined by UNAIDS 1) were used. These are nationally representative, standardized surveys with high response rates 27, collecting information on various population and health topics. Analyses were restricted to phase four DHS (1997 to 2003) onwards as data on key sexual behaviour measures were not collected in earlier surveys. Countries were included if at least three surveys were available and at least one was implemented after 2009 (Ethiopia, Kenya, Lesotho, Malawi, Mozambique, Namibia, Rwanda, Tanzania, Uganda, Zambia and Zimbabwe), with included surveys completed between 1999 and 2016. Data were restricted to individuals aged 15 to 49 years reporting sexual intercourse in the past 12 months. Sample sizes varied between 1400 and 10,100 for males and 3100 and 18,300 for females. Trends in specific sexual behaviours may be affected by changing proportions of individuals being sexually active 28. These biases were considered in sensitivity analyses but no indications for these were found as there was limited change in the sizes of sexually active populations (See Supporting Information, Section 6).

Sexual behaviour measures in this study are described in Table 1. Primary sexual behaviour measures include multiple partnerships, non‐regular partnerships, condomless sex with the last non‐regular partner among those reporting non‐regular partners (condomless non‐regular sex), and condomless sex with the last non‐regular partner among everyone who had sex in the past 12 months (population‐level condomless non‐regular sex). Population‐level condomless non‐regular sex captures trends in both non‐regular partnerships and condom use within those partnerships, evaluating in how far potential increases in non‐regular partnerships translated into increases in proportions of individuals exposed to elevated HIV/STI risks in the population as a whole. Secondary analyses included casual partnerships, condomless casual sex and age at first sex (Supporting Information, Section 4). Condom use in all relationships, including long‐term partners, was not considered as these partnerships are characterized by lower HIV/STI risks and condom use in stable partnerships tends to be low. Details on surveys and measures are provided in the Supporting Information (Section 1).

**Table 1:** Definitions of primary and secondary sexual behaviour measures of this study

**Primary sexual behaviour measures**	
Multiple partnerships	Having more than one sexual partner in the past 12 months.
Non‐regular partnerships	Having at least one sexual partner in the past 12 months who the participant was not married to or did not live with. This includes all non‐spousal, non‐cohabiting partners, including casual acquaintances and sex workers/clients.
Condomless non‐regular sex	Not using a condom during the last sexual intercourse with a non‐regular partner in the past 12 months among everyone who had a non‐regular partner in the past 12 months.
Population‐level condomless non‐regular sex	Not using a condom during the last sexual intercourse with a non‐regular partner in the past 12 months among everyone who had sex in the past 12 months. This measure covers the whole sexually active population, including those who did not have non‐regular partners.
**Secondary sexual behaviour measures**	
Casual partnerships	Having at least one casual sexual partner in the past 12 months. This includes casual acquaintances and sex workers/clients. Casual partnerships represent a subset of non‐regular partnerships and are likely to be characterized by high HIV/STI infection risks.
Condomless casual sex	Not using a condom during the last sexual intercourse with a casual partner in the past 12 months among everyone who had a casual partner in the past 12 months.
Age at first sex	Age at which the participant first had sexual intercourse among those aged 20 to 29 years.

For further information on measures, including on survey questions and changes in measurement over time, see Supporting Information.

### 2.2 Analysis

Proportions and 95% confidence intervals (CIs) were calculated for each sexual behaviour for each survey to describe trends over time in every country. Statistical significance of changes in sexual behaviours was evaluated by estimating logistic regressions with each behaviour as the outcome and an indicator for the survey and age (continuous) as independent variables. Regressions were restricted to two consecutive surveys, so one survey was compared to the preceding one. For each outcome, a significant recent change was defined as a statistically significant difference (*p *<* *0.05) in the most recent survey compared to the previous survey. For countries where the two most recent surveys were conducted after 2009 and no significant difference was determined between these two surveys (*p *≥* *0.10), a significant change was also considered to have occurred if there was a significant difference between the earlier of the two most recent surveys and the last survey preceding 2010 (*p *<* *0.05). All analyses were implemented separately by sex, survey sampling weights were applied to create nationally representative results, and clustering of individuals due to sample design was accounted for (see Supporting Information, Section 1). Where possible (five countries), analyses were also stratified by HIV status (Supporting Information, Section 5).

## 3 Results

**Figure 1:**
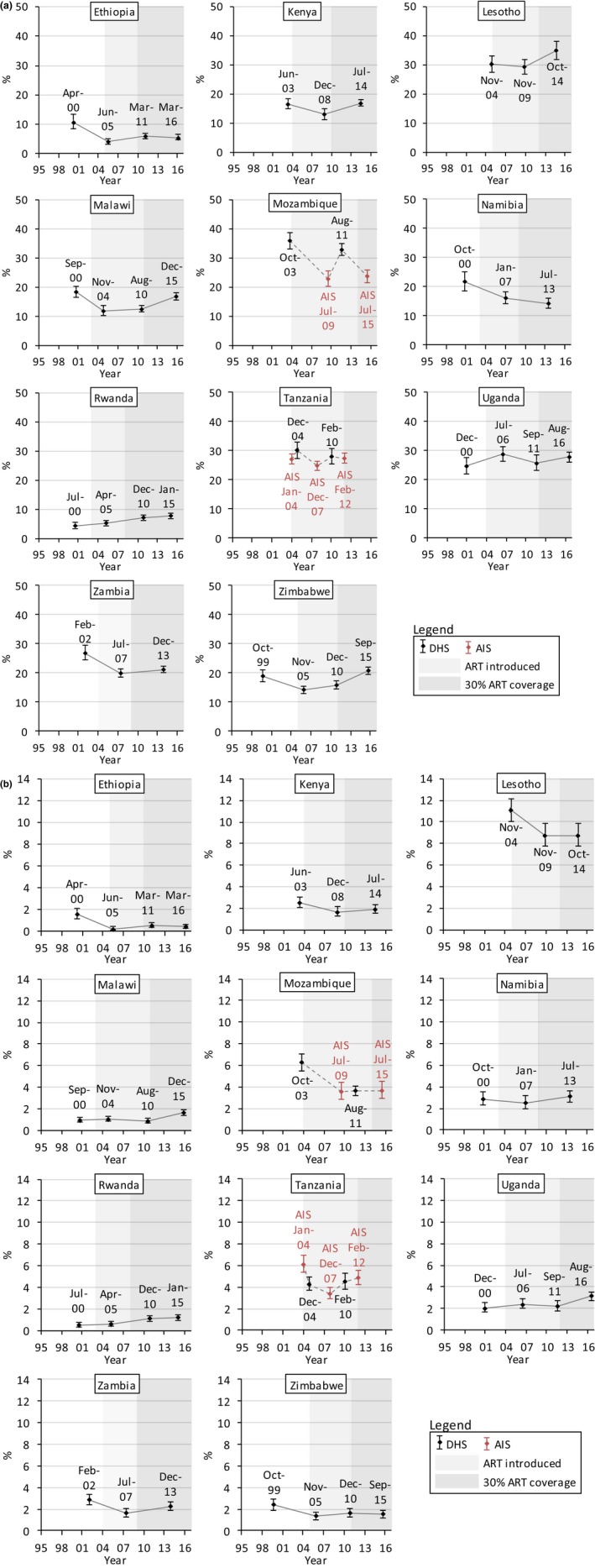
Trends in multiple sexual partnerships among (a) males and (b) females (15 to 49 years), eastern and southern Africa. Multiple sexual partnerships were defined as reporting more than one sexual partner in the past 12 months among everyone who reported sex in the past 12 months. Dates refer to the mid‐points of the survey data collection period. Data from AIS are indicated in red. Data from different survey types are linked with dashed lines. Shaded areas indicate the years in which ART was introduced into the public healthcare sector in each country and from when 30% of adult PLHIV (15 + years) were in treatment (disregarding treatment eligibility criteria; see Supporting Information, Section 2).

**Figure 2:**
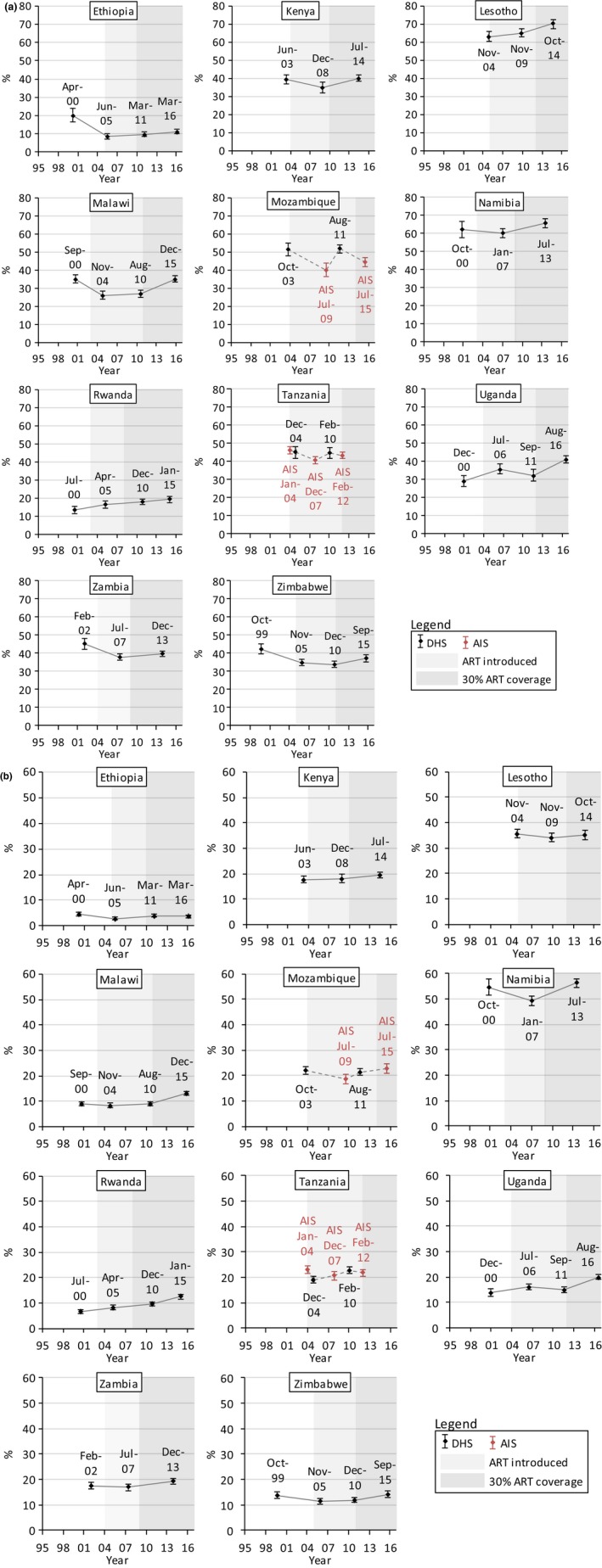
Trends in non‐regular sexual partnerships among (a) males and (b) females (15 to 49 years), eastern and southern Africa. Non‐regular sexual partnerships were defined as reporting at least one sexual partner in the past 12 months who the participant was not married to and did not live with among everyone who reported sex in the past 12 months. Dates refer to the mid‐points of the survey data collection period. Data from AIS are indicated in red. Data from different survey types are linked with dashed lines. Shaded areas indicate the years in which ART was introduced into the public healthcare sector in each country and from when 30% of adult PLHIV (15 + years) were in treatment (disregarding treatment eligibility criteria; see Supporting Information, Section 2).

**Figure 3:**
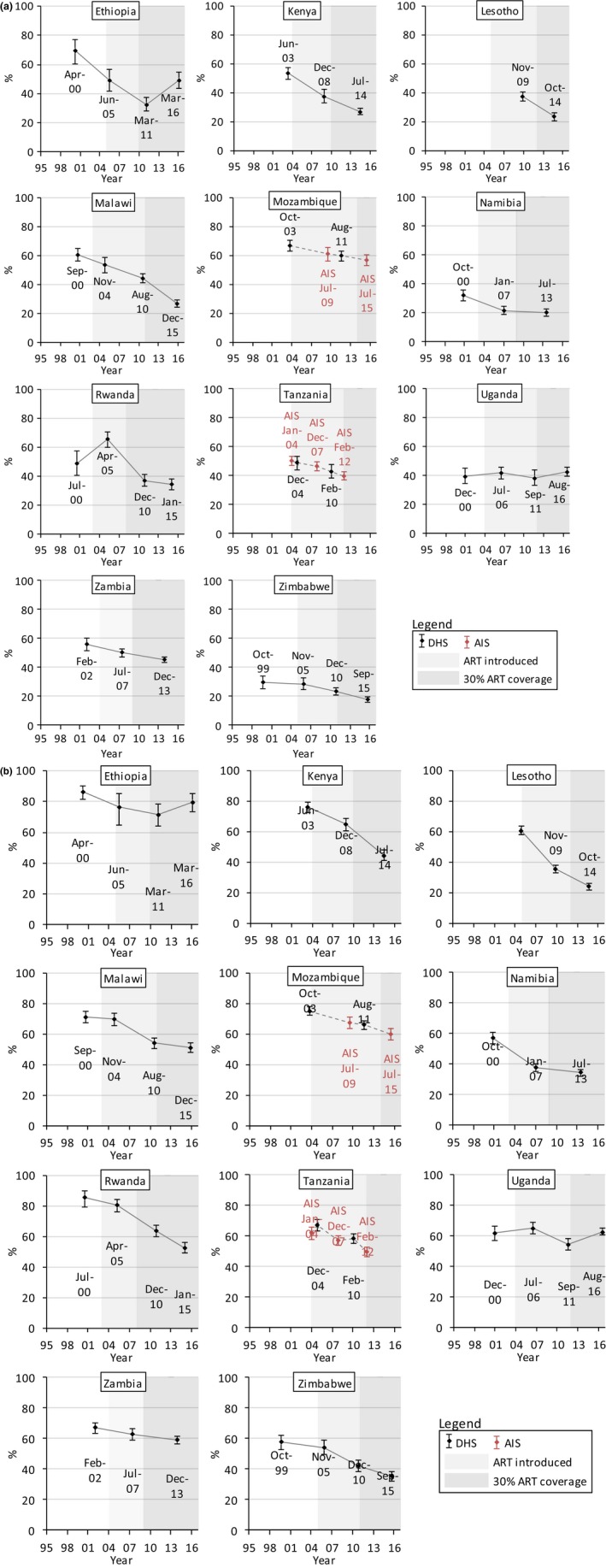
Trends in condomless sex with the last non‐regular partner among (a) males and (b) females (15 to 49 years) who had non‐regular partners in the past 12 months, eastern and southern Africa. The condom use refers to the last sexual intercourse in the past 12 months with a partner who the participant was not married to and did not live with among everyone who had such a non‐regular partner in the past 12 months. Dates refer to the mid‐points of the survey data collection period. Data from AIS are indicated in red. Data from different survey types are linked with dashed lines. Shaded areas indicate the years in which ART was introduced into the public healthcare sector in each country and from when 30% of adult PLHIV (15 + years) were in treatment (disregarding treatment eligibility criteria; see Supporting Information, Section 2).

**Figure 4:**
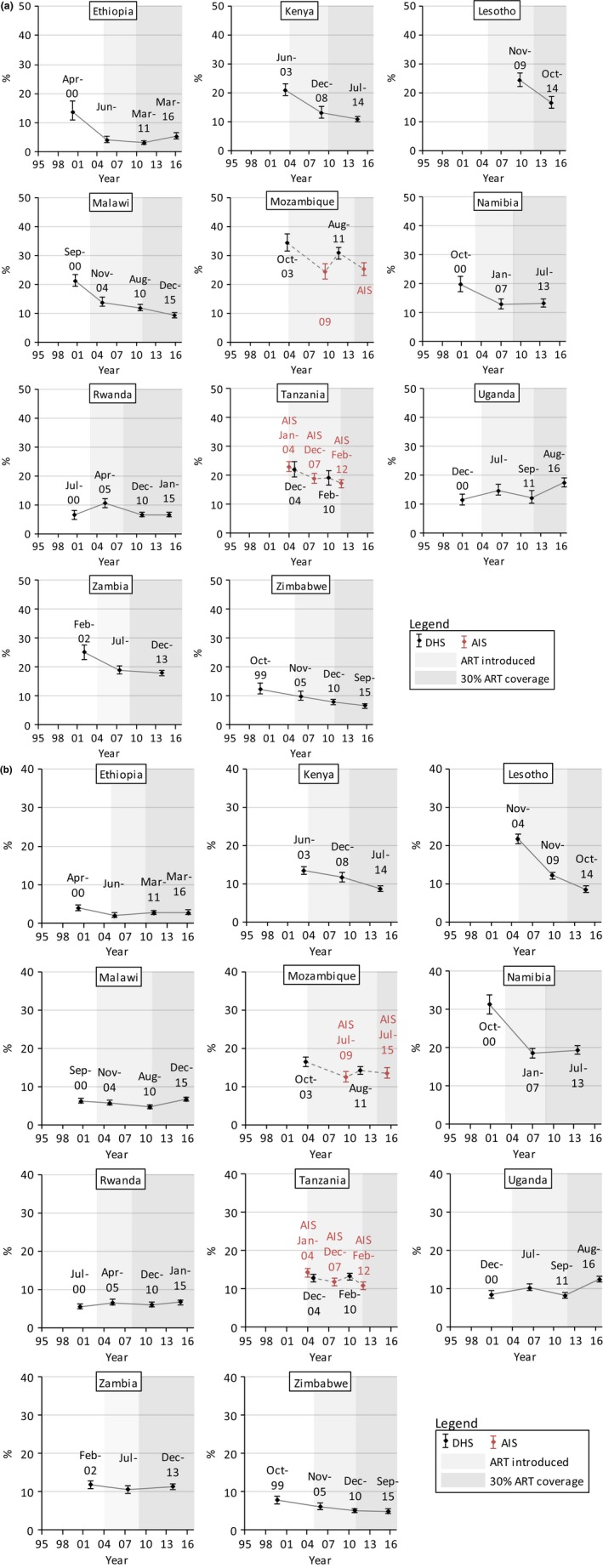
Trends in condomless sex with the last non‐regular partner among (a) males and (b) females (15 to 49 years) who had sex in the past 12 months, eastern and southern Africa. The condom use refers to the last sexual intercourse in the past 12 months with a partner who the participant was not married to and did not live with among everyone who had any sexual partner in the past 12 months. Dates refer to the mid‐points of the survey data collection period. Data from AIS are indicated in red. Data from different survey types are linked with dashed lines. Shaded areas indicate the years in which ART was introduced into the public healthcare sector in each country and from when 30% of adult PLHIV (15 + years) were in treatment (disregarding treatment eligibility criteria; see Supporting Information, Section 2).

**Figure 5:**
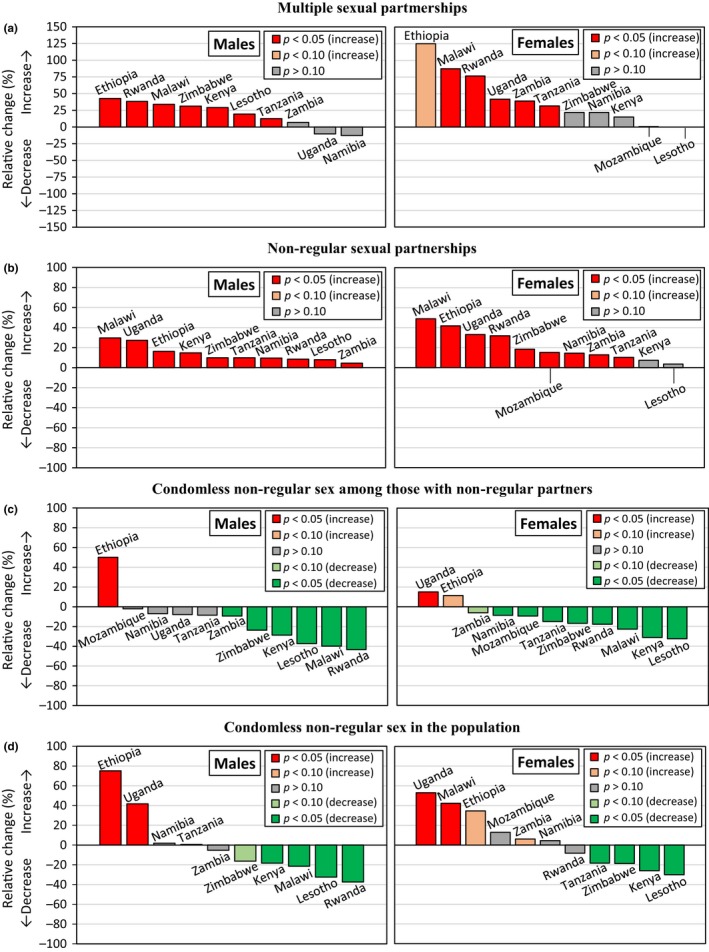
Relative recent change in sexual behaviour measures among males and females (15 to 49 years), eastern and southern Africa. (a) Multiple partnerships among everyone who had sex in the past 12 months. (b) Non‐regular partnerships among everyone who had sex in the past 12 months. (c) Condomless sex with the last non‐regular partner among everyone reporting a non‐regular partner in the past 12 months. (d) Condomless sex with the last non‐regular partner among everyone who had sex in the past 12 months. The comparison refers to the two most recent surveys. Where there were more than two surveys from the year 2010 and no difference between the most recent and the preceding one (*p *≥* *0.10), the comparison refers to the earliest of the surveys from 2010 and the survey preceding 2010. Significance was determined in logistic regressions, adjusting for age. See Table 2.

Figures 1, 2, 3, and 4 show trends in multiple partnerships, non‐regular partnerships, condomless non‐regular sex, and population‐level condomless non‐regular sex. Additional data in table format are available in the Supporting Information (see Section 3 and Tables S3 to S6). These tables include *p*‐values from regressions referred to throughout the text, and Figure 5 shows relative recent change in these measures. Results for casual partnerships, condomless casual sex and age at first sex are provided in the Supporting Information (Section 4). Table 2 provides an overall summary.

**Table 2:**
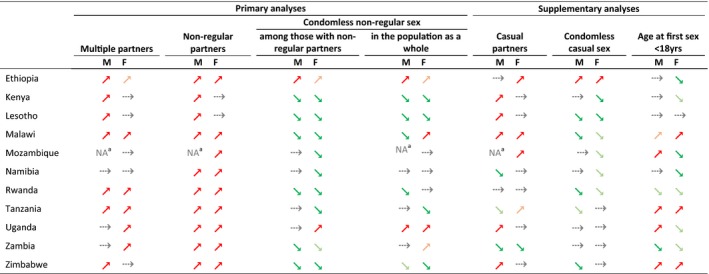
Recent changes in sexual behaviour in eastern and southern Africa

Symbols indicate recent trends in risky sexual behaviour and their statistical significance among males (M) and females (F). Significance was determined in logistic regressions, adjusting for age, comparing consecutive surveys. A significant recent increase (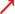) or significant recent decrease (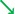) was observed if the most recent survey was significantly different (*p *<* *0.05) to the preceding survey. A non‐significant recent upward trend (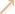) or non‐significant recent downward trend (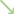) was observed if statistical significance was approached (0.05 < *p *<* *0.10). No recent change (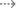) was observed if the most recent survey was not significantly different (*p *≥* *0.10) to the preceding survey. Where there were more than two surveys from the year 2010 and no difference between the most recent and the preceding one (*p *≥* *0.10), the comparison refers to the earliest of the surveys from 2010 and the survey preceding 2010.

Multiple partners: Reporting more than one sexual partner in the past 12 months among everyone who had sex in the past 12 months. Non‐regular partners: Reporting at least one non‐married, non‐cohabiting sexual partner in the past 12 months among everyone who had sex in the past 12 months. Condomless non‐regular sex: Reporting not using a condom during last sexual intercourse with a non‐regular partner among (a) everyone who had a non‐regular partner in the past 12 months; (b) everyone who had sex in the past 12 months. Casual partners: Reporting at least one casual sexual partner in the past 12 months among everyone who had sex in the past 12 months. Condomless casual sex: Reporting not using a condom during last sexual intercourse with a casual partner among everyone who had a casual partner in the past 12 months. Age at first sex <18 years: Age at first sex reported to be before the age of 18 years among everyone who had sex and was aged 20 to 29 years.

The trends in some sexual behaviour measures in Mozambique for males was unclear given strongly varying results between recent surveys.

### 3.1 Multiple partnerships

Among males (Figure 1a), reporting of multiple partnerships increased by 30% between the most recent surveys in Kenya (*p *=* *0.0012), Malawi (*p *<* *0.0001) and Zimbabwe (*p *<* *0.0001), reaching 16.9% (95% CI = 15.8% to 18.0%), 16.8% (15.5% to 18.2%) and 20.7% (19.4% to 22.0%) respectively. Recent increases were also observed in Tanzania (*p *=* *0.0472) and Lesotho (*p *=* *0.0045), reaching high levels of 27.4% (25.8% to 29.2%) and 34.9% (31.7% to 38.2%) respectively. In Rwanda, levels of multiple partnership were lower but increased by 38% between 2005 (5.07% (4.13% to 6.20%)) and 2010 (7.01% (6.19% to 7.92%)) (*p *=* *0.0084). Ethiopia had low levels of multiple partnerships (5.50% (4.61% to 6.55%) in 2016) but a significant increase occurred between 2005 and 2011 (*p *=* *0.0095). The trend in Mozambique was unclear due to strong variation between AIS and DHS results.

Females were less likely to report multiple partners but increases were observed in a number of countries (Figure 1b). Multiple partnerships doubled at low levels in Malawi between 2010 (0.88% (0.71% to 1.09%)) and 2015/16 (1.65% (1.42% to 1.92%)) (*p *<* *0.0001) and in Ethiopia between 2005 (0.24% (0.11% to 0.50%)) and 2011 (0.54% (0.35% to 0.83%)) (*p *=* *0.0598). In Rwanda, an upward trend was observed (1.18% (0.94% to 1.46%) in 2014/15) (*p *=* *0.0068, between 2005 and 2010). Higher levels of multiple partnerships were observed in Tanzania, with an increase from 3.41% (2.92% to 3.98%) in 2007/08 to 4.49% (3.83% to 5.26%) in 2010 (*p *=* *0.0142), and 40% increases occurred in Zambia (*p *=* *0.0145) and Uganda (*p *=* *0.0057). In Lesotho levels were stable but high (8.76% (7.75% to 9.88%) in 2014).

### 3.2 Non‐regular partnerships

Significant recent increases in non‐regular partnerships among males were observed in all countries except Mozambique (Figure 2a), where the trend was unclear. Increases of nearly 30% were observed in Malawi (35.0% (33.2% to 36.9%) in 2015/16) (*p *<* *0.0001) and Uganda (41.0% (39.0% to 43.1%) in 2016) (*p *=* *0.0002). The significant recent increases (*p *<* *0.0001) in Namibia and Lesotho led to high levels of 65.8%(63.4% to 68.3%) and 70.1% (67.6% to 72.5%), respectively.

Among females, similarly, there were recent increases in non‐regular partnerships in nearly all countries (Figure 2b). Strong increases (*p *<* *0.0001) of nearly 50% were observed in Malawi (reaching 13.2% (12.4% to 14.0%) in 2015) and of 33% in Uganda (20.1% (19.1% to 21.1%) in 2016). In Rwanda, there was a long‐term upward trend, with levels in 2014/15 (12.4% (11.6% to 13.3%)) 32% higher than in 2010 (9.43% (8.68% to 10.2%)) (*p *<* *0.0001). In Ethiopia, levels of non‐regular partnerships were low but increased by 42% between 2005 (2.80% (2.22% to 3.53%)) and 2011 (3.97% (3.38% to 4.64%)) (*p *=* *0.0128). Non‐regular partnerships increased at high levels in Namibia (56.3% (54.6% to 58.0%) in 2013) (*p *<* *0.0001) and remained stable and high in Lesotho (35.3% (33.4% to 37.2%) in 2014).

Trends in casual partnerships were similar to non‐regular partnerships in many countries (Table 2), although declines were observed in Zambia and Namibia. See Supporting Information, Section 4.

### 3.3 Condom use among those reporting non‐regular partners

In most countries, there were long‐term decreases in condomless non‐regular sex among those with non‐regular partners (Table 2; Figures 3a,b). In Ethiopia, there were recent increases in condomless non‐regular sex among males (*p *<* *0.0001) and females (*p *=* *0.0672) (reaching 49.0% (43.5% to 54.6%) and 79.6% (73.2% to 84.9%), respectively, in 2016). Among females, condomless non‐regular sex increased in Uganda to 62.7% (60.5% to 64.9%) in 2016 (*p *=* *0.0005). However, despite generally decreasing trends, condomless non‐regular sex was above 30% in six countries for males (highest: Mozambique (56.7%)) and above 50% in six countries for females (highest: Ethiopia (79.6%)). Trends in condom use during casual sex tended to be similar (see Supporting Information, Section 4).

### 3.4 Population‐level condomless non‐regular sex

Trends in population‐level condomless non‐regular sex among males (Figure 4a) tended to be similar to trends in condomless non‐regular sex among those reporting non‐regular partners. Population‐level condomless non‐regular sex increased by 75% in Ethiopia (5.50% (4.54% to 6.67%) in 2016) (*p *<* *0.0001) and by 42% in Uganda (17.5% (16.1% to 19.1%) in 2016) (*p *=* *0.0021). Despite increasing non‐regular partnerships, there were no recent changes in population‐level condomless non‐regular sex in Namibia, Tanzania and Zambia, and there were decreases in Zimbabwe, Kenya, Malawi, Lesotho and Rwanda. In Malawi, particularly, there was a strong increase in non‐regular partnerships of 30% but a 21% decrease in population‐level condomless non‐regular sex (reaching 9.33% (8.43% to 10.3%) in 2015/16) (*p *<* *0.0001).

For females, trends in population‐level condomless non‐regular sex (Figure 4b) tended to be more different to trends in condomless non‐regular sex. In Uganda, population‐level condomless non‐regular sex increased by 52% (from 8.21% (7.43% to 9.09%) in 2011 to 12.6% (11.9% to 13.3%) in 2016) (*p *<* *0.0001). In Malawi, there was a significant increase of 42% in population‐level condomless non‐regular sex (reaching 6.75% (6.21% to 7.32%)) (*p *<* *0.0001) despite the observed reduction in condomless non‐regular sex among those with non‐regular partners. In Kenya and Lesotho, where there were no significant changes in non‐regular partnerships, significant decreases were found in population‐level condomless non‐regular sex (reaching 8.65% (7.93% to 9.42%) in 2014 in Kenya and 8.46% (7.58% to 9.44%) in 2014 in Lesotho) (*p *<* *0.0001). Although non‐regular partnerships increased, population‐level condomless non‐regular sex decreased in Zimbabwe and Tanzania and remained stable in Mozambique, Zambia, Namibia and Rwanda.

### 3.5 Age at first sex

Reporting of age at first sex before 18 years among those aged 20 to 29 years was stable in many countries or followed declining trends (Table 2), particularly between earlier surveys. Exceptions to this are Malawi, Tanzania and Zimbabwe, where there were recent increases in early sexual debut among both sexes, as well as Mozambique and Uganda, where early sexual debut increased among males (Supporting Information, Section 4).

## 4 Discussion

In this study of nationally representative data on sexually active males and females aged 15 to 49 years from 11 countries in eastern and southern Africa, we document widespread recent change in sexual behaviour. Significant recent increase in at least one of multiple or non‐regular partnerships occurred in 10 countries for males and nine countries for females; casual partnerships also increased in several countries. Early sexual debut increased in five countries and declined in four. These changes in partnership patterns and sexual debut were accompanied by widespread and long‐term decreases in condomless sex. Population‐level condomless non‐regular sex – capturing trends in non‐regular partnerships and condom use within these partnerships – decreased in Kenya, Lesotho, Malawi (males), Tanzania (females) and Zimbabwe (females), but increased in Ethiopia (males), Malawi (females) and Uganda. Similar patterns were found for HIV‐positive and HIV‐negative individuals (Supporting Information, Section 5).

### 4.1 Implications for population‐level sexual risk

Results of this study suggest that increases in certain behaviours associated with increased risk of HIV/STI infection – multiple and non‐regular partnerships – may not translate into increasing proportions of the sexually active population as a whole engaging in potentially risky sex. For example, proportions of males in Malawi reporting non‐regular partnerships increased strongly between 2010 and 2015/16 but proportions of sexually active males in the population engaging in condomless non‐regular sex decreased due to increased condom use. Conversely, decreasing condomless sex in non‐regular partnerships may not translate into lower proportions in the population engaging in condomless non‐regular sex due to parallel increases in non‐regular partnerships. For instance, females in Rwanda reported markedly more condom use in non‐regular partnerships in 2014/15 compared to 2010, but there was only a small decrease in condomless non‐regular sex in the population.

This study underlines that behaviours considered “risky” – having multiple or non‐regular partners – are not necessarily “risky” in themselves and that increases in these behaviours could be considered unproblematic if appropriate protective measures are taken (condoms, pre‐exposure prophylaxis (PrEP), ART for PLHIV). Considered in isolation, changes in multiple and non‐regular partnerships may lead to incorrect conclusions regarding trends in sexual risk. In some countries (e.g. Ethiopia and Uganda), trends in these partnerships suggest increased population‐level sexual risks for HIV/STIs (as indicated by a previous study 29). In other countries (e.g. Namibia and Zambia), increases in non‐regular partnerships have not led to population‐level increases in condomless non‐regular sex, but, equally, can be seen as having prevented rising levels of condom use from helping to bring down levels of HIV incidence. Where increases in early sexual debut have occurred, these could have had a similar effect.

While having multiple or non‐regular sexual partners may not lead to increased HIV/STI risks if protective measures are implemented perfectly, this is rarely the case in real‐life situations in eastern and southern Africa. Despite widely declining condomless sex, condom use in non‐regular partnerships was far from complete, with less than 70% of males and less than 50% of females reporting condom use with non‐regular (and casual) partners in most countries. ART and PrEP may substantially reduce HIV infection risks of among people engaging in condomless sex. However, while ART coverage rose in eastern and southern Africa from virtually zero before 2003 to 66% in 2017 1, significant gaps remain and high levels of coverage may not translate into population‐level reductions in HIV incidence 30. PrEP availability remains low in sub‐Saharan Africa 1. Therefore, people who have condomless sex with multiple and non‐regular sexual partners continue to be at increased HIV/STI risks.

### 4.2 Possible reasons for changes in sexual behaviour

In most countries, increases in multiple and non‐regular partnerships occurred after periods of declining or stable levels in these behaviours, although trends in the late 1980s and 1990s were not captured in this analysis. This reflects that initial behavioural responses to the HIV epidemic included delayed sexual debut and reductions in sexual partners, with condom use becoming more important later during the epidemic 4. Condom use could have increasingly replaced other behavioural strategies to reduce HIV infection risks, thus – together with general perceptions about increasing condom use – contributing to populations returning to levels of sexual activity that occurred prior to the epidemic. Moreover, increasing ART coverage primarily contributed to a 62% drop in HIV‐related deaths since the peak in 2004 1. Behaviour change in the 1990s and early 2000s has partially been attributed to common personal experience of HIV mortality 31. ART has turned a previously terminal illness into a manageable chronic disease. HIV/AIDS may thus be considered less severe, reducing motivation to engage in protective behaviour 32, and reduced the salience of HIV 33. ART availability and declining HIV‐related mortality could therefore have further disinhibited sexual activity (in addition to hypothesized non‐psycho‐social pathways through which ART may influence behaviour – see 34).

Effects of increasing condom use and ART coverage on sexual behaviour were not directly evaluated. Beliefs about reduced severity of HIV infection could have developed gradually over time, even before higher levels of ART coverage were achieved. Nevertheless, there is limited evidence for optimistic beliefs about HIV due to ART. Cross‐sectional studies in Uganda 23, Kenya 24 and Malawi 25 found associations between optimistic beliefs and potentially risky sexual behaviour, but such beliefs could be used to justify behaviour in hindsight. Moreover, these studies found optimistic beliefs to be uncommon. Studies among MSM in high‐income settings similarly found fractions of risky behaviour attributable to optimistic beliefs to be small 14. Unrelated to ART, there may be a general normalization of HIV/AIDS – an epidemic spanning more than three decades – that has contributed to complacency, as has been observed for other diseases 35.

Changes in international funding priorities for the HIV response may have contributed to observed changes in sexual behaviour too. International funding increased substantially until 2010 and stabilized thereafter 36, 37. HIV prevention funding and programmatic focus may have shifted from behaviour change to biomedical interventions such as treatment‐as‐prevention, prevent‐mother‐to‐child‐transmission and voluntary medical male circumcision (VMMC) since 2011 (see, for example, the “Blueprint” of the U.S. President's Emergency Plan for AIDS Relief 38). With multiple and non‐regular partnerships starting to increase after 2005 in some countries and more consistently across countries after 2010, reductions in funding could have amplified an emerging trend. It is therefore concerning that funding for condom programmes may also have been reduced in the last few years 37 as this could exacerbate gaps in provision and lead to a slowing or even a reversal of the rising levels of condom use documented in this study.

Available HIV prevention tools have also become more diverse. However, PrEP became available too recently to impact observed behavioural trends, and for VMMC, promoted since the mid‐2000s 39, there is limited evidence for risk compensation 40. Moreover, broader socio‐demographic and economic changes could explain changing behaviour. For example, rising poverty may have contributed to changes in sexual behaviour in Zimbabwe in the 1990s 31. Patterns of condom use can also be influenced by fertility preferences and family planning services. However, patterns in these factors are likely to differ markedly between countries, while similar changes in sexual behaviour were observed across the region.

### 4.3 Limitations

Self‐reporting of sexual behaviour is subject to reporting biases. Non‐regular partnerships may be especially underreported by females. The DHS involves extensive interviewer training 27 and interviewers try to reduce reporting biases by building rapport with survey participants 41. However, sex differences in reporting of sexual behaviour have been described for DHS data 42 and is evident in this study that shows consistently higher levels of multiple and non‐regular partnerships among males. Levels of these partnerships reported by females are therefore likely to be underestimated. It is, however, unlikely that these biases changed strongly over short time periods (across countries) so that observed changes in sexual behaviour would purely be the result of changing reporting. This is particularly unlikely since the study populations have been exposed to HIV‐related messages for decades, so it is unlikely that reporting patterns would change in recent times. Therefore, these biases affect levels of sexual behaviours but not the interpretation of trends in behaviours within countries, particularly as the DHS aims for comparability across surveys 27. Similarly, condom use during last sexual intercourse may be over‐reported and not reflect condom use over longer time periods; however, again, these biases are unlikely to affect trends in condomless sex within countries.

Different patterns of reporting could explain variation in results between different survey types (DHS and AIS) among males in Mozambique. It may be that, in HIV‐specific surveys, participants feel stronger pressures to underreport certain sexual behaviours; there is also more time in a DHS to build trust between interviewer and participant. While it is unclear why this should affect responses in Mozambique in particular, AIS were analysed for only two countries, so general conclusions are not affected by using two survey types.

Type of sexual intercourse (vaginal, anal or oral) was not specified in survey measurements. HIV infection risks are higher during anal intercourse compared with vaginal intercourse 43, 44 and contributions of heterosexual anal intercourse to new HIV infections could be substantial 45, 46. Trends in prevalence and frequency of heterosexual anal intercourse may affect trends in population‐level sexual risks, which further depends on condom use during anal intercourse. This is may be to be lower than for vaginal intercourse 47, although a systematic review of South Africa indicated equal or higher condom use during anal intercourse 48. Some studies suggest that heterosexual anal intercourse has become more common over time but, generally, trends in heterosexual anal intercourse in sub‐Saharan Africa are poorly understood 47, 49 and impacts of different types of sexual intercourse on population‐level sexual risks could not be evaluated in this study.

## 5 Conclusions

We document epidemiologically relevant changes in sexual behaviours across eastern and southern Africa, including increases in multiple and non‐regular partnerships – behaviours associated with elevated risk of HIV infection. After years of declines, populations may return to pre‐epidemic levels in these behaviours, possibly reflecting increasing levels of condom use and complacency due to treatment optimism and normalization of HIV/AIDS, although underlying reasons are poorly understood and may differ between populations, including different age groups. Impact of increases in these behaviours on population‐level sexual risks is likely to vary by country. Despite increasing non‐regular partnerships, population‐level condomless non‐regular sex may decline or remain stable. Multiple and non‐regular partnerships are thus not inherently problematic, but increases in these behaviours may create challenges for HIV prevention and affect other sexual and reproductive health outcomes. This is most obvious in countries where population‐level condomless non‐regular sex increased, but even where this remained stable increasing condom use may not translate into reductions in HIV incidence. The public health implications of these behavioural trends also depend on prevalence of multiple and non‐regular partnerships and increases in these behaviours may create more challenges in countries where they are more common. However, many countries in eastern and southern Africa are off track to meet international targets of reducing HIV incidence 50. Continuing high incidence threatens the sustainability of HIV treatment programmes. Further improvements in HIV prevention efforts are therefore critical, including ensuring political leadership and securing adequate financing. Rather than deprioritizing behaviour change promotion, behavioural dimensions should be integrated into combination prevention programmes – particularly in the eastern and southern African region that has shown how behaviour change can impact the epidemic and which is set to play a central role in the efforts to end AIDS.

## Competing interests

Simon Gregson declares shareholding in pharmaceutical companies (GSK and Astra Zeneca). All other authors have no potential conflicts of interests to declare.

## Authors’ contributions

All authors contributed to the design of the study. C.B. conducted preliminary analyses that formed the basis of the main analysis. R.S. conducted the main analysis with input from C.B. and S.G. The manuscript was drafted by R.S. All authors contributed to the interpretation of results and the manuscript. All authors approved the final manuscript.

## Supporting information


**Table S1.** Description and data availability for all included surveys.
**Table S2.** Availability of ART in the eastern and southern African countries included in the analysis.
**Table S3.** Multiple sexual partnerships, eastern and southern Africa.
**Table S4.** Non‐regular sexual partnerships, eastern and southern Africa.
**Table S5.** Condomless non‐regular sexual intercourse among everyone with non‐regular partners, eastern and southern Africa.
**Table S6.** Condomless non‐regular sexual intercourse among everyone who had a sexual partner in the past 12 months, eastern and southern Africa.
**Table S7.** Casual sexual partnerships, eastern and southern Africa.
**Table S8.** Condomless casual sexual intercourse among everyone who had a casual sexual partner in the past 12 months, eastern and southern Africa.
**Table S9.** Age at first sex before the age of 18 years, eastern and southern Africa.
**Table S10.** Interaction of sexual risk behaviour and HIV status, eastern and southern Africa.
**Table S11.** Having ever had sex, eastern and southern Africa.
**Table S12.** Multiple sexual partnerships (including those who have never had sex before), eastern and southern Africa.
**Table S13.** Non‐regular sexual partnerships (including those who have never had sex before), eastern and southern Africa.
**Table S14.** Casual sexual partnerships (including those who have never had sex before), eastern and southern Africa.
**Table S15.** Age at first sex before the age of 18 years (including those who have never had sex before), eastern and southern Africa.
**Figure S1.** Trends in casual sexual partnerships among (a) males and (b) females (15 to 49 years), eastern and southern Africa. Casual sexual partnerships were defined as reporting at least one casual sexual partner in the past 12. Dates refer to the mid‐points of the survey data collection period. Data from AIS are indicated in red. Data from different survey types are linked with dashed lines. Shaded areas indicate the years in which ART was introduced into the public healthcare sector in each country and from when 30% of adult PLHIV (15 + years) were in treatment (disregarding treatment eligibility criteria; see Supporting Information, Section 2).
**Figure S2.** Trends in condomless sex with the last casual sexual partner among (a) males and (b) females (15 to 49 years), eastern and southern Africa. The condom use refers to the last sexual intercourse in the past 12 months with a casual partner among everyone who had such a casual in the past 12 months. Dates refer to the mid‐points of the survey data collection period. Data from AIS are indicated in red. Data from different survey types are linked with dashed lines. Shaded areas indicate the years in which ART was introduced into the public healthcare sector in each country and from when 30% of adult PLHIV (15 + years) were in treatment (disregarding treatment eligibility criteria; see Supporting Information, Section 2).
**Figure S3.** Trends in age at first sex before the age of 18 years among (a) males and (b) females (20 to 29 years), eastern and southern Africa. The sample included males aged 20 to 29 years who reported ever having had sexual intercourse. Dates refer to the mid‐points of the survey data collection period. Data from AIS are indicated in red. Data from different survey types are linked with dashed lines. Shaded areas indicate the years in which ART was introduced into the public healthcare sector in each country and from when 30% of adult PLHIV (15 + years) were in treatment (disregarding treatment eligibility criteria; see Supporting Information, Section 2).
**Figure S4.** Trends in multiple sexual partnerships among (a) males and (b) females (15 to 49 years) by HIV status, eastern and southern Africa. Multiple sexual partnerships were defined as reporting more than one sexual partner in the past 12 months. Dates refer to the mid‐points of the survey data collection period.
**Figure S5.** Trends in non‐regular sexual partnerships among (a) males and (b) females (15 to 49 years) by HIV status, eastern and southern Africa. Non‐regular sexual partnerships were defined as reporting at least one sexual partner in the past 12 months who the participant was not married to and did not live with. Dates refer to the mid‐points of the survey data collection period.
**Figure S6.** Trends in condom use with the last non‐regular sexual partners among (a) males and (b) females (15 to 49 years) by HIV status, eastern and southern Africa. The condom use refers to the last sexual intercourse in the past 12 months with a partner who the participant was not married to and did not live with among everyone who had such a non‐regular partner in the past 12 months. Dates refer to the mid‐points of the survey data collection period.
**Figure S7.** Trends in reporting of having had sex among (a) males and (b) females (12 to 49 years), eastern and southern Africa. The sample included males aged 15 to 49 years. Dates refer to the mid‐points of the survey data collection period. Data from AIS are indicated in red. Data from different survey types are linked with dashed lines. Shaded areas indicate the years in which ART was introduced into the public healthcare sector in each country and from when 30% of adult PLHIV (15 + years) were in treatment (disregarding treatment eligibility criteria; see Supporting Information, Section 2).
