# Supplementary material for: Widespread changes in sexual behaviour in eastern and southern Africa: Challenges to achieving global HIV targets? Longitudinal analyses of nationally representative surveys
Source: J Int AIDS Soc. 2019 Jun 21;22(6):e25329. doi: 10.1002/jia2.25329 (PMC6587908; doi:10.1002/jia2.25329)
Supplement: Supplementary file 1 — Table S1. Description and data availability for all included surveys. Table S2. Availability of ART in the eastern and southern African countries included in the analysis. Table S3. Multiple sexual partnerships, eastern and southern Africa. Table S4. Non‐regular sexual partnerships, eastern and southern Africa. Table S5. Condomless non‐regular sexual intercourse among everyone with non‐regular partners, eastern and southern Africa. Table S6. Condomless non‐regular sexual intercourse among everyone who had a sexual partner in the past 12 months, eastern and southern Africa. Table S7. Casual sexual partnerships, eastern and southern Africa. Table S8. Condomless casual sexual intercourse among everyone who had a casual sexual partner in the past 12 months, eastern and southern Africa. Table S9. Age at first sex before the age of 18 years, eastern and southern Africa. Table S10. Interaction of sexual risk behaviour and HIV status, eastern and southern Africa. Table S11. Having ever had sex, eastern and southern Africa. Table S12. Multiple sexual partnerships (including those who have never had sex before), eastern and southern Africa. Table S13. Non‐regular sexual partnerships (including those who have never had sex before), eastern and southern Africa. Table S14. Casual sexual partnerships (including those who have never had sex before), eastern and southern Africa. Table S15. Age at first sex before the age of 18 years (including those who have never had sex before), eastern and southern Africa. Figure S1. Trends in casual sexual partnerships among (a) males and (b) females (15 to 49 years), eastern and southern Africa. Casual sexual partnerships were defined as reporting at least one casual sexual partner in the past 12. Dates refer to the mid‐points of the survey data collection period. Data from AIS are indicated in red. Data from different survey types are linked with dashed lines. Shaded areas indicate the years in which ART was introduced into the public healthc [file JIA2-22-e25329-s001.pdf]

Widespread changes in sexual behaviour in eastern and southern Africa: Challenges to achieving global HIV targets? Longitudinal analyses of nationally representative surveys

Schaefer et al.

Supporting information

Sections in this supporting information document are numbered and referred to with these numbers in the main article.

Table of contents of the supporting information

1. Details on data, measures, and methods 2
2. ART availability in eastern and southern Africa 4
3. Additional data and results on primary sexual behaviour measures 5
4. Data and results on secondary sexual behaviour measures 9
5. Analysis by HIV status 18
6. Sensitivity analysis: Including non-sexually active individuals 22
7. References 30

1. Details on data, measures, and methods

Table S1 provides information on the included surveys and the data available in each. Below details on the measures of sexual behaviour as well as the methods used for the analyses are provided. Further details on data and measures are also provided online (https://dhsprogram.com/).

Recent sexual activity:
The denominator for most analyses in this study was everyone who reported sexual intercourse in the past 12 months. This was based on the question "when was the last time you had sexual intercourse?" that was posed to everyone who reported having ever had sexual intercourse before.

Multiple sexual partnerships:
Multiple partnerships were based on the question "in total, with how many different people have you had sexual intercourse in the last 12 months?" that was asked from DHS phase 4 surveys onwards.

Non-regular and casual sexual partnerships:
From DHS phase 4 onwards, data on the type of sexual partners in the past 12 months were gathered with the question "what is your relationship to the man/woman with whom you last had sex?" Non-regular sexual partners were all types of non-spousal, non-cohabiting partners. Casual partners were those referred to as 'casual acquaintance' or 'commercial sex worker/client'. Casual partners are one type of non-regular partners. In DHS phase 4 surveys, these data were collected for the last two sexual partners in the past 12 months; from DHS phase 5 surveys, these data were collected for the last three sexual partners in the past 12 months. Some phase 5 or later surveys did only collect these data on the last two sexual partners. However, since very few people report three previous sexual partners, these differences have negligible effects.

Age at first sex:
The question on age at first sex was asked everyone reporting ever having had sexual intercourse: "How old were you when you first had sexual intercourse?" Analyses on age at first sex were restricted to everyone who had sex (irrespective of sexual activity in the past 12 months) and those aged 20-29 years.

Condom use:
A question on condom use was asked from DHS phase 3 surveys onwards. In phase 3 surveys, those who reported having ever had sexual intercourse were asked: "the last time you had sex, was a condom used?" From phase 4 onwards, this question was asked for each sexual partner in the past 12 months. In phase 4, the wording was "the last time you had sexual intercourse, was a condom used?" and, referring to another sexual partner in the past 12 months, "have you had sex with any other man in the last 12 months?" From phase 5 onwards, the wording was "the last time you had sexual intercourse (with this second/third person), was a condom used?" Condom use with non-regular or casual partners was defined as condom use the person had last sex with a non-regular or casual partner, which may not be the last sexual partner in the past 12 months.

Details on methods:
To create nationally representative samples, surveys tended to over-sample certain region, which required the application of survey sampling weights to the data so that results are nationally representative. These sampling weights were supplied with each DHS data set. Moreover, individuals were sampled in clusters in each survey. The size and numbers of these clusters differed markedly between countries and within countries over several surveys. Details are provided on the DHS website (https://dhsprogram.com/). Responses of individuals within the same clusters tend to be correlated (non-independence), so this sample design clustering needs to be accounted for. All analyses were conducted in STATA Release 14 (StataCorp. 2015. College Station, TX: StataCorp LP); the svy command was used to apply survey sampling weights and account for sample clustering (see the survey data reference manual for details [1]).

Table S1: Description and data availability for all included surveys.

Country	Survey	(Phase)	Mid-point[a]	Sample size[b] M	Sample size[b] F	HIV status	Age first sex	Multiple partners	Partner type	Condom use last sex	Condom use by partner type
**Ethiopia**	DHS 2000	(4)	Apr 2000	1434	9474		✓	✓	✓	✓	✓
	DHS 2005	(4)	Jun 2005	3197	4199	✓	✓	✓	✓	✓	✓
	DHS 2011	(6)	Mar 2011	7708	10390	✓	✓	✓	✓	✓	✓
	DHS 2016	(7)	Mar 2016	7186	9894		✓	✓	✓	✓	✓
**Kenya**	DHS 1998[c]	(3)	Apr 1998	2579	5560		✓			✓	
	DHS 2003	(4)	Jun 2003	2355	5678	✓	✓	✓	✓	✓	✓
	DHS 2008/09	(5)	Dec 2008	2329	5995	✓	✓	✓	✓	✓	✓
	DHS 2014	(6)	Jul 2014	8698	10680		✓	✓	✓	✓	✓
**Lesotho**[d]	DHS 2004	(4)	Nov 2004	1746	4915	✓	✓	✓	✓	✓[d]	✓[d]
	DHS 2009	(5)	Nov 2009	2236	5524	✓	✓	✓	✓	✓	✓
	DHS 2014	(6)	Oct 2014	2006	4923	✓	✓	✓	✓	✓	✓
**Malawi**	DHS 2000	(4)	Sep 2000	2307	10295		✓	✓	✓	✓	✓
	DHS 2004	(4)	Nov 2004	2418	9159	✓	✓	✓	✓	✓	✓
	DHS 2010	(5)	Aug 2010	4967	16977	✓	✓	✓	✓	✓	✓
	DHS 2015/16	(7)	Dec 2015	5433	18316	✓	✓	✓	✓	✓	✓
**Mozambique**	DHS 1997[c]	(3)	May 1997	1660	5860		✓			✓	
	DHS 2003	(4)	Oct 2003	2106	9744		✓	✓	✓	✓	✓
	AIS 2009	(5)	Jul 2009	3568	4606	✓	✓	✓	✓	✓	✓
	DHS 2011	(6)	Aug 2011	3118	10445		✓	✓	✓	✓	✓
	AIS 2015	(7)	Jul 2015	4067	5589	✓	✓	✓	✓	✓	✓
**Namibia**	DHS 2000	(4)	Oct 2000	2071	4712		✓	✓	✓	✓	✓
	DHS 2006/07	(5)	Jan 2007	2735	6597		✓	✓	✓	✓	✓
	DHS 2013	(6)	Jul 2013	2897	6697	✓	✓	✓	✓	✓	✓
**Rwanda**	DHS 2000	(4)	Jul 2000	1401	5173		✓	✓	✓	✓	✓
	DHS 2005	(4)	Apr 2005	2391	7565	✓	✓	✓	✓	✓	✓
	DHS 2010	(6)	Dec 2010	3181	5851	✓	✓	✓	✓	✓	✓
	DHS 2014/15	(6)	Jan 2015	3296	5173	✓	✓	✓	✓	✓	✓
**Tanzania**[e]	DHS 1996[c]	(3)	Sep 1996	1368	5410		✓			✓	
	DHS 1999	(3)	Oct 1999	2460	3079		✓			✓	
	AIS 2003/4	(5)	Jan 2004	4173	5294	✓	✓	✓	✓	✓	✓
	DHS 2004/05	(4)	Dec 2004	1880	7704		✓	✓	✓	✓	✓
	AIS 2007/08	(5)	Dec 2007	4665	6789	✓	✓	✓	✓	✓	✓
	DHS 2010	(5)	Feb 2010	1750	7585		✓	✓	✓	✓	✓
	AIS 2011/12	(6)	Feb 2012	6060	8237	✓	✓	✓	✓	✓	✓
	DHS 2015/16	(7)	Nov 2015	2629	10216		✓			✓	
**Uganda**	DHS 1995[c]	(3)	May 1995	1428	5145		✓			✓	
	DHS 2000/01	(4)	Dec 2000	1382	5490		✓	✓	✓	✓	✓
	DHS 2006	(5)	Jul 2006	1708	6221		✓	✓	✓	✓	✓
	DHS 2011[f]	(6)	Sep 2011	1575	6174		✓	✓	✓	✓	✓
	DHS 2016	(7)	Aug 2016	3722	13713		✓	✓	✓	✓	✓
**Zambia**	DHS 1996[c]	(3)	Oct 1996	1371	5519		✓			✓	
	DHS 2001/02	(4)	Feb 2002	1558	5643		✓	✓	✓	✓	✓
	DHS 2007	(5)	Jul 2007	4446	5321	✓	✓	✓	✓	✓	✓
	DHS 2013/14	(6)	Dec 2013	10142	12235	✓	✓	✓	✓	✓	✓
**Zimbabwe**	DHS 1994[c]	(3)	Sep 1994	1426	4278		✓			✓	
	DHS 1999	(4)	Oct 1999	1680	4178		✓	✓	✓	✓	✓
	DHS 2005/06	(5)	Nov 2005	4302	5839	✓	✓	✓	✓	✓	✓
	DHS 2010/11	(6)	Dec 2010	4746	6202	✓	✓	✓	✓	✓	✓
	DHS 2015	(7)	Sep 2015	5628	7186	✓	✓	✓	✓	✓	✓

[a] For surveys with even numbers of month of data collection, the mid-point was the earlier of the two middle months.
[b] The sample size refers to the number of males and females aged 15-49 who reported to have had sex in the past 12 months.
[c] DHS phase 3 surveys were used only for secondary analyses on age at first sex (see below).
[d] The Lesotho 2004 DHS only collected condom use information on females.
[e] The Tanzania DHS 2015/16 included only a reduced sexual behaviour section, including age at first sex and condom during last sexual intercourse.
[f] There was a DHS and AIS in Uganda in 2011 but only the DHS data were included to ensure comparability with the preceding and subsequent DHS.

2. ART availability in eastern and southern Africa

Table S2 provides the years when ART was introduced in the eastern and southern African countries included in this analysis and when 30% of adults living with HIV were on treatment. In figures in which these periods are indicated, the first day of each of these years were used as the date of introduction.

Table S2: Availability of ART in the eastern and southern African countries included in the analysis.

**Country**	**Introduction of ART in the public sector**	**30% ART coverage (adults 15+)**
Ethiopia	2005 [2]	2010 [3]
Kenya	2004 [4] [a]	2010 [3]
Lesotho	2005 [5] [b]	2012 [6]
Malawi	2003 [7]	2011 [3]
Mozambique	2004 [8]	2014 [3]
Namibia	2003 [9]	2009 [10] [c]
Rwanda	2003 [11]	2008 [12] [c]
Tanzania	2004 [13]	2012 [3]
Uganda	2004 [14]	2012 [3]
Zambia	2004 [15]	2009 [16] [c]
Zimbabwe	2005 [17] [d]	2011 [18] [c]

The table indicates the year when antiretroviral therapy (ART) was introduced in the public healthcare sector (ART may have been available earlier through private healthcare providers). These years refer to the year when ART was made widely available for free or highly subsidised as opposed to small-scale pilot projects (see notes below). The year of 30% ART coverage refers to the year when 30% of adults (15+ years) who were HIV-positive received ART (eligibility criteria for ART were disregarded).

[a] Pilot provision of ART started in 2001 in 5 sites and expanded to 100 sites at the end of 2004.

[b] ART was first made available at the end of 2004 in one hospital and expanded to 22 sites by the end of 2005.

[c] For calculating ART coverage, the number of adults (15+ years) on treatment was taken from the referenced report and divided by the total estimated number of people living with HIV in the year provided by UNAIDS [3].

[d] Pilot started in 2004 and strongly expanded in 2005

3. Additional data and results on primary sexual behaviour measures

Tables S3-6 provide levels of multiple partnerships, non-regular partnerships, condomless sex with non-regular partners among those with non-regular partners, and condomless sex with non-regular partners among everyone who had sex with 95% CIs by sex for each country and survey, and p-values of logistic regressions comparing consecutive surveys, adjusted for age. Figures 1-4 in the main article present these trends over time.

Table S3: Multiple sexual partnerships, eastern and southern Africa.

			Males				Females			
			Sample	Adj. proportions		Logit	Sample	Adj. proportions		Logit
**Country**	**Survey**	**(Phase)**	**n/N**	**%**	**(95% CI)**	**p-value**	**n/N**	**%**	**(95% CI)**	**p-value**
**Ethiopia**	DHS 2000	(4)	158/1434	10.70	(8.44-13.47)		141/9473	1.58	(1.15-2.15)	
	DHS 2005	(4)	176/3196	4.14	(3.29-5.19)	<0.0001	15/4195	0.24	(0.11-0.50)	<0.0001
	DHS 2011	(6)	562/7706	5.91	(5.09-6.84)	0.0095	73/10384	0.54	(0.35-0.83)	0.0598
	DHS 2016	(7)	443/7185	5.50	(4.61-6.55)	0.5191	47/9884	0.43	(0.28-0.67)	0.5059
**Kenya**	DHS 2003	(4)	380/2355	16.53	(14.81-18.41)		130/5677	2.50	(2.05-3.04)	
	DHS 2008/09	(5)	340/2326	13.08	(11.30-15.08)	0.0198	102/5990	1.67	(1.28-2.17)	0.0208
	DHS 2014	(6)	1381/8694	16.90	(15.82-18.03)	0.0012	182/10680	1.92	(1.54-2.39)	0.3774
**Lesotho**	DHS 2004	(4)	524/1740	30.19	(27.41-33.13)		578/4915	11.06	(10.03-12.17)	
	DHS 2009	(5)	660/2235	29.21	(26.71-31.83)	0.4829	493/5521	8.76	(7.77-9.86)	0.0037
	DHS 2014	(6)	666/2002	34.89	(31.72-38.19)	0.0045	430/4923	8.76	(7.75-9.88)	0.9906
**Malawi**	DHS 2000	(4)	420/2307	18.25	(16.45-20.19)		120/10295	0.99	(0.79-1.24)	
	DHS 2004	(4)	300/2417	11.85	(10.30-13.59)	<0.0001	97/9159	1.08	(0.86-1.34)	0.6287
	DHS 2010	(5)	650/4967	12.54	(11.42-13.74)	0.4983	132/16976	0.88	(0.71-1.09)	0.2865
	DHS 2015/16	(7)	969/5433	16.80	(15.49-18.20)	<0.0001	285/18312	1.65	(1.42-1.92)	<0.0001
**Mozambique**	DHS 2003	(4)	821/2100	35.72	(32.86-38.68)		603/9740	6.22	(5.46-7.08)	
	AIS 2009	(5)	839/3554	22.67	(20.12-25.44)	<0.0001	180/4604	3.59	(2.89-4.45)	<0.0001
	DHS 2011	(6)	1174/3102	32.82	(30.86-34.85)	<0.0001	443/10444	3.61	(3.22-4.05)	0.9775
	AIS 2015	(7)	1028/4056	23.60	(21.50-25.83)	<0.0001	222/5589	3.65	(2.92-4.55)	0.9619
**Namibia**	DHS 2000	(4)	479/2069	21.66	(18.54-25.14)		153/4710	2.87	(2.34-3.52)	
	DHS 2006/07	(5)	414/2733	16.21	(14.31-18.30)	0.0107	144/6594	2.51	(1.97-3.19)	0.3956
	DHS 2013	(6)	386/2897	14.17	(12.52-16.00)	0.1737	213/6696	3.06	(2.56-3.66)	0.1431
**Rwanda**	DHS 2000	(4)	66/1401	4.19	(3.15-5.56)		30/5173	0.50	(0.33-0.77)	
	DHS 2005	(4)	119/2391	5.07	(4.13-6.20)	0.2918	37/5851	0.60	(0.42-0.85)	0.4436
	DHS 2010	(6)	227/3181	7.01	(6.19-7.92)	0.0084	79/7563	1.06	(0.84-1.35)	0.0068
	DHS 2014/15	(6)	252/3293	7.59	(6.68-8.62)	0.3411	99/7978	1.18	(0.94-1.46)	0.5175
**Tanzania**	AIS 2003/04	(5)	1114/4168	27.12	(25.49-28.80)		327/5294	6.11	(5.38-6.94)	
	DHS 2004/05	(4)	542/1880	30.08	(27.26-33.07)	0.0859	296/7704	4.25	(3.68-4.90)	0.0003
	AIS 2007/08	(5)	1061/4664	24.88	(23.35-26.48)	0.0014	196/6789	3.41	(2.92-3.98)	0.0454
	DHS 2010	(5)	472/1748	28.02	(25.37-30.83)	0.0472	291/7585	4.49	(3.83-5.26)	0.0142
	AIS 2011/12	(6)	1583/6059	27.44	(25.75-29.19)	0.7236	365/8236	4.89	(4.28-5.58)	0.4014
**Uganda**	DHS 2000/01	(4)	363/1382	24.60	(21.90-27.53)		136/5490	2.05	(1.64-2.56)	
	DHS 2006	(5)	498/1707	28.69	(26.17-31.36)	0.0400	141/6220	2.40	(1.98-2.91)	0.2440
	DHS 2011	(6)	412/1574	25.76	(23.16-28.54)	0.1277	142/6174	2.20	(1.79-2.72)	0.5437
	DHS 2016	(7)	1063/3722	27.74	(26.00-29.54)	0.2256	430/13713	3.12	(2.76-3.52)	0.0057
**Zambia**	DHS 2001/02	(4)	405/1556	26.81	(24.33-29.44)		146/5643	2.83	(2.36-3.38)	
	DHS 2007	(5)	909/4437	19.78	(18.40-21.23)	<0.0001	95/5321	1.62	(1.29-2.02)	0.0004
	DHS 2013/14	(6)	2101/10138	21.16	(20.03-22.34)	0.1208	263/12234	2.25	(1.90-2.66)	0.0145
**Zimbabwe**	DHS 1999	(4)	343/1680	18.91	(16.87-21.14)		103/4176	2.37	(1.91-2.93)	
	DHS 2005/06	(5)	579/4295	14.13	(12.86-15.50)	0.0002	77/5839	1.33	(1.02-1.73)	0.0008
	DHS 2010/11	(6)	718/4745	15.73	(14.39-17.18)	0.0599	101/6202	1.62	(1.30-2.01)	0.2344
	DHS 2015	(7)	1163/5625	20.66	(19.39-21.99)	<0.0001	127/7182	1.52	(1.22-1.90)	0.9044

Sample sizes (n/N) refer to unadjusted numbers of people reporting multiple sexual partnerships (n) among everyone who sexual intercourse in the past 12 months with data on this variable (N). Proportions (%) and 95% confidence intervals (95% CI) are adjusted for survey design and sampling weights. The p-values refer to results from logistic regressions with odds ratios of multiple sexual partnerships calculated for one survey compared with the preceding one, adjusted for age. These are also adjusted for survey design and sampling weights.

Table S4: Non-regular sexual partnerships, eastern and southern Africa.

			Males				Females			
			Sample	Adj. proportions		Logit	Sample	Adj. proportions		Logit
**Country**	**Survey**	**(Phase)**	**n/N**	**%**	**(95% CI)**	**p-value**	**n/N**	**%**	**(95% CI)**	**p-value**
**Ethiopia**	DHS 2000	(4)	319/1434	19.89	(16.45-23.85)		603/9467	4.54	(3.83-5.38)	
	DHS 2005	(4)	444/3197	8.58	(7.29-10.07)	<0.0001	168/4199	2.80	(2.22-3.53)	0.0008
	DHS 2011	(6)	1105/7708	9.66	(8.60-10.84)	0.2423	538/10390	3.97	(3.38-4.64)	0.0128
	DHS 2016	(7)	1129/7186	11.23	(9.84-12.78)	0.0129	483/9894	3.66	(3.05-4.39)	0.6438
**Kenya**	DHS 2003	(4)	893/2355	39.30	(36.84-41.82)		1002/5678	17.62	(16.42-18.90)	
	DHS 2008/09	(5)	821/2329	35.02	(32.07-38.09)	0.1937	1018/5995	18.10	(16.45-19.88)	0.3711
	DHS 2014	(6)	3289/8698	40.18	(38.40-41.98)	0.0001	1809/10671	19.42	(18.26-20.64)	0.1043
**Lesotho**	DHS 2004	(4)	1121/1746	63.02	(60.22-65.74)		1794/4910	35.63	(33.91-37.39)	
	DHS 2009	(5)	1477/2236	65.06	(62.62-67.43)	0.8231	1861/5524	34.08	(32.46-35.74)	0.1183
	DHS 2014	(6)	1389/2006	70.14	(67.61-72.55)	<0.0001	1736/4923	35.28	(33.42-37.19)	0.2865
**Malawi**	DHS 2000	(4)	828/2306	35.11	(32.79-37.51)		990/10292	8.89	(8.12-9.72)	
	DHS 2004	(4)	632/2417	25.96	(23.80-28.24)	<0.0001	764/9154	8.29	(7.44-9.24)	0.3040
	DHS 2010	(5)	1365/4967	27.02	(25.18-28.95)	0.2886	1470/16977	8.86	(8.21-9.56)	0.0329
	DHS 2015/16	(7)	1968/5433	35.01	(33.20-36.85)	<0.0001	2455/18316	13.19	(12.45-13.96)	<0.0001
**Mozambique**	DHS 2003	(4)	1156/2105	51.40	(47.92-54.87)		2337/9729	21.84	(20.39-23.38)	
	AIS 2009	(5)	1603/3568	39.97	(36.42-43.63)	0.0001	1046/4606	18.54	(16.68-20.55)	0.0161
	DHS 2011	(6)	1770/3118	51.56	(49.17-53.95)	<0.0001	2624/10445	21.35	(20.07-22.70)	0.0321
	AIS 2015	(7)	2006/4067	44.32	(41.83-46.83)	0.0001	1511/5587	22.64	(20.75-24.65)	0.3330
**Namibia**	DHS 2000	(4)	1261/2069	62.32	(57.90-66.54)		2477/4708	54.70	(51.55-57.82)	
	DHS 2006/07	(5)	1621/2731	60.14	(57.52-62.71)	0.8593	3152/6580	49.18	(47.35-51.02)	0.0011
	DHS 2013	(6)	1829/2894	65.89	(63.42-68.28)	<0.0001	3589/6693	56.29	(54.58-57.99)	<0.0001
**Rwanda**	DHS 2000	(4)	223/1400	13.07	(11.25-15.13)		368/5170	6.43	(5.65-7.30)	
	DHS 2005	(4)	401/2391	16.20	(14.47-18.10)	0.0016	504/5851	8.18	(7.42-9.02)	0.0008
	DHS 2010	(6)	581/3181	17.65	(16.17-19.24)	0.8484	740/7565	9.43	(8.68-10.24)	0.0206
	DHS 2014/15	(6)	656/3295	19.15	(17.51-20.89)	0.0127	1035/7976	12.43	(11.64-13.27)	<0.0001
**Tanzania**	AIS 2003/04	(5)	1899/4173	46.13	(44.12-48.16)		1219/5294	23.11	(21.60-24.70)	
	DHS 2004/05	(4)	775/1880	45.00	(41.99-48.04)	0.3276	1328/7704	19.08	(17.68-20.56)	0.0005
	AIS 2007/08	(5)	1677/4665	40.66	(38.71-42.64)	0.3032	1180/6789	20.63	(19.10-22.25)	0.0898
	DHS 2010	(5)	733/1750	44.67	(41.67-47.72)	0.0160	1501/7585	22.76	(21.45-24.12)	0.0263
	AIS 2011/12	(6)	2475/6060	43.36	(41.55-45.18)	0.3559	1626/8237	21.86	(20.56-23.22)	0.4191
**Uganda**	DHS 2000/01	(4)	437/1381	29.03	(25.96-32.29)		855/5483	13.80	(12.34-15.39)	
	DHS 2006	(5)	605/1708	35.67	(33.01-38.42)	<0.0001	979/6221	16.03	(14.85-17.29)	0.0026
	DHS 2011	(6)	531/1575	32.24	(29.18-35.45)	0.0597	966/6174	15.06	(14.03-16.14)	0.1930
	DHS 2016	(7)	1483/3722	41.04	(38.98-43.14)	0.0002	2670/13713	20.06	(19.07-21.09)	<0.0001
**Zambia**	DHS 2001/02	(4)	688/1558	44.95	(41.96-47.97)		991/5643	17.55	(16.20-18.98)	
	DHS 2007	(5)	1742/4446	37.89	(36.01-39.81)	0.0071	997/5321	16.93	(15.62-18.32)	0.5937
	DHS 2013/14	(6)	4015/10139	39.56	(38.20-40.95)	0.0074	2518/12227	19.09	(18.01-20.22)	0.0013
**Zimbabwe**	DHS 1999	(4)	746/1680	42.33	(39.65-45.06)		676/4178	13.84	(12.46-15.34)	
	DHS 2005/06	(5)	1498/4300	34.90	(33.16-36.68)	0.0001	693/5838	11.45	(10.37-12.63)	0.0091
	DHS 2010/11	(6)	1692/4746	33.91	(32.25-35.62)	0.4624	863/6202	11.94	(10.95-13.01)	0.4529
	DHS 2015	(7)	2203/5628	37.25	(35.48-39.06)	<0.0001	1172/7186	14.14	(12.97-15.40)	0.0012

Sample sizes (n/N) refer to unadjusted numbers of people reporting non-regular sexual partnerships (n) among everyone who sexual intercourse in the past 12 months with data on this variable (N). Proportions (%) and 95% confidence intervals (95% CI) are adjusted for survey design and sampling weights. The p-values refer to results from logistic regressions with odds ratios of non-regular sexual partnerships calculated for one survey compared with the preceding one, adjusted for age. These are also adjusted for survey design and sampling weights.

Table S5: Condomless non-regular sexual intercourse among everyone with non-regular partners, eastern and southern Africa.

			Males				Females			
			Sample	Adj. proportions		Logit	Sample	Adj. proportions		Logit
**Country**	**Survey**	**(Phase)**	**n/N**	**%**	**(95% CI)**	**p-value**	**n/N**	**%**	**(95% CI)**	**p-value**
**Ethiopia**	DHS 2000	(4)	197/319	69.35	(60.41-77.04)		455/603	86.38	(81.28-90.26)	
	DHS 2005	(4)	190/442	49.08	(41.43-56.77)	0.0004	117/163	76.43	(64.70-85.16)	0.1389
	DHS 2011	(6)	391/1100	32.66	(28.23-37.41)	0.0004	329/532	71.51	(63.88-78.09)	0.3116
	DHS 2016	(7)	468/1129	49.01	(43.46-54.60)	<0.0001	333/483	79.64	(73.17-84.87)	0.0672
**Kenya**	DHS 2003	(4)	464/892	53.46	(49.57-57.31)		744/1001	76.06	(72.63-79.19)	
	DHS 2008/09	(5)	302/820	37.56	(32.89-42.48)	<0.0001	630/1014	64.77	(60.63-68.69)	<0.0001
	DHS 2014	(6)	896/3288	26.75	(24.62-29.00)	<0.0001	889/1808	44.55	(41.23-47.92)	<0.0001
**Lesotho**	DHS 2004	(4)	NA*				1128/1794	60.68	(57.81-63.48)	
	DHS 2009	(5)	595/1477	37.49	(34.34-40.74)		723/1861	35.52	(33.11-38.00)	<0.0001
	DHS 2014	(6)	356/1389	23.46	(20.76-26.40)	<0.0001	447/1736	23.98	(21.73-26.39)	<0.0001
**Malawi**	DHS 2000	(4)	496/828	60.69	(56.40-64.82)		699/990	71.37	(67.71-74.77)	
	DHS 2004	(4)	342/632	53.40	(48.23-58.49)	0.0302	552/763	69.97	(65.79-73.85)	0.2916
	DHS 2010	(5)	596/1352	44.29	(40.92-47.72)	0.0035	831/1456	54.05	(50.66-57.40)	<0.0001
	DHS 2015/16	(7)	505/1968	26.65	(24.25-29.19)	<0.0001	1182/2455	51.17	(48.33-54.00)	0.2016
**Mozambique**	DHS 2003	(4)	715/1156	66.60	(62.94-70.07)		1694/2335	75.02	(72.12-77.70)	
	AIS 2009	(5)	860/1599	60.84	(56.21-65.28)	0.0450	643/1043	67.44	(63.37-71.27)	0.0006
	DHS 2011	(6)	946/1770	59.53	(56.26-62.71)	0.6381	1580/2624	65.82	(62.80-68.72)	0.5403
	AIS 2015	(7)	980/2005	56.67	(53.03-60.23)	0.2846	847/1511	59.62	(55.90-63.23)	0.0029
**Namibia**	DHS 2000	(4)	408/1259	31.90	(28.19-35.84)		1414/2477	57.23	(53.17-61.21)	
	DHS 2006/07	(5)	342/1621	21.69	(19.16-24.44)	<0.0001	1286/3149	37.79	(35.43-40.22)	<0.0001
	DHS 2013	(6)	405/1829	20.17	(18.00-22.53)	0.2872	1340/3589	34.49	(32.45-36.59)	0.0257
**Rwanda**	DHS 2000	(4)	90/223	48.70	(40.36-57.12)		296/368	85.13	(79.08-89.65)	
	DHS 2005	(4)	247/396	65.15	(59.54-70.37)	0.0062	398/499	80.35	(75.98-84.10)	0.1720
	DHS 2010	(6)	207/581	36.86	(32.83-41.07)	<0.0001	458/736	63.75	(59.97-67.37)	<0.0001
	DHS 2014/15	(6)	217/656	33.95	(30.12-37.99)	0.2945	531/1035	52.45	(49.13-55.76)	<0.0001
**Tanzania**	AIS 2003/04	(5)	972/1892	50.13	(47.02-53.25)		770/1216	61.91	(58.01-65.66)	
	DHS 2004/05	(4)	391/775	48.88	(44.08-53.72)	0.5943	907/1327	67.19	(63.58-70.61)	0.0770
	AIS 2007/08	(5)	815/1676	46.68	(43.49-49.89)	0.5544	701/1178	57.27	(53.87-60.60)	<0.0001
	DHS 2010	(5)	335/731	42.75	(38.14-47.5)	0.2059	890/1500	58.27	(55.10-61.36)	0.6254
	AIS 2011/12	(6)	1031/2469	39.85	(37.09-42.68)	0.2527	815/1624	49.60	(46.30-52.91)	0.0002
**Uganda**	DHS 2000/01	(4)	158/437	39.55	(34.45-44.90)		485/854	61.72	(57.00-66.23)	
	DHS 2006	(5)	252/605	41.67	(37.47-45.99)	0.5551	628/973	65.15	(61.54-68.59)	0.2901
	DHS 2011	(6)	202/531	38.39	(33.32-43.72)	0.3177	514/966	54.52	(50.57-58.42)	0.0001
	DHS 2016	(7)	644/1483	42.72	(39.73-45.77)	0.1638	1674/2670	62.69	(60.45-64.87)	0.0005
**Zambia**	DHS 2001/02	(4)	392/688	55.67	(51.18-60.07)		662/988	66.92	(63.32-70.34)	
	DHS 2007	(5)	862/1740	50.03	(47.15-52.91)	0.0405	612/995	62.57	(58.84-66.16)	0.0829
	DHS 2013/14	(6)	1844/4015	45.34	(43.32-47.38)	0.0083	1453/2518	58.77	(56.24-61.26)	0.0963
**Zimbabwe**	DHS 1999	(4)	248/746	29.50	(25.54-33.80)		427/671	57.50	(52.96-61.91)	
	DHS 2005/06	(5)	444/1497	28.51	(24.74-32.60)	0.6348	386/693	54.35	(49.36-59.25)	0.3553
	DHS 2010/11	(6)	431/1692	23.38	(21.15-25.77)	0.0503	389/863	42.35	(38.61-46.19)	0.0003
	DHS 2015	(7)	392/2203	17.82	(15.95-19.84)	0.0005	413/1172	35.27	(32.07-38.60)	0.0102

Sample sizes (n/N) refer to unadjusted numbers of people reporting not using condoms during the non-regular casual sexual intercourse (n) among everyone who reported non-regular sexual intercourse in the past 12 months with data on this variable (N). Proportions (%) and 95% confidence intervals (95% CI) are adjusted for survey design and sampling weights. The p-values refer to results from logistic regressions with odds ratios of not using condoms during last non-regular sexual intercourse calculated for one survey compared with the preceding one, adjusted for age. These are also adjusted for survey design and sampling weights.

* The Lesotho 2004 DHS only collected condom use information on females.

Table S6: Condomless non-regular sexual intercourse among everyone who had a sexual partner in the past 12 months, eastern and southern Africa.

			**Males**				**Females**			
			**Sample**	**Adj. proportions**		**Logit**	**Sample**	**Adj. proportions**		**Logit**
**Country**	**Survey**	**(Phase)**	**n/N**	**%**	**(95% CI)**	**p-value**	**n/N**	**%**	**(95% CI)**	**p-value**
**Ethiopia**	DHS 2000	(4)	197/1434	13.79	(10.84-17.40)		455/9467	3.92	(3.26-4.72)	
	DHS 2005	(4)	190/3197	4.18	(3.26-5.34)	<0.0001	117/4199	2.08	(1.53-2.82)	0.0004
	DHS 2011	(6)	391/7708	3.14	(2.63-3.75)	0.0523	329/10390	2.80	(2.33-3.37)	0.0921
	DHS 2016	(7)	468/7186	5.50	(4.53-6.67)	<0.0001	333/9894	2.92	(2.37-3.59)	0.6844
**Kenya**	DHS 2003	(4)	464/2355	20.99	(19.05-23.07)		744/5678	13.39	(12.38-14.47)	
	DHS 2008/09	(5)	302/2329	13.15	(11.25-15.30)	<0.0001	630/5995	11.70	(10.53-12.97)	0.0875
	DHS 2014	(6)	896/8698	10.74	(9.82-11.75)	0.0314	889/10671	8.65	(7.93-9.42)	<0.0001
**Lesotho**	DHS 2004	(4)					1128/4910	21.62	(20.32-22.98)	
	DHS 2009	(5)	595/2236	24.39	(22.19-26.73)		723/5524	12.10	(11.22-13.05)	<0.0001
	DHS 2014	(6)	356/2006	16.46	(14.49-18.62)	<0.0001	447/4923	8.46	(7.58-9.44)	<0.0001
**Malawi**	DHS 2000	(4)	496/2306	21.31	(19.33-23.43)		699/10292	6.34	(5.74-7.00)	
	DHS 2004	(4)	342/2417	13.86	(12.36-15.51)	<0.0001	552/9154	5.80	(5.22-6.44)	0.1891
	DHS 2010	(5)	596/4967	11.87	(10.78-13.06)	0.0127	831/16977	4.74	(4.30-5.22)	0.0689
	DHS 2015/16	(7)	505/5433	9.33	(8.43-10.31)	<0.0001	1182/18316	6.75	(6.21-7.32)	<0.0001
**Mozambique**	DHS 2003	(4)	715/2105	34.23	(31.32-37.27)		1694/9729	16.37	(15.23-17.59)	
	AIS 2009	(5)	860/3568	24.24	(21.64-27.05)	<0.0001	643/4606	12.44	(11.07-13.95)	0.0001
	DHS 2011	(6)	946/3118	30.69	(28.69-32.77)	0.0039	1580/10445	14.05	(13.04-15.13)	0.1002
	AIS 2015	(7)	980/4067	25.09	(22.96-27.34)	0.0007	847/5587	13.50	(12.20-14.91)	0.4795
**Namibia**	DHS 2000	(4)	408/2069	19.84	(17.36-22.56)		1414/4708	31.31	(28.76-33.98)	
	DHS 2006/07	(5)	342/2731	13.04	(11.50-14.75)	<0.0001	1286/6580	18.57	(17.44-19.76)	<0.0001
	DHS 2013	(6)	405/2894	13.29	(11.88-14.84)	0.6870	1340/6693	19.42	(18.24-20.65)	0.1845
**Rwanda**	DHS 2000	(4)	90/1400	6.37	(4.98-8.10)		296/5170	5.47	(4.80-6.22)	
	DHS 2005	(4)	247/2391	10.41	(8.92-12.11)	0.0001	398/5851	6.51	(5.81-7.28)	0.0257
	DHS 2010	(6)	207/3181	6.51	(5.65-7.48)	<0.0001	458/7565	5.98	(5.46-6.55)	0.2722
	DHS 2014/15	(6)	217/3295	6.50	(5.66-7.46)	0.6577	531/7976	6.52	(5.97-7.11)	0.1370
**Tanzania**	AIS 2003/04	(5)	972/4173	23.06	(21.26-24.97)		770/5294	14.28	(13.15-15.49)	
	DHS 2004/05	(4)	391/1880	22.00	(19.44-24.79)	0.3467	907/7704	12.81	(11.81-13.87)	0.0936
	AIS 2007/08	(5)	815/4665	18.97	(17.33-20.72)	0.3054	701/6789	11.80	(10.84-12.83)	0.2109
	DHS 2010	(5)	335/1750	19.09	(16.73-21.69)	0.9772	890/7585	13.26	(12.40-14.18)	0.0240
	AIS 2011/12	(6)	1031/6060	17.24	(15.89-18.68)	0.1367	815/8237	10.84	(9.96-11.78)	0.0003
**Uganda**	DHS 2000/01	(4)	158/1381	11.48	(9.70-13.54)		485/5483	8.51	(7.50-9.64)	
	DHS 2006	(5)	252/1708	14.86	(13.01-16.93)	0.0044	628/6221	10.40	(9.57-11.30)	0.0017
	DHS 2011	(6)	202/1575	12.37	(10.47-14.56)	0.0685	514/6174	8.21	(7.43-9.07)	0.0003
	DHS 2016	(7)	644/3722	17.53	(16.05-19.12)	0.0021	1674/13713	12.57	(11.88-13.30)	<0.0001
**Zambia**	DHS 2001/02	(4)	392/1558	25.02	(22.55-27.67)		662/5643	11.73	(10.74-12.80)	
	DHS 2007	(5)	862/4446	18.94	(17.55-20.41)	0.0013	612/5321	10.57	(9.68-11.53)	0.5089
	DHS 2013/14	(6)	1844/10139	17.94	(17.03-18.89)	0.3224	1453/12227	11.22	(10.48-12.00)	0.0899
**Zimbabwe**	DHS 1999	(4)	248/1680	12.49	(10.78-14.43)		427/4178	7.86	(6.97-8.85)	
	DHS 2005/06	(5)	444/4300	9.94	(8.53-11.56)	0.0995	386/5838	6.22	(5.47-7.08)	0.0086
	DHS 2010/11	(6)	431/4746	7.93	(7.09-8.85)	0.0855	389/6202	5.06	(4.50-5.68)	0.0291
	DHS 2015	(7)	392/5628	6.64	(5.88-7.49)	0.0549	413/7186	4.99	(4.40-5.66)	0.6293

Sample sizes (n/N) refer to unadjusted numbers of people reporting not using condoms during the non-regular casual sexual intercourse (n) among everyone who reported any sexual partner in the past 12 months with (N). Proportions (%) and 95% confidence intervals (95% CI) are adjusted for survey design and sampling weights. The p-values refer to results from logistic regressions with odds ratios of not using condoms during last non-regular sexual intercourse calculated for one survey compared with the preceding one, adjusted for age. These are also adjusted for survey design and sampling weights.

* The Lesotho 2004 DHS only collected condom use information on females.

4. Data and results on secondary sexual behaviour measures

Tables S7-9 provide levels of casual partnerships, condomless sex casual partners, and age at first sex below 18 years with 95% CIs by sex for each country and survey, and p-values of logistic regressions comparing consecutive surveys, adjusted for age. Figures S1-3 present these trends over time.

Table S7: Casual sexual partnerships, eastern and southern Africa.

			Males				Females			
			Sample	Adj. proportions		Logit	Sample	Adj. proportions		Logit
**Country**	**Survey**	**(Phase)**	**n/N**	**%**	**(95% CI)**	**p-value**	**n/N**	**%**	**(95% CI)**	**p-value**
**Ethiopia**	DHS 2000	(4)	33/1312	3.82	(2.50-5.81)		88/9393	1.14	(0.79-1.65)	
	DHS 2005	(4)	141/3194	2.65	(2.01-3.48)	0.0513	28/4193	0.32	(0.18-0.55)	0.0002
	DHS 2011	(6)	345/7700	3.10	(2.59-3.71)	0.3448	83/10374	0.64	(0.42-0.97)	0.0486
	DHS 2016	(7)	356/7182	3.26	(2.62-4.06)	0.4962	55/9888	0.55	(0.36-0.84)	0.6829
**Kenya**	DHS 2003	(4)	232/2218	10.81	(9.21-12.64)		54/5578	0.88	(0.64-1.20)	
	DHS 2008/09	(5)	152/2327	6.57	(5.25-8.19)	0.0004	65/5964	1.28	(0.85-1.92)	0.1574
	DHS 2014	(6)	741/8664	9.68	(8.75-10.69)	0.0012	147/10643	1.38	(1.10-1.72)	0.7458
**Lesotho**	DHS 2004	(4)	71/1701	4.58	(3.45-6.05)		43/4829	0.74	(0.50-1.11)	
	DHS 2009	(5)	256/2236	11.28	(9.86-12.88)	<0.0001	139/5513	2.86	(2.33-3.50)	<0.0001
	DHS 2014	(6)	274/2005	14.09	(12.07-16.38)	0.0347	175/4918	3.49	(2.92-4.17)	0.1569
**Malawi**	DHS 2000	(4)	188/2238	8.84	(7.45-10.45)		46/10267	0.41	(0.28-0.59)	
	DHS 2004	(4)	147/2358	6.48	(5.35-7.82)	0.0367	47/9133	0.44	(0.31-0.64)	0.7813
	DHS 2010	(5)	211/4951	4.67	(3.94-5.53)	0.0067	43/16925	0.29	(0.18-0.45)	0.1213
	DHS 2015/16	(7)	349/5428	6.38	(5.63-7.23)	0.0079	118/18310	0.62	(0.47-0.80)	0.0044
**Mozambique**	DHS 2003	(4)	442/1882	23.97	(21.12-27.06)		1007/9722	10.26	(9.30-11.3)	
	AIS 2009	(5)	476/3520	11.65	(10.04-13.48)	<0.0001	144/4542	2.79	(2.27-3.43)	<0.0001
	DHS 2011	(6)	960/3114	25.20	(23.50-26.98)	<0.0001	605/10397	5.39	(4.87-5.97)	<0.0001
	AIS 2015	(7)	759/4065	16.33	(14.32-18.57)	<0.0001	334/5582	5.28	(4.41-6.31)	0.8416
**Namibia**	DHS 2000	(4)	342/1878	14.51	(11.06-18.81)		60/4668	1.41	(0.97-2.04)	
	DHS 2006/07	(5)	512/2727	18.97	(16.70-21.47)	0.0478	76/6570	1.23	(0.93-1.64)	0.5760
	DHS 2013	(6)	438/2889	14.49	(12.84-16.32)	0.0049	93/6683	1.16	(0.89-1.51)	0.7742
**Rwanda**	DHS 2000	(4)	98/1338	6.06	(4.78-7.65)		68/5027	1.24	(0.94-1.62)	
	DHS 2005	(4)	91/2323	3.69	(3.00-4.53)	0.0024	40/5811	0.63	(0.46-0.88)	0.0035
	DHS 2010	(6)	141/3174	4.24	(3.58-5.02)	0.7127	53/7517	0.74	(0.57-0.97)	0.4425
	DHS 2014/15	(6)	121/3295	3.52	(2.85-4.34)	0.2690	62/7942	0.69	(0.52-0.94)	0.7588
**Tanzania**	AIS 2003/04	(5)	892/4161	20.54	(19.09-22.07)		180/5284	3.34	(2.86-3.91)	
	DHS 2004/05	(4)	413/1879	24.40	(22.13-26.83)	0.0069	150/7688	2.34	(1.93-2.83)	0.0070
	AIS 2007/08	(5)	525/4662	13.37	(12.19-14.64)	<0.0001	117/6782	2.02	(1.65-2.47)	0.3574
	DHS 2010	(5)	173/1744	11.51	(9.75-13.53)	0.0921	81/7567	1.14	(0.87-1.49)	0.0009
	AIS 2011/12	(6)	603/6052	10.62	(9.63-11.69)	0.3334	129/8229	1.52	(1.23-1.87)	0.0876
**Uganda**	DHS 2000/01	(4)	67/1307	4.29	(3.17-5.79)		33/5400	0.54	(0.34-0.86)	
	DHS 2006	(5)	155/1702	9.49	(7.61-11.77)	<0.0001	78/6189	1.48	(1.15-1.91)	0.0001
	DHS 2011	(6)	110/1571	7.00	(5.53-8.83)	0.0572	102/6163	1.80	(1.41-2.30)	0.2755
	DHS 2016	(7)	413/3722	11.30	(10.12-12.59)	0.0006	250/13696	1.90	(1.64-2.20)	0.7304
**Zambia**	DHS 2001/02	(4)	195/1464	14.09	(12.11-16.34)		42/5595	0.76	(0.56-1.04)	
	DHS 2007	(5)	342/4439	7.56	(6.72-8.50)	<0.0001	54/5295	0.91	(0.66-1.25)	0.3081
	DHS 2013/14	(6)	498/10127	5.17	(4.50-5.93)	<0.0001	66/12198	0.55	(0.41-0.75)	0.0327
**Zimbabwe**	DHS 1999	(4)	79/1567	6.22	(4.86-7.93)		56/4106	1.53	(1.08-2.16)	
	DHS 2005/06	(5)	200/4289	4.77	(4.05-5.61)	0.0893	28/5824	0.45	(0.30-0.69)	<0.0001
	DHS 2010/11	(6)	241/4742	5.17	(4.44-6.00)	0.3053	45/6188	0.58	(0.41-0.81)	0.3848
	DHS 2015	(7)	391/5626	6.64	(5.86-7.52)	0.0045	67/7182	0.74	(0.51-1.07)	0.3503

Sample sizes (n/N) refer to unadjusted numbers of people reporting casual sexual partnerships (n) among everyone who sexual intercourse in the past 12 months with data on this variable (N). Proportions (%) and 95% confidence intervals (95% CI) are adjusted for survey design and sampling weights. The p-values refer to results from logistic regressions with odds ratios of casual sexual partnerships calculated for one survey compared with the preceding one, adjusted for age. These are also adjusted for survey design and sampling weights.

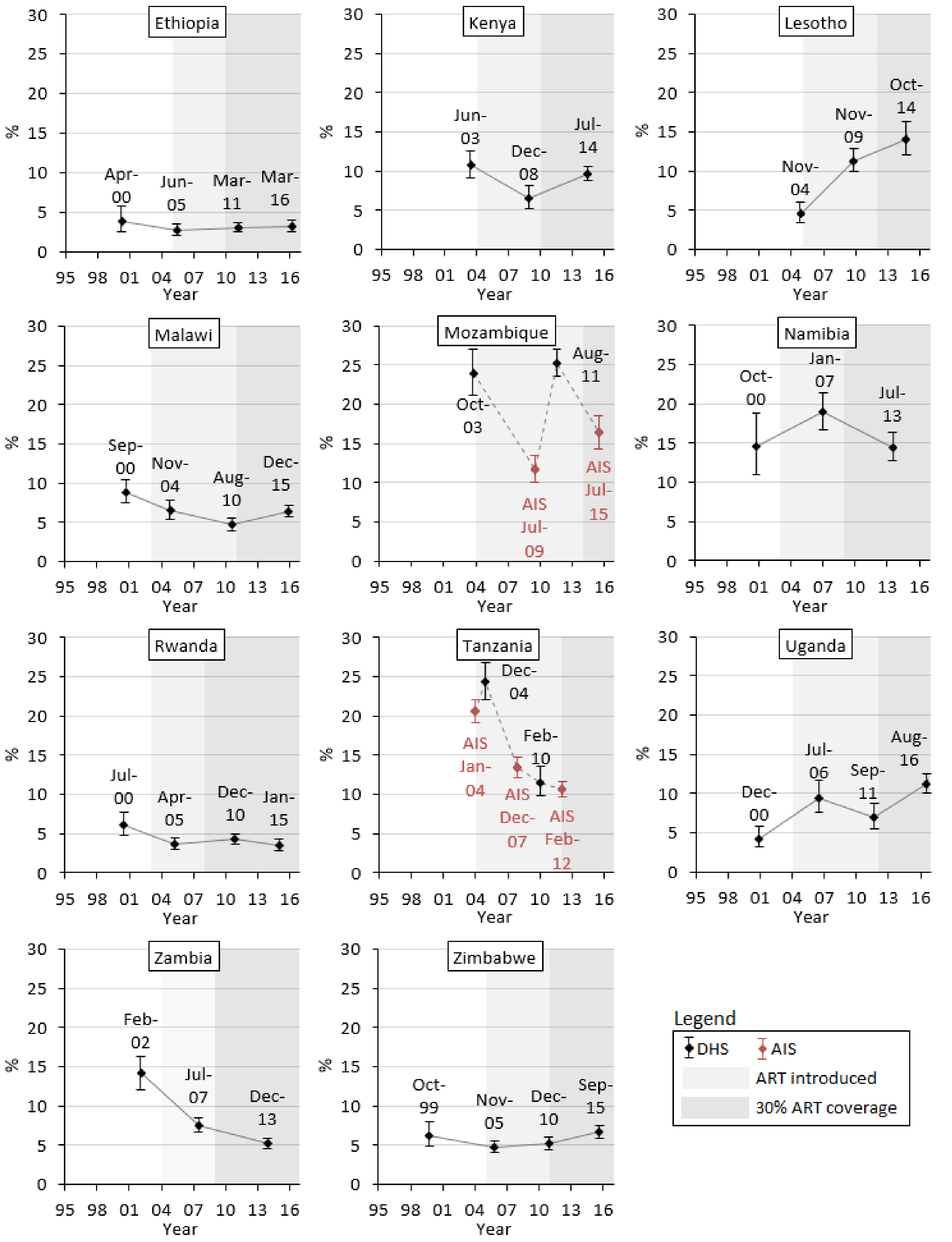

Figure S1a. Trends in casual sexual partnerships among males (15-49 years), eastern and southern Africa. Casual sexual partnerships were defined as reporting at least one casual sexual partner in the past 12. Dates refer to the mid-points of the survey data collection period. Data from AIS are indicated in red. Data from different survey types are linked with dashed lines. Shaded areas indicate the years in which ART was introduced into the public healthcare sector in each country and from when 30% of adult PLWH (15+ years) were in treatment (disregarding treatment eligibility criteria; see Supporting Information, Section 2).

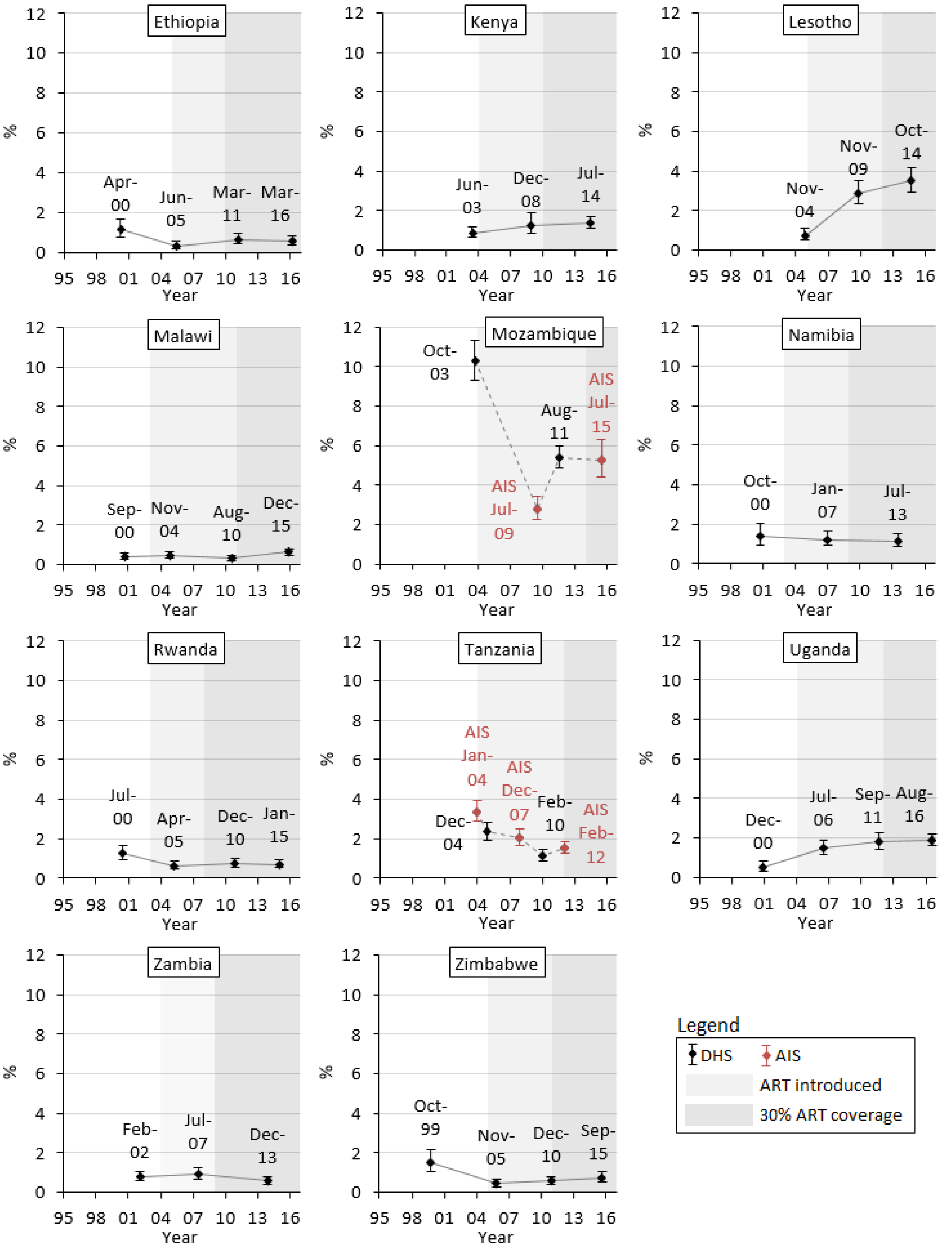

Figure S1b. Trends in casual sexual partnerships among females (15-49 years), eastern and southern Africa. Casual sexual partnerships were defined as reporting at least one casual sexual partner in the past 12. Dates refer to the mid-points of the survey data collection period. Data from AIS are indicated in red. Data from different survey types are linked with dashed lines. Shaded areas indicate the years in which ART was introduced into the public healthcare sector in each country and from when 30% of adult PLWH (15+ years) were in treatment (disregarding treatment eligibility criteria; see Supporting Information, Section 2).

Table S8: Condomless casual sexual intercourse among everyone who had a casual sexual partner in the past 12 months, eastern and southern Africa.

			Males				Females			
			Sample	Adj. proportions		Logit	Sample	Adj. proportions		Logit
**Country**	**Survey**	**(Phase)**	**n/N**	**%**	**(95% CI)**	**p-value**	**n/N**	**%**	**(95% CI)**	**p-value**
**Ethiopia**	DHS 2000	(4)	15/30	42.92	(23.10-65.30)		55/83	82.86	(65.57-92.47)	
	DHS 2005	(4)	56/141	51.51	(38.04-64.77)	0.2640	10/28	42.74	(19.34-69.91)	0.0266
	DHS 2011	(6)	85/345	18.01	(12.24-25.69)	<0.0001	21/82	39.59	(22.35-59.87)	0.9319
	DHS 2016	(7)	109/355	34.16	(26.04-43.33)	0.0047	29/54	68.74	(48.69-83.60)	0.0721
**Kenya**	DHS 2003	(4)	92/219	42.91	(35.20-50.98)		36/53	65.72	(48.18-79.81)	
	DHS 2008/09	(5)	44/152	27.68	(19.99-36.96)	0.0190	35/65	60.11	(46.11-72.64)	0.6374
	DHS 2014	(6)	166/738	20.81	(17.32-24.80)	0.1943	55/146	30.98	(22.56-40.88)	0.0014
**Lesotho**	DHS 2004	(4)	NA*				23/42	55.74	(34.56-75.02)	
	DHS 2009	(5)	108/256	38.09	(31.56-45.09)	<0.0001	73/136	46.41	(35.93-57.21)	0.4310
	DHS 2014	(6)	69/274	17.98	(13.36-23.76)	<0.0001	56/175	26.67	(20.16-34.38)	0.0014
**Malawi**	DHS 2000	(4)	95/178	54.70	(44.86-64.18)		30/45	71.21	(55.09-83.30)	
	DHS 2004	(4)	71/141	54.52	(44.89-63.82)	0.9719	35/47	76.37	(59.68-87.59)	0.6806
	DHS 2010	(5)	77/210	37.70	(29.34-46.86)	0.0104	24/43	50.93	(28.95-72.56)	0.0876
	DHS 2015/16	(7)	80/349	23.28	(18.37-29.03)	0.0081	58/118	53.73	(40.05-66.88)	0.9063
**Mozambique**	DHS 2003	(4)	260/375	76.22	(71.11-80.68)		787/1002	80.98	(77.39-84.12)	
	AIS 2009	(5)	224/469	57.18	(49.71-64.34)	<0.0001	95/144	72.11	(62.55-80.01)	0.0681
	DHS 2011	(6)	483/959	54.98	(51.19-58.72)	0.5285	386/604	70.91	(66.62-74.86)	0.6079
	AIS 2015	(7)	321/758	50.62	(43.85-57.36)	0.3096	198/334	62.51	(54.06-70.27)	0.0710
**Namibia**	DHS 2000	(4)	45/332	16.18	(11.61-22.10)		17/60	35.17	(20.98-52.57)	
	DHS 2006/07	(5)	92/512	16.58	(12.88-21.08)	0.8933	23/76	30.96	(18.47-47.03)	0.9607
	DHS 2013	(6)	66/438	14.02	(10.67-18.20)	0.4328	25/91	19.50	(12.29-29.51)	0.0566
**Rwanda**	DHS 2000	(4)	42/94	55.47	(42.77-67.48)		58/66	90.20	(76.17-96.36)	
	DHS 2005	(4)	49/91	55.84	(45.63-65.58)	0.9826	34/40	86.46	(71.92-94.09)	0.5565
	DHS 2010	(6)	37/140	27.74	(20.63-36.17)	<0.0001	35/53	67.81	(54.73-78.59)	0.0424
	DHS 2014/15	(6)	43/121	35.84	(27.89-44.67)	0.1585	31/62	52.79	(39.94-65.27)	0.0864
**Tanzania**	AIS 2003/04	(5)	430/889	46.05	(42.17-49.97)		109/178	62.66	(54.66-70.02)	
	DHS 2004/05	(4)	192/413	41.53	(35.45-47.88)	0.1489	93/150	61.37	(51.10-70.73)	0.8537
	AIS 2007/08	(5)	262/525	49.28	(43.85-54.73)	0.0229	66/117	54.93	(44.31-65.12)	0.3680
	DHS 2010	(5)	64/172	39.30	(30.43-48.94)	0.0536	50/81	56.72	(44.69-68.00)	0.8119
	AIS 2011/12	(6)	243/603	39.52	(33.99-45.32)	0.9197	62/129	51.56	(41.59-61.41)	0.5364
**Uganda**	DHS 2000/01	(4)	18/59	33.75	(22.76-46.83)		19/31	67.84	(45.43-84.24)	
	DHS 2006	(5)	57/154	36.35	(29.30-44.05)	0.7149	46/78	56.66	(44.96-67.66)	0.3933
	DHS 2011	(6)	42/110	41.03	(30.89-52.00)	0.4761	53/101	63.69	(50.77-74.89)	0.3531
	DHS 2016	(7)	174/413	40.92	(35.50-46.58)	0.9798	158/250	63.52	(56.88-69.68)	0.9902
**Zambia**	DHS 2001/02	(4)	99/183	53.12	(44.70-61.37)		27/42	62.50	(46.68-76.05)	
	DHS 2007	(5)	146/342	42.18	(36.10-48.50)	0.0419	33/53	61.06	(43.95-75.82)	0.9224
	DHS 2013/14	(6)	235/498	44.47	(38.64-50.45)	0.6867	33/66	48.20	(33.87-62.83)	0.1368
**Zimbabwe**	DHS 1999	(4)	13/72	13.57	(7.77-22.62)		15/53	28.49	(16.89-43.84)	
	DHS 2005/06	(5)	54/200	22.70	(16.78-29.96)	0.1005	14/28	48.59	(29.30-68.30)	0.1189
	DHS 2010/11	(6)	39/240	13.23	(9.23-18.60)	0.0233	20/45	37.50	(23.36-54.15)	0.3962
	DHS 2015	(7)	46/390	11.52	(8.67-15.15)	0.5773	15/67	24.26	(14.16-38.34)	0.1878

Sample sizes (n/N) refer to unadjusted numbers of people reporting condom use during the last casual sexual intercourse (n) among everyone who reported casual sexual intercourse in the past 12 months with data on this variable (N). Proportions (%) and 95% confidence intervals (95% CI) are adjusted for survey design and sampling weights. The p-values refer to the results from Pearson's chi-squared (χ^2) tests comparing the values for one survey with the previous one. These are also adjusted for survey design and sampling weights.

* The Lesotho 2004 DHS only collected condom use information on females.

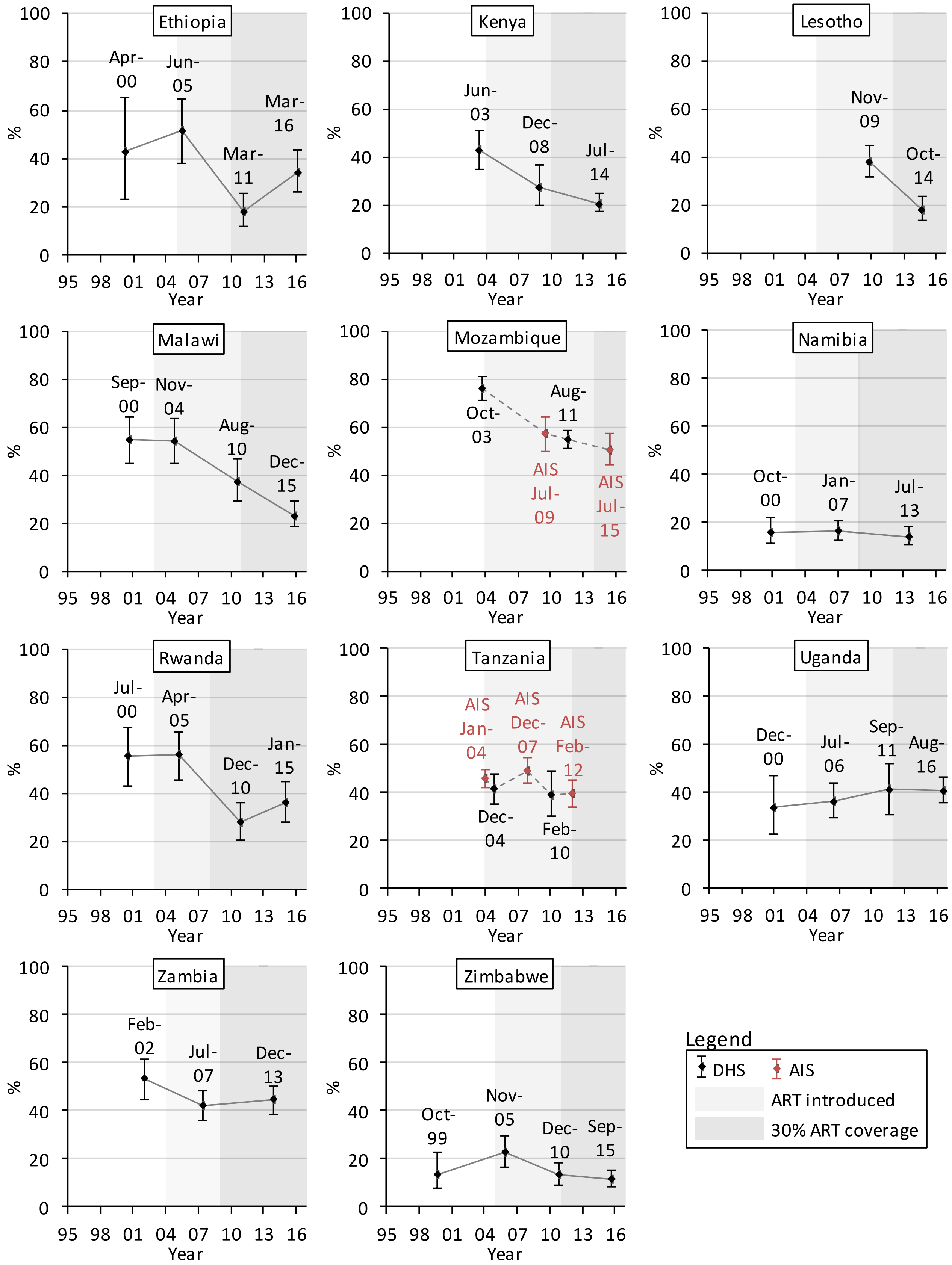

Figure S2a. Trends in condomless sex with the last casual sexual partner among males (15-49 years), eastern and southern Africa. The condom use refers to the last sexual intercourse in the past 12 months with a casual partner among everyone who had such a casual in the past 12 months. Dates refer to the mid-points of the survey data collection period. Data from AIS are indicated in red. Data from different survey types are linked with dashed lines. Shaded areas indicate the years in which ART was introduced into the public healthcare sector in each country and from when 30% of adult PLWH (15+ years) were in treatment (disregarding treatment eligibility criteria; see Supporting Information, Section 2).

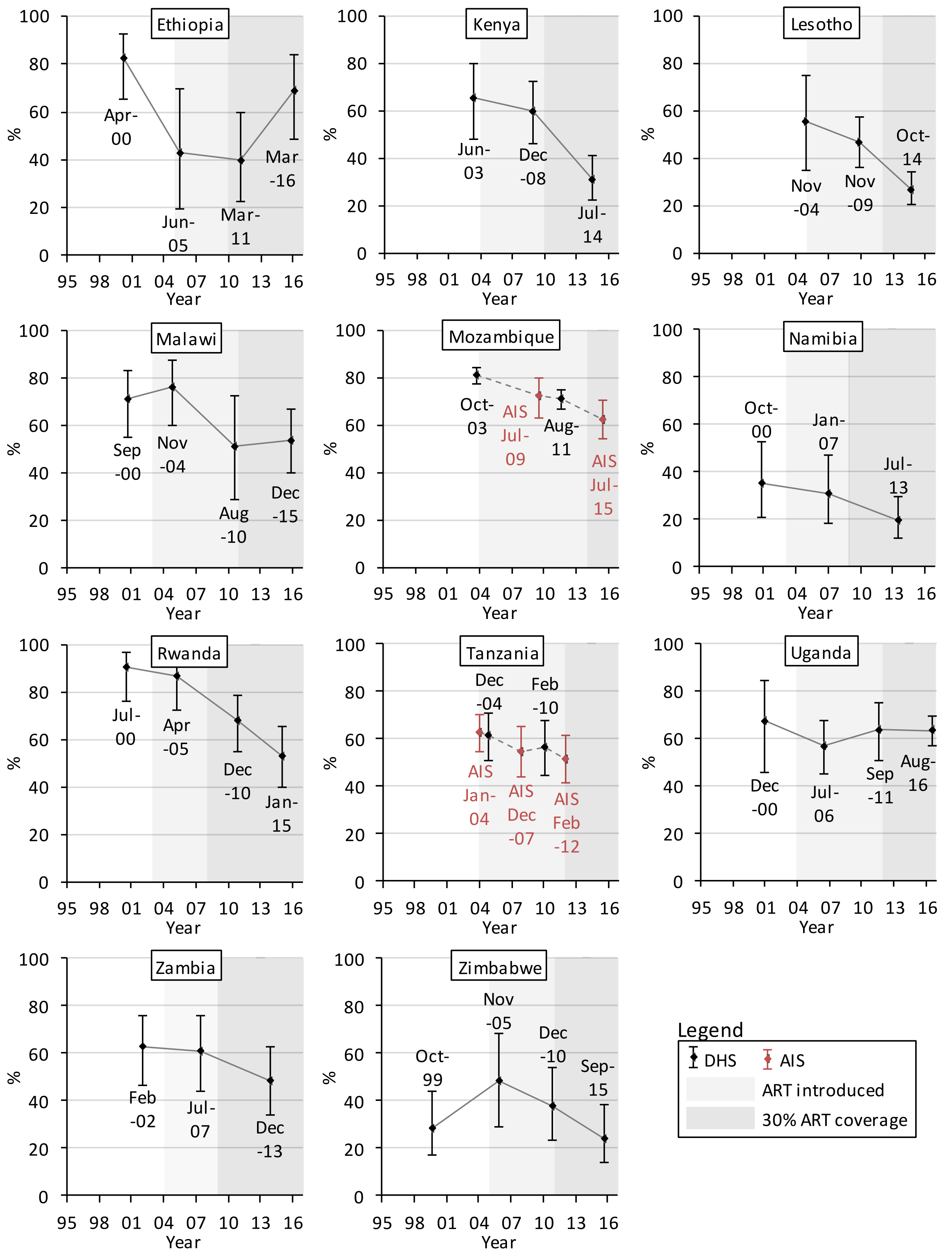

Figure S2b. Trends in condomless sex with the last casual sexual partner among females (15-49 years), eastern and southern Africa. The condom use refers to the last sexual intercourse in the past 12 months with a casual partner among everyone who had such a casual in the past 12 months. Dates refer to the mid-points of the survey data collection period. Data from AIS are indicated in red. Data from different survey types are linked with dashed lines. Shaded areas indicate the years in which ART was introduced into the public healthcare sector in each country and from when 30% of adult PLWH (15+ years) were in treatment (disregarding treatment eligibility criteria; see Supporting Information, Section 2).

Table S9: Age at first sex before the age of 18 years, eastern and southern Africa.

			Males				Females			
			Sample	Adj. proportions		Logit	Sample	Adj. proportions		Logit
**Country**	**Survey**	**(Phase)**	**n/N**	**%**	**(95% CI)**	**p-value**	**n/N**	**%**	**(95% CI)**	**p-value**
**Ethiopia**	DHS 2000	(4)	192/560	32.65	(27.18-38.65)		2802/4547	63.60	(60.98-66.13)	
	DHS 2005	(4)	351/1203	25.28	(22.09-28.77)	0.0155	2626/4127	66.79	(64.32-69.18)	0.0807
	DHS 2011	(6)	833/3045	24.53	(21.95-27.32)	0.9626	2946/5150	59.43	(57.05-61.78)	<0.0001
	DHS 2016	(7)	656/2717	21.70	(19.23-24.40)	0.1734	2690/4762	57.94	(55.16-60.66)	0.4181
**Kenya**	DHS 1998	(3)	735/992	73.30	(69.75-76.56)		1651/2676	63.43	(61.06-65.74)	
	DHS 2003	(4)	657/1062	64.44	(61.22-67.52)	0.0002	1462/2775	54.64	(52.02-57.23)	<0.0001
	DHS 2008/09	(5)	635/1006	60.55	(55.50-65.39)	0.1824	1519/2898	50.92	(47.49-54.34)	0.0882
	DHS 2014	(6)	2233/3572	63.39	(61.19-65.54)	0.2217	5898/10622	51.18	(49.36-52.99)	0.8040
**Lesotho**	DHS 2004	(4)	392/789	49.45	(45.41-53.51)		963/2297	40.78	(38.45-43.16)	
	DHS 2009	(5)	619/1028	60.21	(56.65-63.67)	0.0002	1230/2616	46.06	(43.70-48.45)	0.0015
	DHS 2014	(6)	547/872	62.79	(58.80-66.61)	0.3173	1025/2277	43.50	(40.59-46.45)	0.1771
**Malawi**	DHS 2000	(4)	598/1081	52.41	(48.75-56.05)		3421/5209	64.30	(62.37-66.20)	
	DHS 2004	(4)	550/1127	46.90	(43.73-50.10)	0.0551	2958/4820	58.38	(56.50-60.24)	<0.0001
	DHS 2010	(5)	1057/2091	51.25	(48.34-54.14)	0.0857	5306/8400	61.85	(60.22-63.45)	0.0020
	DHS 2015/16	(7)	1142/2246	49.89	(47.21-52.57)	0.2791	5369/8718	61.15	(59.75-62.53)	0.4096
**Mozambique**	DHS 1997	(3)	366/604	52.39	(45.86-58.83)		2432/3198	80.25	(77.73-82.55)	
	DHS 2003	(4)	532/795	66.28	(62.01-70.31)	0.0005	3556/4609	78.81	(77.24-80.30)	0.3399
	AIS 2009	(5)	852/1359	62.28	(58.03-66.36)	0.2043	1508/2072	74.18	(71.27-76.90)	0.0027
	DHS 2011	(6)	815/1181	68.37	(64.89-71.66)	0.0338	3760/4775	79.10	(77.48-80.64)	0.0020
	AIS 2015	(7)	1066/1602	67.67	(64.55-70.64)	0.7243	1786/2454	74.87	(72.51-77.09)	0.0020
**Namibia**	DHS 2000	(4)	608/954	64.31	(60.08-68.34)		1044/2263	44.48	(41.26-47.75)	
	DHS 2006/07	(5)	798/1335	60.65	(57.26-63.95)	0.3125	1480/3180	44.92	(42.75-47.10)	0.7207
	DHS 2013	(6)	755/1301	57.44	(54.19-60.62)	0.1323	1352/3041	40.92	(38.88-42.98)	0.0051
**Rwanda**	DHS 2000	(4)	202/627	33.88	(29.81-38.20)		845/2738	31.92	(29.78-34.15)	
	DHS 2005	(4)	412/1113	36.70	(33.55-39.95)	0.4745	882/2933	29.70	(27.99-31.47)	0.1697
	DHS 2010	(6)	474/1616	29.82	(27.45-32.31)	0.0186	876/3714	23.70	(22.25-25.22)	<0.0001
	DHS 2014/15	(6)	376/1414	26.39	(23.89-29.04)	0.0811	798/3603	22.05	(20.60-23.57)	0.0664
**Tanzania**	DHS 1996	(3)	364/604	58.63	(54.25-62.88)		1836/2927	62.81	(60.16-65.39)	
	DHS 1999	(3)	530/957	58.58	(53.20-63.76)	0.9093	930/1425	68.38	(64.23-72.26)	0.0248
	AIS 2003/04	(5)	754/1748	43.61	(40.88-46.38)	<0.0001	1467/2531	57.30	(54.88-59.69)	<0.0001
	DHS 2004/05	(4)	344/748	48.26	(44.00-52.55)	0.0942	2140/3467	64.29	(61.99-66.52)	<0.0001
	AIS 2007/08	(5)	713/1655	46.66	(43.61-49.73)	0.5704	1646/2860	60.15	(57.74-62.52)	0.0122
	DHS 2010	(5)	281/621	47.93	(43.37-52.53)	0.6494	1792/3099	61.51	(59.11-63.87)	0.4229
	AIS 2011/12	(6)	928/2247	44.31	(41.58-47.08)	0.1659	1802/3428	53.11	(50.43-55.77)	<0.0001
	DHS 2015/16	(7)	474/995	49.87	(46.17-53.56)	0.0156	2469/4202	60.62	(58.55-62.65)	<0.0001
**Uganda**	DHS 1995	(3)	444/715	60.62	(56.77-64.34)		2040/2803	73.86	(71.63-75.98)	
	DHS 2000/01	(4)	230/602	38.98	(34.72-43.41)	<0.0001	1857/2783	68.58	(66.36-70.73)	0.0010
	DHS 2006	(5)	355/681	51.85	(47.56-56.12)	0.0001	1977/2921	68.87	(66.67-70.99)	0.8223
	DHS 2011	(6)	279/639	43.86	(39.33-48.49)	0.0210	1859/3101	62.74	(60.52-64.91)	0.0001
	DHS 2016	(7)	804/1561	50.90	(47.93-53.86)	0.0316	3973/6474	60.01	(58.27-61.72)	0.0504
**Zambia**	DHS 1996	(3)	465/607	75.30	(71.02-79.14)		2222/3008	71.71	(69.72-73.61)	
	DHS 2001/02	(4)	418/665	61.20	(56.52-65.68)	<0.0001	1959/2895	65.65	(63.45-67.78)	0.0001
	DHS 2007	(5)	1094/1894	55.54	(52.70-58.35)	0.0351	1679/2659	61.96	(59.38-64.47)	0.0400
	DHS 2013/14	(6)	2081/3900	50.96	(48.91-53.01)	0.0062	3275/5498	58.95	(57.13-60.75)	0.0537
**Zimbabwe**	DHS 1994	(3)	263/621	39.90	(35.40-44.57)		947/1945	46.43	(43.71-49.16)	
	DHS 1999	(4)	289/761	35.65	(31.41-40.13)	0.2607	832/2053	37.98	(35.36-40.67)	<0.0001
	DHS 2005/06	(5)	662/2070	30.86	(28.61-33.20)	0.0308	1295/3008	42.18	(40.10-44.28)	0.0147
	DHS 2010/11	(6)	592/2098	26.94	(24.91-29.07)	0.0409	1330/3188	40.25	(38.26-42.27)	0.2621
	DHS 2015	(7)	705/2200	31.31	(28.87-33.85)	0.0132	1353/3107	45.27	(42.89-47.67)	0.0009

Sample sizes (n/N) refer to unadjusted numbers of people reporting an age at first sex before the age of 18 years (n) among everyone who reported sexual intercourse before with data on this variable and who were aged 20-29 years (N). Proportions (%) and 95% confidence intervals (95% CI) are adjusted for survey design and sampling weights. The p-values refer to results from logistic regressions with odds ratios of age at first sex before 18 years calculated for one survey compared with the preceding one, adjusted for age. These are also adjusted for survey design and sampling weights.

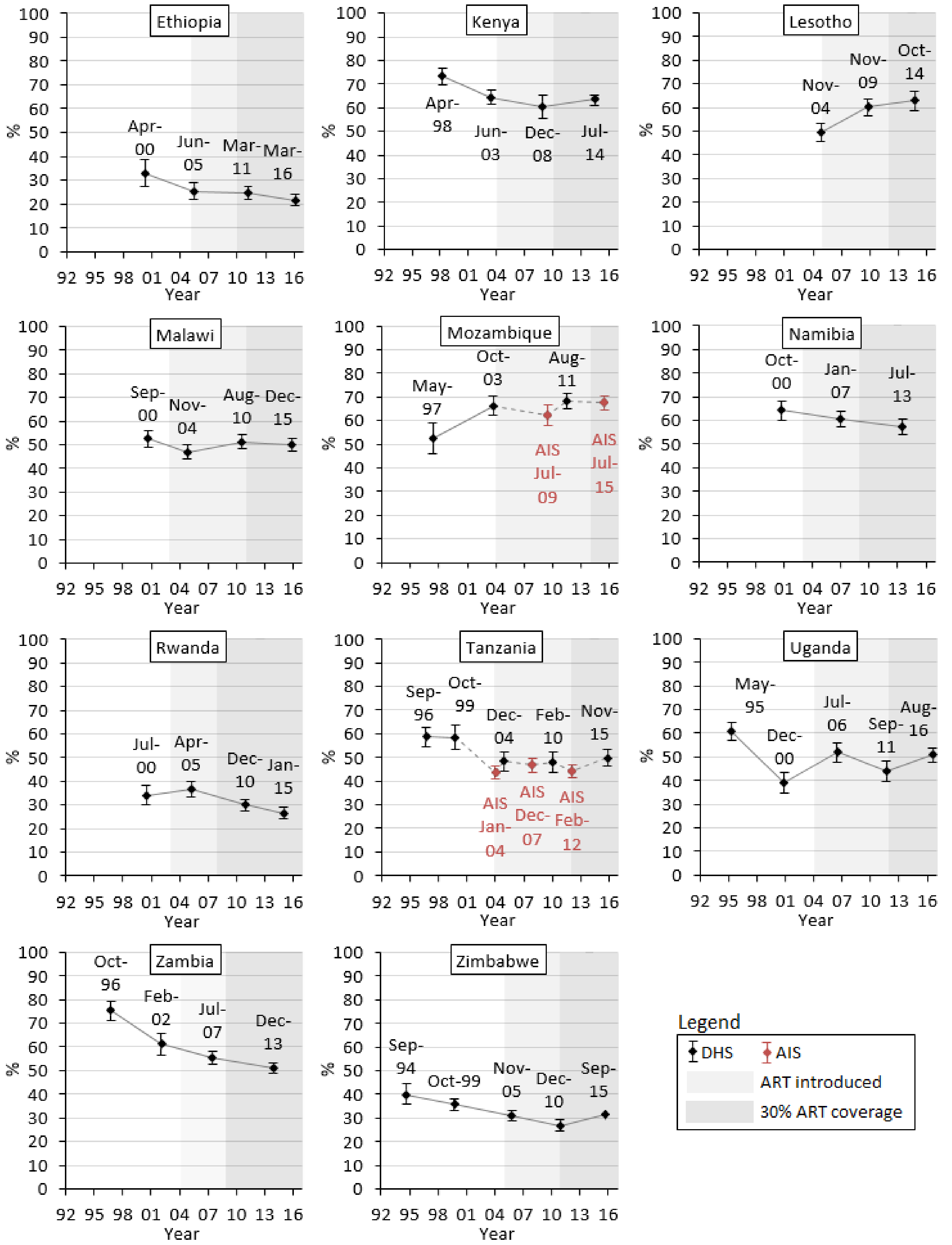

Figure S3a. Trends in age at first sex before the age of 18 years among males (20-29 years), eastern and southern Africa. The sample included males aged 20-29 years who reported ever having had sexual intercourse. Dates refer to the mid-points of the survey data collection period. Data from AIS are indicated in red. Data from different survey types are linked with dashed lines. Shaded areas indicate the years in which ART was introduced into the public healthcare sector in each country and from when 30% of adult PLWH (15+ years) were in treatment (disregarding treatment eligibility criteria; see Supporting Information, Section 2).

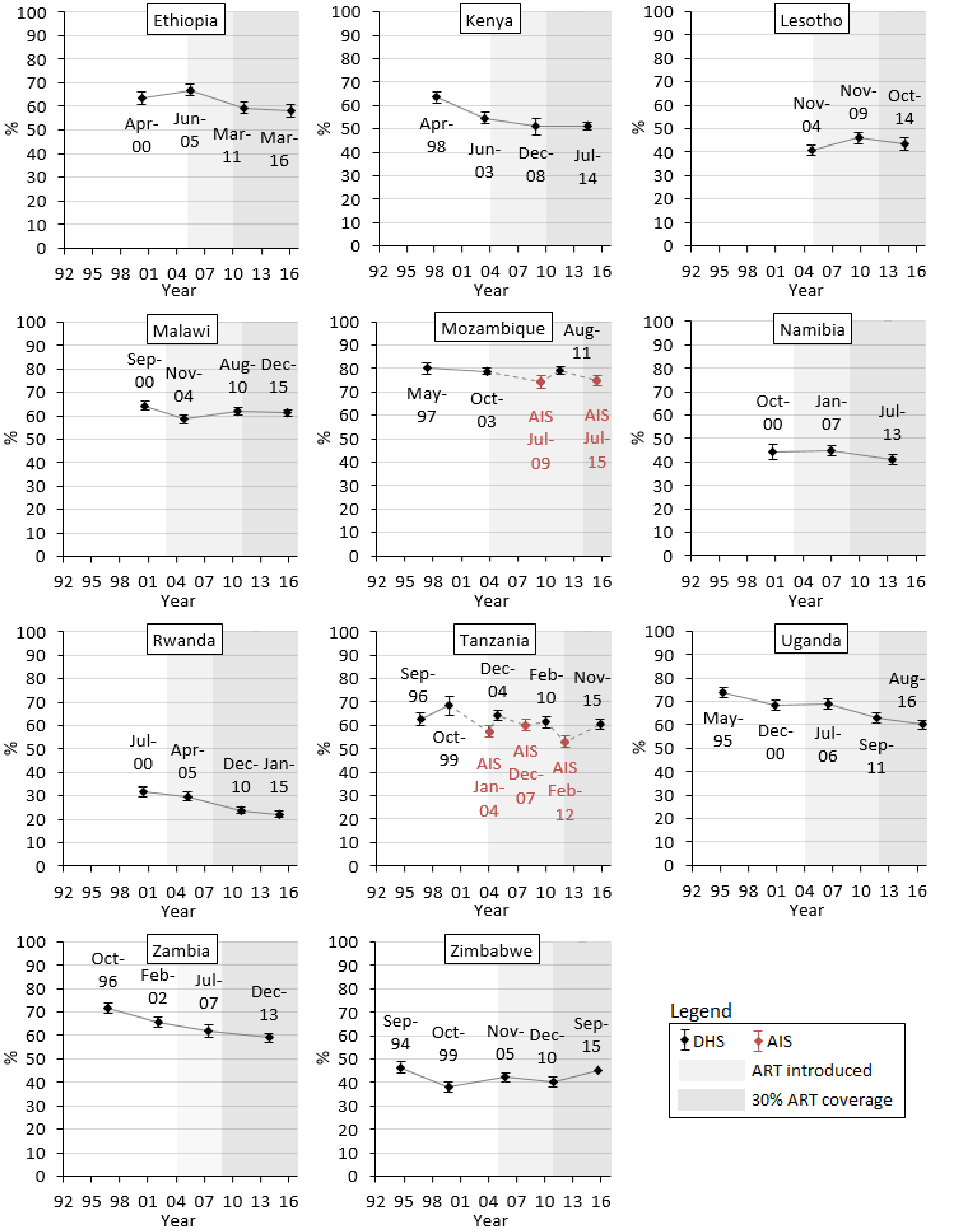

Figure S3b. Trends in age at first sex before the age of 18 years among females (20-29 years), eastern and southern Africa. The sample included females aged 20-29 years who reported ever having had sexual intercourse. Dates refer to the mid-points of the survey data collection period. Data from AIS are indicated in red. Data from different survey types are linked with dashed lines. Shaded areas indicate the years in which ART was introduced into the public healthcare sector in each country and from when 30% of adult PLWH (15+ years) were in treatment (disregarding treatment eligibility criteria; see Supporting Information, Section 2).

5. Analysis by HIV status

Five countries had at least three surveys with HIV status data available (Lesotho, Malawi, Rwanda, Tanzania, and Zimbabwe) (Table S1), which allows for comparing trends in risky sexual behaviour over time between those HIV-negative and HIV-positive. This HIV status was determined at the time of the survey and does not necessarily mean that participants are aware of their status. Particularly in earlier surveys, the proportion of participants who have ever done an HIV test is likely to have been low.

Only multiple sexual partnerships, non-regular partnerships, and condom use during last non-regular partnership are considered here. Figures S4-6 show trends in these behaviours over time by sex and HIV status. To formally test whether there are differences in trends by HIV status, logistic regression models were estimated with the sexual behaviour as the outcome and age, survey, HIV status, and an interaction term between the survey and HIV status as independent variables. Regressions included a survey variable covering all surveys for a country (not just consecutive surveys as in previous regressions). Regressions are estimated separately by sex. Results are presented in Table S10.

As can be seen from the figures and the interaction terms, trends for those HIV-positive and HIV-negative tend to be similar. Levels of multiple partnerships tended to be higher among those HIV-positive (Figure S4a-b). Only In Tanzania there is a markedly different trend between those HIV-positive and HIV-negative, although this interaction is not significant (Table S10). For non-regular partnerships, levels among those HIV-negative tend to be higher among males but lower among females (Figure S5a-b), while trends are generally similar (with the exceptions of Zimbabwe among males and Lesotho among females). For condom use with the last non-regular partner, significant interactions are indicated for Lesotho (males) and Zimbabwe (females) (Table S10). However, the direction of the trend is the same; only the magnitude of increase varies by HIV status.

Table S10: Interaction of sexual risk behaviour and HIV status, eastern and southern Africa.

		**Males**			**Females**		
		**Multiple partners**	**Non-regular partners**	**Condom use non-regular partners**	**Multiple partners**	**Non-regular partners**	**Condom use non-regular partners**
**Country**	**Interaction**	**p-value**	**p-value**	**p-value**	**p-value**	**p-value**	**p-value**
**Lesotho**	DHS 2004#HIV status						
	DHS 2009#HIV status	0.4961	0.5222		0.8643	0.1478	0.4014
	DHS 2014#HIV status	0.7053	0.5545	0.0432	0.4989	0.9264	0.9617
	Overall interaction	0.5135	0.7810	0.0432	0.6780	0.2210	0.6087
**Malawi**	DHS 2004#HIV status						
	DHS 2010#HIV status	0.6654	0.7215	0.9356	0.5970	0.5879	0.4014
	DHS 2015/16#HIV status	0.5037	0.8991	0.1851	0.4241	0.6119	0.9617
	Overall interaction	0.7996	0.8615	0.2344	0.7249	0.8439	0.0885
**Rwanda**	DHS 2005#HIV status						
	DHS 2010#HIV status	0.2293	0.3055	0.3408	0.5044	0.5879	0.4014
	DHS 2014/15#HIV status	0.5893	0.6481	0.1011	0.5970	0.6119	0.9617
	Overall interaction	0.4363	0.5892	0.2606	0.2314	0.7608	0.6970
**Tanzania**	AIS 2003/04#HIV status						
	AIS 2007/08#HIV status	0.0910	0.7654	0.0539	0.9591	0.5879	0.4014
	AIS 2011/12#HIV status	0.5068	0.8558	0.2434	0.3686	0.6119	0.9617
	Overall interaction	0.2399	0.9532	0.1424	0.6123	0.2710	0.2755
**Zimbabwe**	DHS 2005/06#HIV status						
	DHS 2010/11#HIV status	0.2291	0.0198	0.5013	0.5287	0.5879	0.4014
	DHS 2015#HIV status	0.2217	0.0787	0.4024	0.2954	0.6119	0.9617
	Overall interaction	0.3993	0.0581	0.6756	0.5753	0.5688	0.0214

For each country and by sex, a logistic regression was estimated with one sexual behaviour variable as the outcome and age, the survey, HIV status, and an interaction term between survey and HIV status as independent variables. The p-values refer to the individual interactions of the surveys and the interaction as a whole. Samples included everyone who reported sexual activity in the past 12 months. Regressions adjusted for survey design and sampling weights.

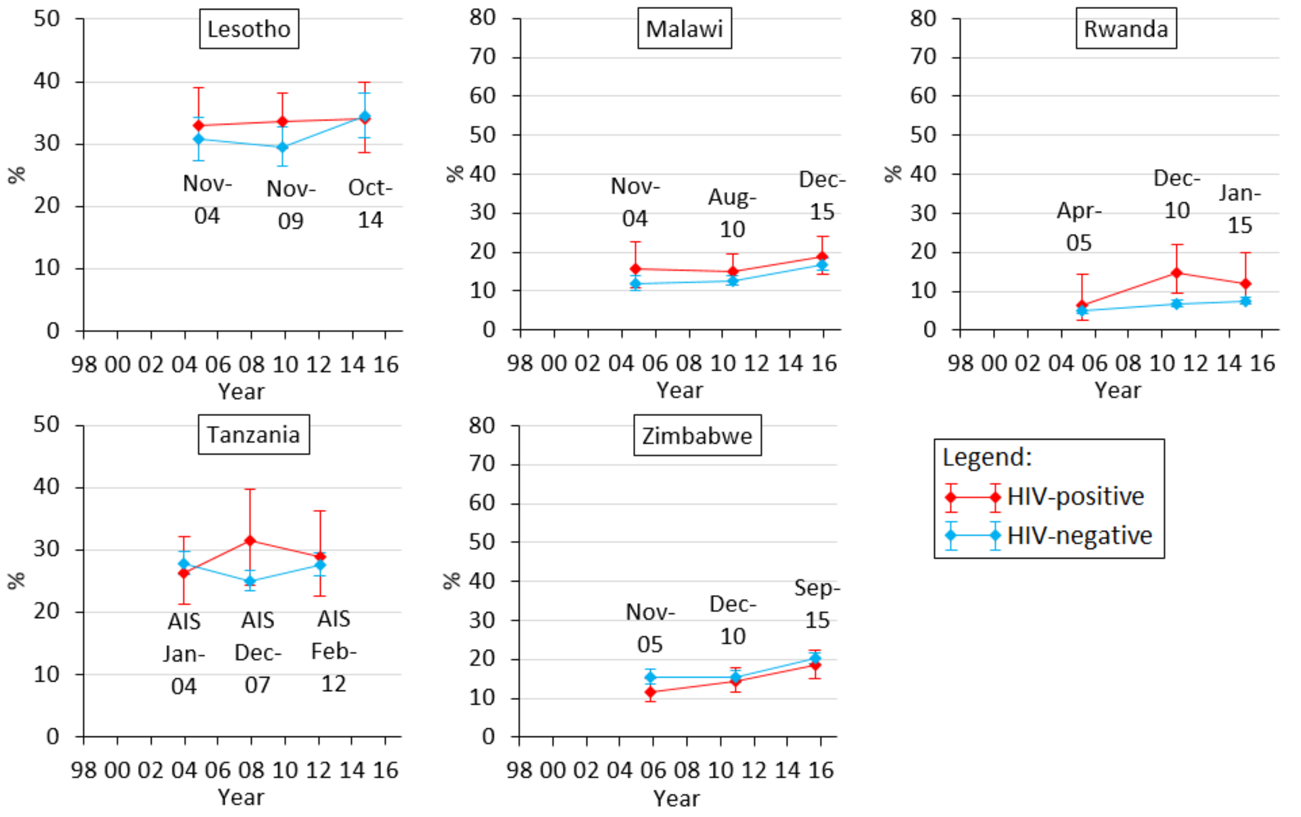

Figure S4a. Trends in multiple sexual partnerships among males (15-49 years) by HIV status, eastern and southern Africa. Multiple sexual partnerships were defined as reporting more than one sexual partner in the past 12 months. Dates refer to the mid-points of the survey data collection period.

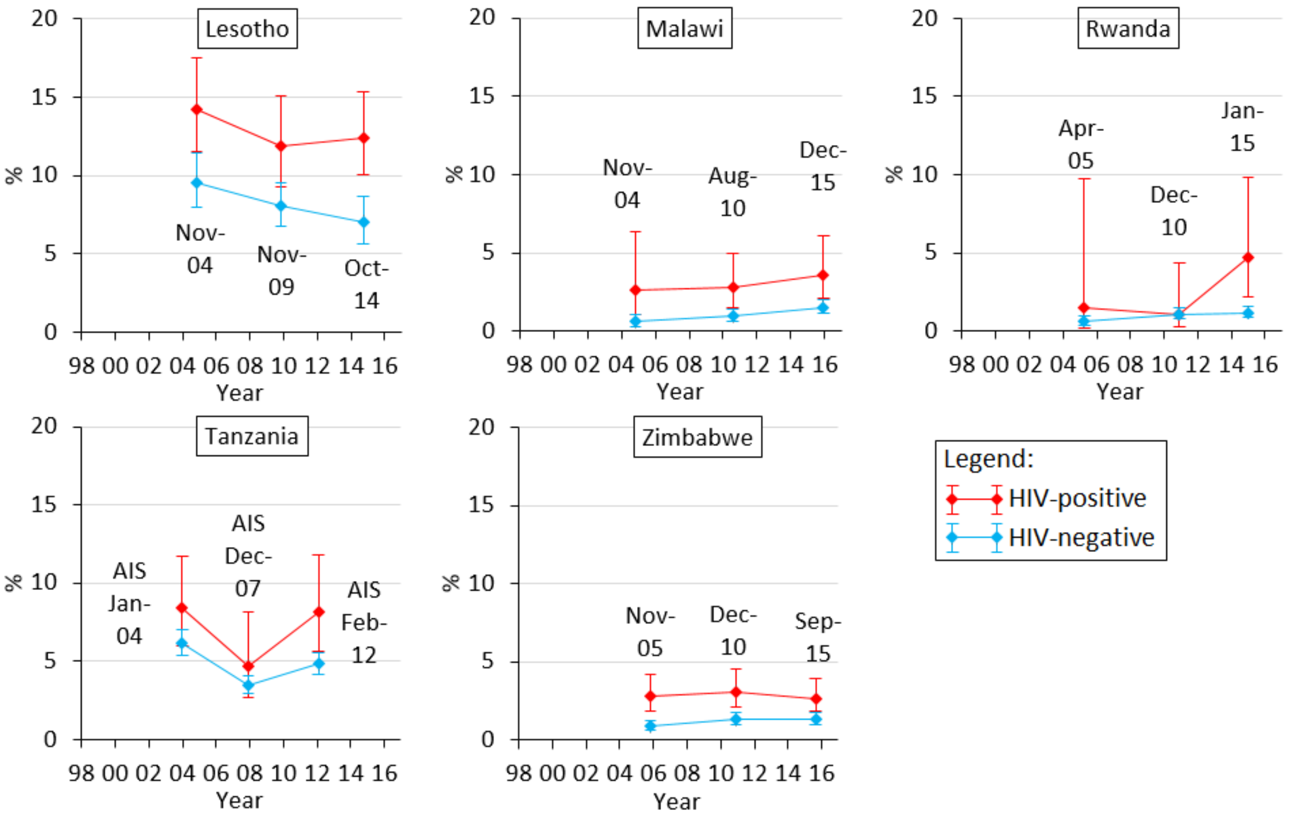

Figure S4b. Trends in multiple sexual partnerships among females (15-49 years) by HIV status, eastern and southern Africa. Multiple sexual partnerships were defined as reporting more than one sexual partner in the past 12 months. Dates refer to the mid-points of the survey data collection period.

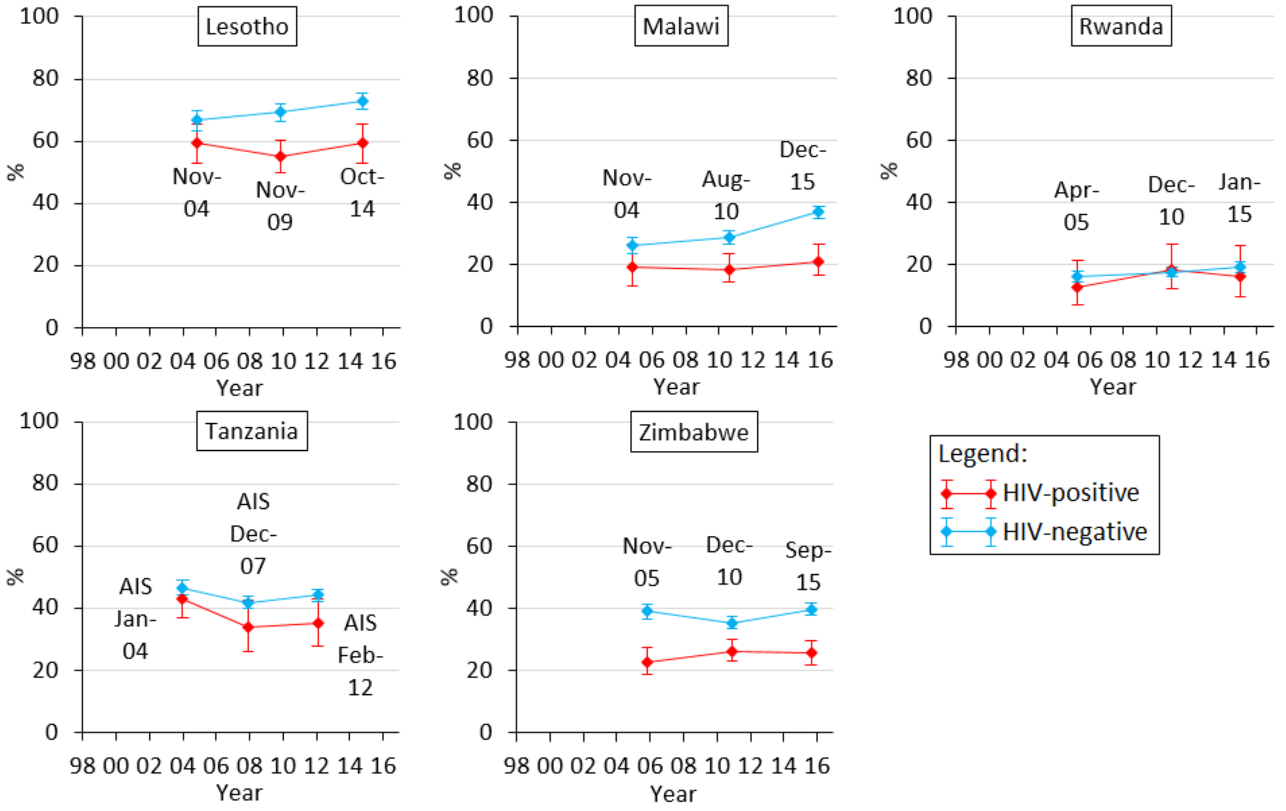

Figure S5a. Trends in non-regular sexual partnerships among males (15-49 years) by HIV status, eastern and southern Africa. Non-regular sexual partnerships were defined as reporting at least one sexual partner in the past 12 months who the participant was not married to and did not live with. Dates refer to the mid-points of the survey data collection period.

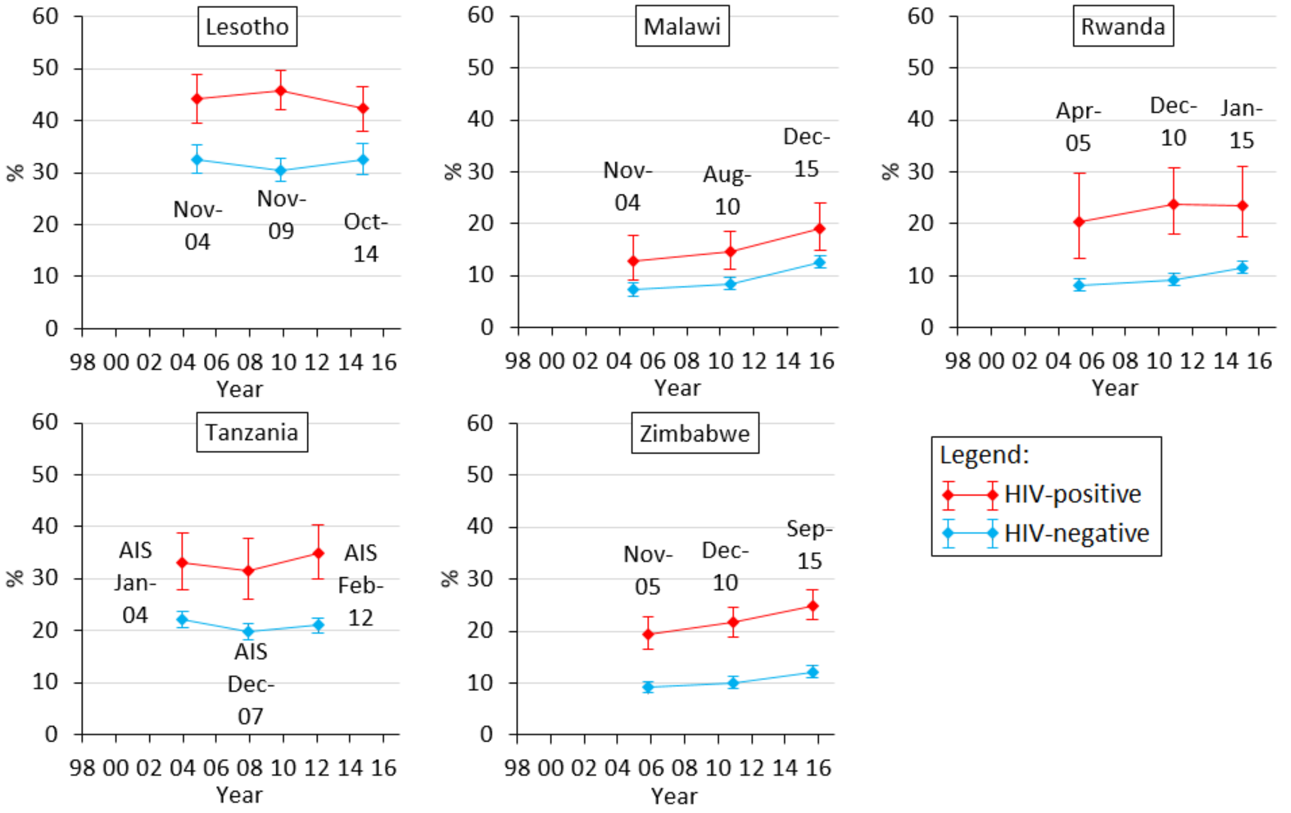

Figure S5b. Trends in non-regular sexual partnerships among females (15-49 years) by HIV status, eastern and southern Africa. Non-regular sexual partnerships were defined as reporting at least one sexual partner in the past 12 months who the participant was not married to and did not live with. Dates refer to the mid-points of the survey data collection period.

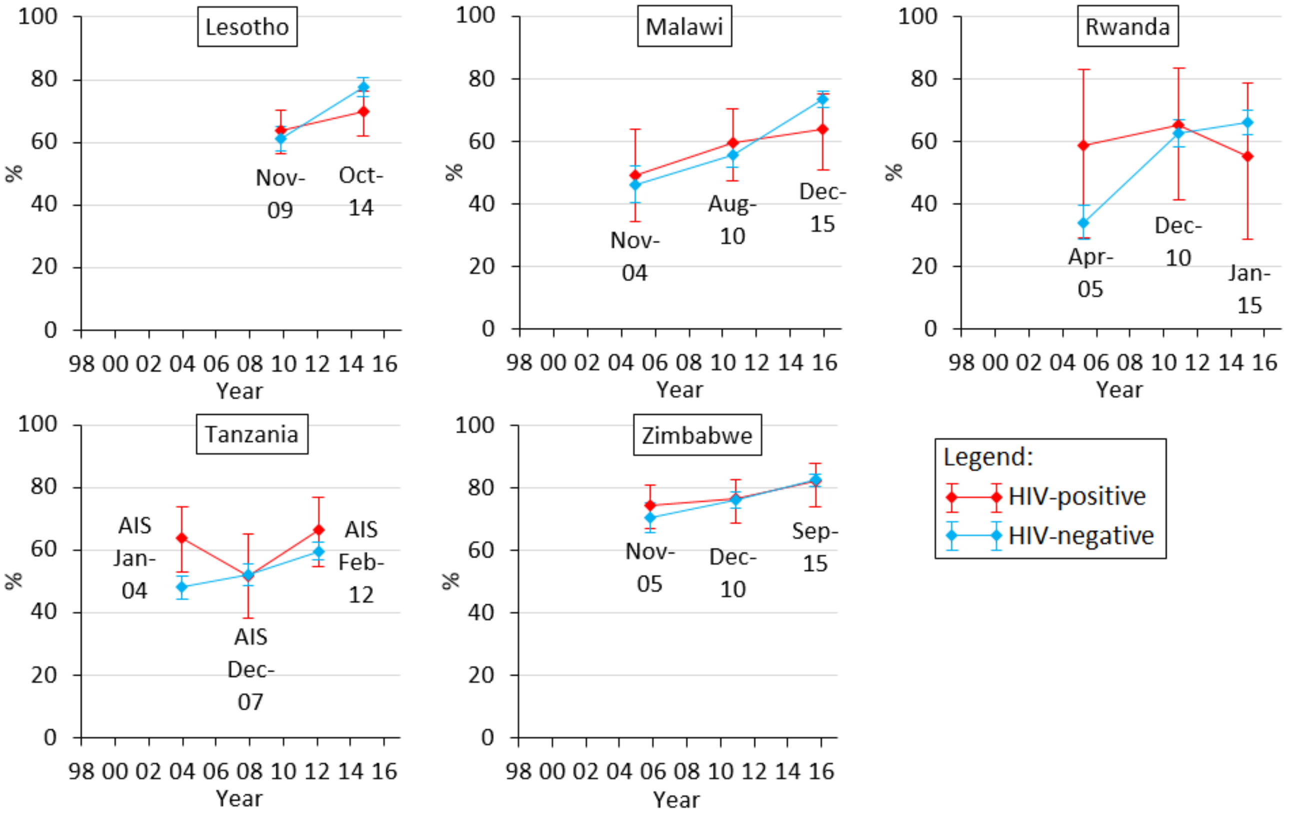

Figure S6a. Trends in condom use with the last non-regular sexual partners among males (15-49 years) by HIV status, eastern and southern Africa. The condom use refers to the last sexual intercourse in the past 12 months with a partner who the participant was not married to and did not live with among everyone who had such a non-regular partner in the past 12 months. Dates refer to the mid-points of the survey data collection period.

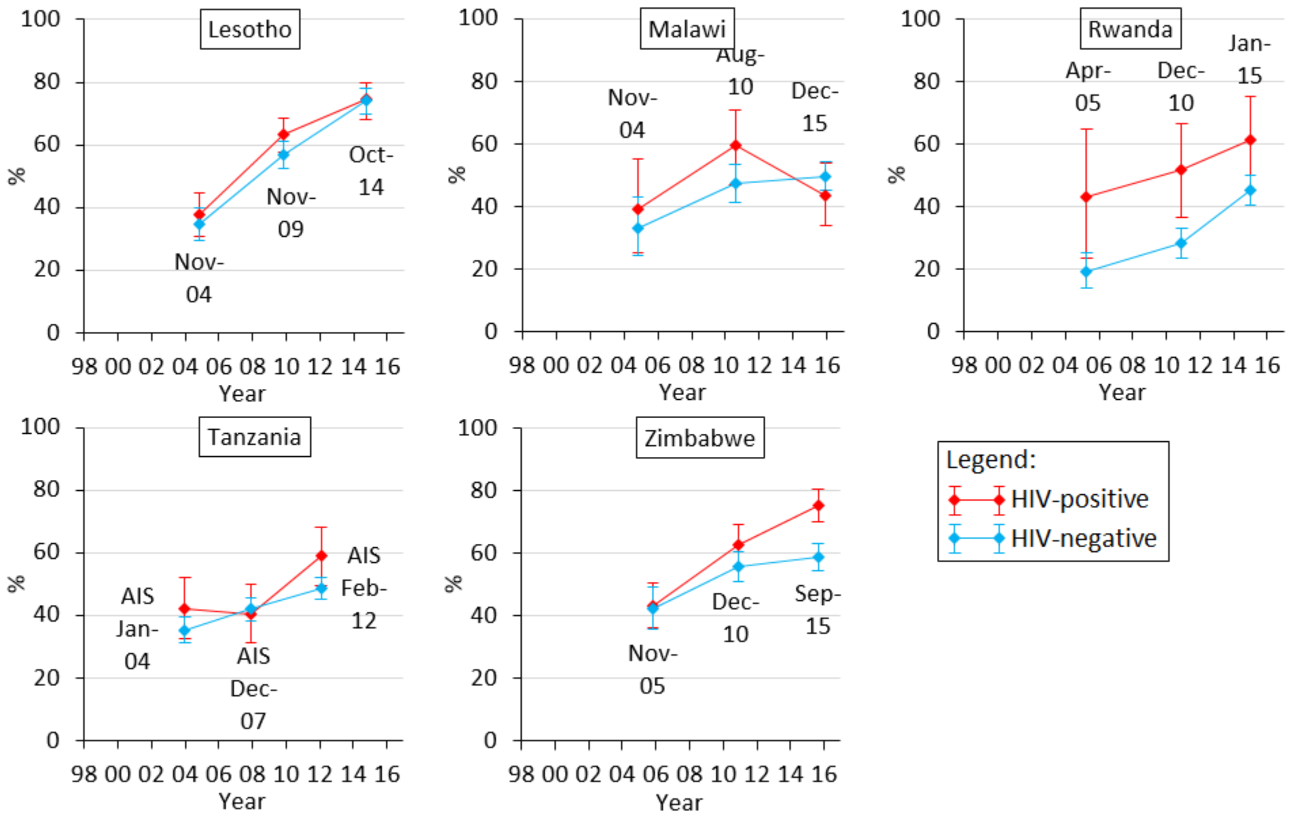

Figure S6b. Trends in condom use with the last non-regular sexual partners among females (15-49 years) by HIV status, eastern and southern Africa. The condom use refers to the last sexual intercourse in the past 12 months with a partner who the participant was not married to and did not live with among everyone who had such a non-regular partner in the past 12 months. Dates refer to the mid-points of the survey data collection period.

6. Sensitivity analysis: Including non-sexually active individuals

The analyses presented in the main article and other analyses in this supplementary material were restricted to those who ever had sex (and further those reporting sexual activity in the past 12 months). Conclusions drawn from these analyses regarding trends in the sexual behaviour may be biased if there are changes in levels of sexual activity in the population. For example, if the proportion of those who ever had sex declines over time then there may be declines in population levels of multiple sexual partnerships in the population, even if there are increases in multiple sexual partnerships among those who are sexually active.

Table S11 and Figure S7a-b presents trends in reporting of having had sex before. The trends in sexual activity reflect, to a large degree, the trends in age of sexual debut presented in Figures S4a-b. Particularly in earlier surveys among males (Figure S7a), there were declines in the proportion of those reporting sexual debut, often correlating with decreasing proportions reporting an age of first sex before the age of 18 (Figure S3a), while trends among females were less pronounced (Figure S7b). Generally, changes in tended to be small in absolute terms. More importantly, decreases in the proportion reporting sexual debut tended not to occur between later surveys (exceptions are Malawi, and Namibia for males). This means it is unlikely that the conclusions drawn from the main analyses of recent increases in risky sexual behaviour are invalid due to overall decreases in sexual activity in recent surveys.

To test formally whether conclusions are impacted by the selected sample, a sensitivity analysis was conducted to estimate the proportions and statistical differences over time for the three primary indicators of the main analysis (multiple, non-regular, and casual partnerships) including those who have not had sex before and who have not had sex in the past 12 months (who were recorded as not reporting multiple, non-regular, or casual partnerships). Moreover, proportions of those aged 20-29 reporting an age at first sex before the age of 18 were estimated in this sample including those who not had their sexual debut as estimates in this sample may be particularly sensitive to changes in the proportion of those sexually active. The results are presented in Table S12 (multiple partnerships), S13 (non-regular partnerships), S14 (casual partnerships), and S15 (age at first sex). Generally, there is limited indication that the conclusions drawn in the main analysis were biased by restricting the sample to those sexually active. The same trends appear in the sample including those not sexually active and similar statistical differences between surveys within countries tended to be found in this sample. There were few instances in which a difference between surveys was statistically significant in the sample that excludes those who have not had sex compared to the sample that included these (and vice versa). We therefore concluded that no bias was introduced when analysing the sample excluding those who have not had sex before, which is better suited to represent trends in sexual behaviour.

Table S11: Having ever had sex, eastern and southern Africa.

			Males				Females			
			Sample	Adj. proportions		Logit	Sample	Adj. proportions		Logit
Country	Survey	(Phase)	n/N	%	(95% CI)	p-value	n/N	%	(95% CI)	p-value
**Ethiopia**	DHS 2000	(4)	1619/2336	67.15	(64.17-69.99)	0.0000	11787/15356	77.17	(75.61-78.66)	0.0000
	DHS 2005	(4)	3625/5468	63.12	(61.29-64.92)	0.0013	10609/14056	76.36	(74.98-77.69)	0.1500
	DHS 2011	(6)	8682/12858	64.14	(62.51-65.75)	0.9121	12541/16498	74.73	(73.39-76.03)	0.1678
	DHS 2016	(7)	7965/11578	66.94	(65.43-68.41)	0.9189	11962/15683	76.73	(75.30-78.10)	0.7805
**Kenya**	DHS 1998	(3)	2775/3198	86.32	(84.84-87.67)	0.0000	6591/7860	84.20	(83.09-85.25)	0.0000
	DHS 2003	(4)	2766/3337	83.97	(82.34-85.48)	0.0238	6778/8176	83.01	(81.87-84.10)	0.0100
	DHS 2008/09	(5)	2718/3256	83.77	(81.84-85.52)	0.0793	7031/8438	83.29	(81.91-84.59)	0.3853
	DHS 2014	(6)	9921/12002	84.77	(83.85-85.64)	0.5363	26420/31038	85.70	(85.06-86.32)	0.1620
**Lesotho**	DHS 2004	(4)	1995/2488	79.72	(77.84-81.47)	0.0000	5879/7082	83.36	(82.21-84.45)	0.0000
	DHS 2009	(5)	2572/2988	86.40	(84.89-87.77)	0.0000	6508/7624	85.27	(84.17-86.29)	0.0064
	DHS 2014	(6)	2284/2626	87.03	(85.22-88.66)	0.9807	5689/6621	86.49	(85.45-87.46)	0.4157
**Malawi**	DHS 2000	(4)	2603/2911	89.48	(87.58-91.12)	0.0000	11839/13211	89.59	(88.83-90.30)	0.0000
	DHS 2004	(4)	2712/3079	87.18	(85.33-88.83)	0.0008	10445/11675	88.86	(88.06-89.61)	0.0015
	DHS 2010	(5)	5785/6794	85.33	(84.11-86.47)	0.4193	19855/22995	86.42	(85.79-87.03)	0.0004
	DHS 2015/16	(7)	6053/7138	85.72	(84.68-86.70)	0.4950	21589/24562	88.28	(87.71-88.83)	0.0001
**Mozambique**	DHS 1997	(3)	1818/2002	92.27	(89.94-94.09)	0.0000	8027/8664	93.06	(91.71-94.21)	0.0000
	DHS 2003	(4)	2259/2496	90.91	(89.40-92.23)	0.3496	11633/12405	94.31	(93.73-94.84)	0.0867
	AIS 2009	(5)	3788/4156	91.52	(90.05-92.79)	0.1278	5340/5631	95.51	(94.84-96.10)	0.2611
	DHS 2011	(6)	3177/3514	90.30	(88.97-91.48)	0.9724	12688/13745	92.25	(91.61-92.84)	0.0001
	AIS 2015	(7)	4248/4731	91.02	(90.02-91.93)	0.8924	6446/6938	93.80	(93.13-94.41)	0.0014
**Namibia**	DHS 2000	(4)	2405/2692	89.31	(87.36-91.00)	0.0000	5824/6682	85.36	(83.93-86.68)	0.0000
	DHS 2006/07	(5)	3347/3889	86.30	(84.74-87.73)	0.0000	8240/9750	83.67	(82.51-84.77)	0.0257
	DHS 2013	(6)	3321/3926	84.70	(83.11-86.16)	0.0269	7937/9121	86.02	(85.03-86.95)	0.0407
**Rwanda**	DHS 2000	(4)	1743/2526	68.97	(66.83-71.02)	0.0000	7229/10416	70.62	(69.53-71.69)	0.0000
	DHS 2005	(4)	3050/4406	69.15	(67.42-70.82)	0.0452	7775/11316	69.03	(67.98-70.05)	0.0000
	DHS 2010	(6)	3949/5692	69.49	(68.06-70.88)	0.5243	9523/13665	70.02	(69.08-70.94)	0.7236
	DHS 2014/15	(6)	4000/5582	71.66	(70.23-73.04)	0.2808	9907/13494	73.77	(72.90-74.63)	0.0000
**Tanzania**	DHS 1996	(3)	1684/2027	82.90	(80.96-84.68)	0.0000	7027/8092	87.04	(85.96-88.06)	0.0000
	DHS 1999	(3)	2633/3200	86.95	(85.05-88.65)	0.0000	3427/4025	87.68	(85.98-89.21)	0.0292
	AIS 2003/04	(5)	4659/5659	82.87	(81.52-84.15)	0.0000	5967/6857	86.88	(85.76-87.92)	0.0409
	DHS 2004/05	(4)	2108/2633	84.29	(82.46-85.96)	0.0395	8641/10326	86.93	(85.91-87.89)	0.8846
	AIS 2007/08	(5)	5140/6972	79.84	(78.48-81.13)	0.0000	7584/9341	86.82	(85.90-87.69)	0.8587
	DHS 2010	(5)	1920/2527	80.88	(78.77-82.82)	0.3837	8347/10137	86.16	(85.25-87.03)	0.1265
	AIS 2011/12	(6)	6532/8329	81.90	(80.64-83.08)	0.6939	9203/10957	86.40	(85.45-87.30)	0.8822
	DHS 2015/16	(7)	2830/3513	83.25	(81.60-84.77)	0.0009	11285/13266	87.58	(86.79-88.34)	0.0055
**Uganda**	DHS 1995	(3)	1660/1902	86.46	(84.23-88.42)	0.0000	6294/7034	90.26	(89.14-91.27)	0.0000
	DHS 2000/01	(4)	1561/1881	83.00	(80.64-85.13)	0.1513	6330/7237	88.28	(87.25-89.25)	0.0006
	DHS 2006	(5)	1948/2383	81.22	(79.29-83.01)	0.4288	7239/8527	85.34	(84.35-86.28)	0.0000
	DHS 2011	(6)	1795/2188	81.83	(79.66-83.81)	0.4084	7350/8660	85.22	(84.21-86.17)	0.5767
	DHS 2016	(7)	4172/5043	82.49	(81.10-83.80)	0.2940	15827/18498	85.42	(84.67-86.15)	0.9528
**Zambia**	DHS 1996	(3)	1527/1717	88.18	(86.17-89.94)	0.0000	7109/8002	88.20	(87.35-89.00)	0.0000
	DHS 2001/02	(4)	1757/1969	89.72	(88.16-91.09)	0.7484	6768/7642	88.13	(87.08-89.11)	0.2435
	DHS 2007	(5)	5095/6001	83.83	(82.41-85.15)	0.0000	6210/7142	86.67	(85.34-87.89)	0.0009
	DHS 2013/14	(6)	11496/13522	84.19	(83.31-85.03)	0.3421	14244/16383	86.61	(85.80-87.38)	0.8468
**Zimbabwe**	DHS 1994	(3)	1565/2024	77.07	(74.59-79.38)	0.0000	4919/6127	79.07	(77.70-80.38)	0.0000
	DHS 1999	(4)	1829/2483	74.45	(72.29-76.49)	0.0020	4746/5904	79.30	(77.67-80.84)	0.3256
	DHS 2005/06	(5)	4978/6830	73.80	(72.34-75.20)	0.1116	7033/8898	79.24	(78.11-80.32)	0.6974
	DHS 2010/11	(6)	5294/7104	74.77	(73.34-76.15)	0.0052	7509/9171	81.88	(80.78-82.94)	0.4918
	DHS 2015	(7)	6177/8018	75.83	(74.58-77.03)	0.0047	8130/9955	81.51	(80.36-82.60)	0.6724

Sample sizes (n/N) refer to unadjusted numbers of people reporting to have had sex (n) among everyone with data on this variable (N). Proportions (%) and 95% confidence intervals (95% CI) are adjusted for survey design and sampling weights. The p-values refer to results from logistic regressions with odds ratios of having had sex calculated for one survey compared with the preceding one, adjusted for age. These are also adjusted for survey design and sampling weights.

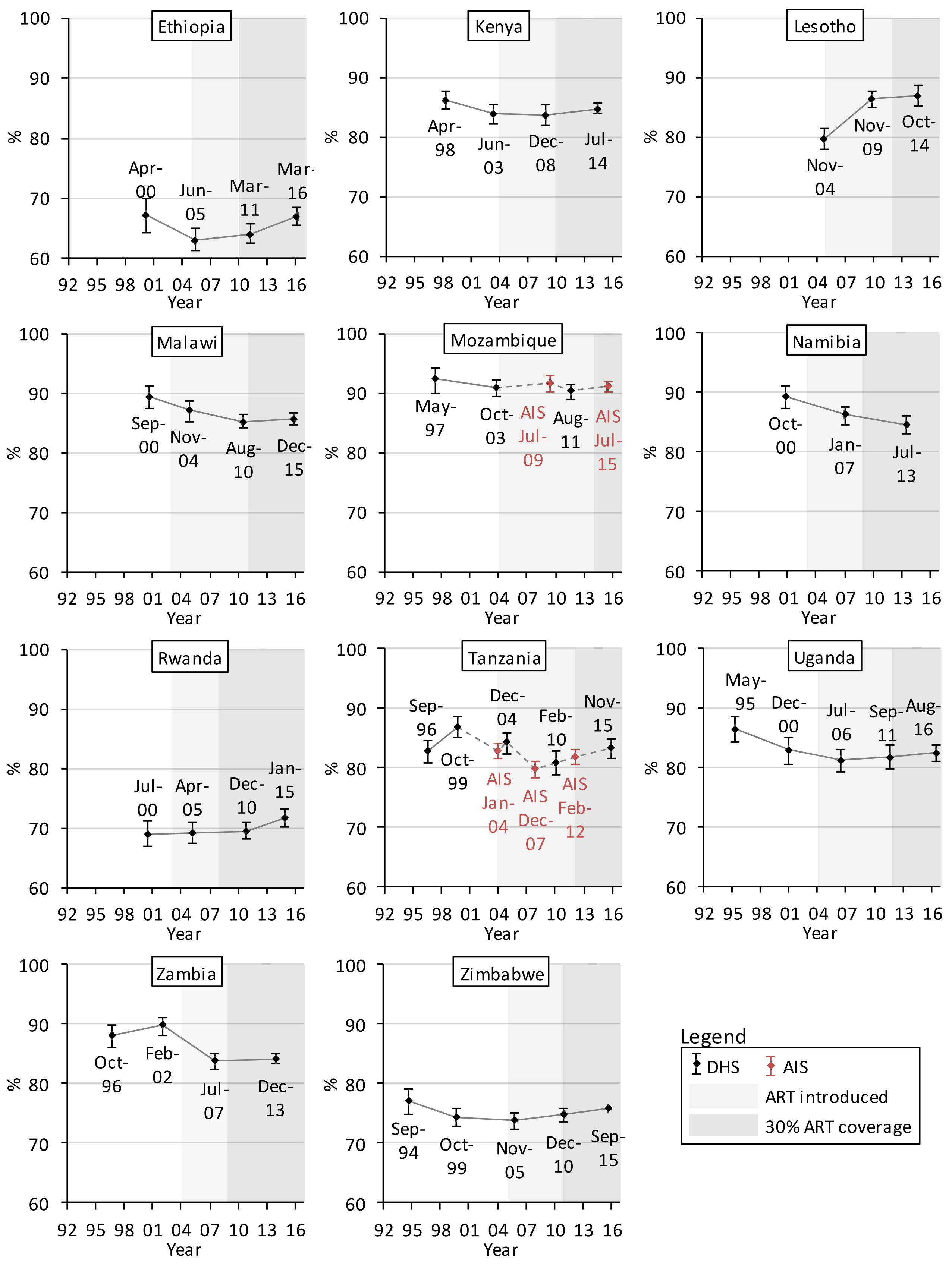

Figure S7a. Trends in reporting of having had sex among males (12-49 years), eastern and southern Africa. The sample included males aged 15-49 years. Dates refer to the mid-points of the survey data collection period. Data from AIS are indicated in red. Data from different survey types are linked with dashed lines. Shaded areas indicate the years in which ART was introduced into the public healthcare sector in each country and from when 30% of adult PLWH (15+ years) were in treatment (disregarding treatment eligibility criteria; see Supporting Information, Section 2).

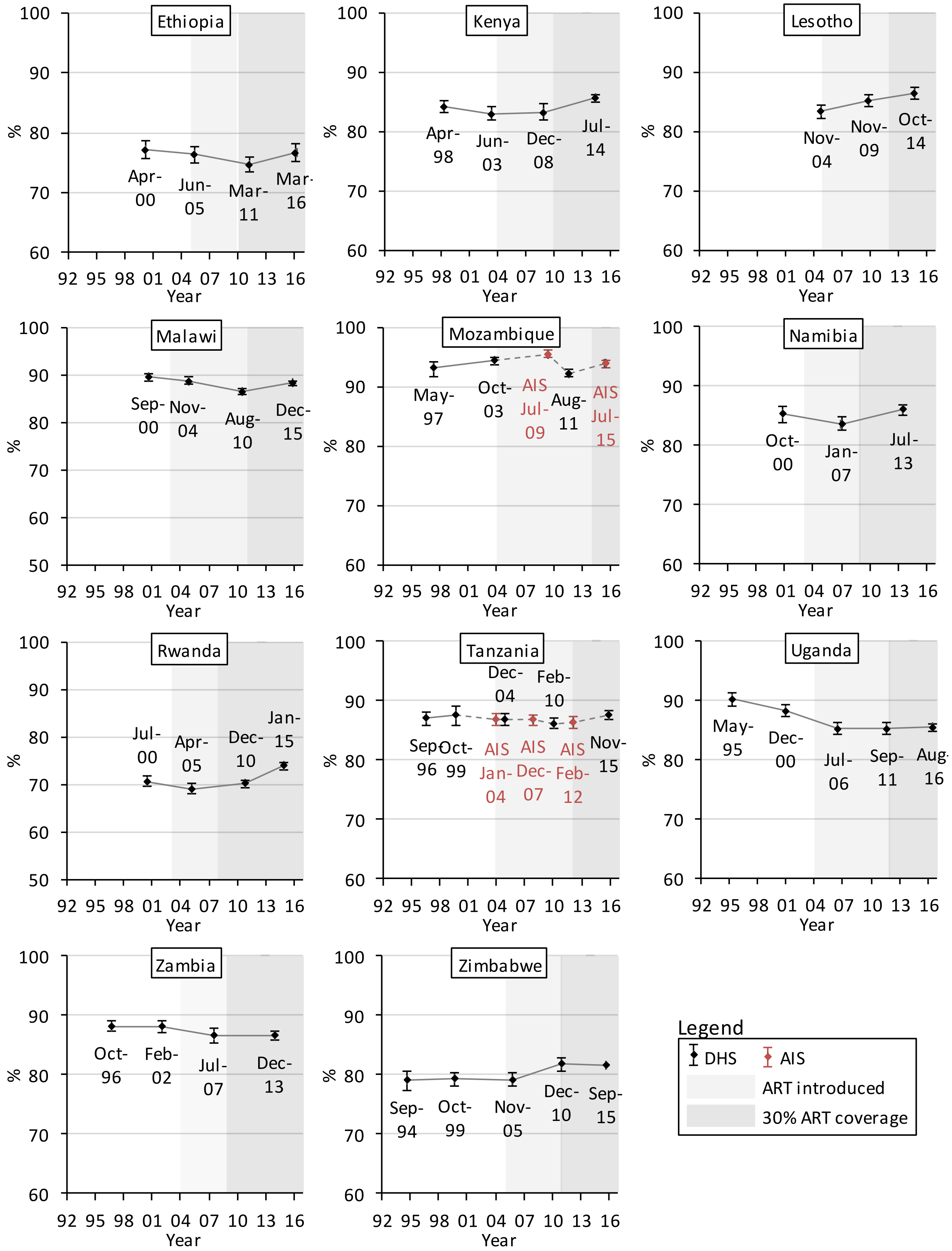

Figure S7b. Trends in reporting of having had sex among females (12-49 years), eastern and southern Africa. The sample included females aged 15-49 years. Dates refer to the mid-points of the survey data collection period. Data from AIS are indicated in red. Data from different survey types are linked with dashed lines. Shaded areas indicate the years in which ART was introduced into the public healthcare sector in each country and from when 30% of adult PLWH (15+ years) were in treatment (disregarding treatment eligibility criteria; see Supporting Information, Section 2).

Table S12: Multiple sexual partnerships (including those who have never had sex before), eastern and southern Africa.

			Males				Females			
			Sample	Adj. proportions		Logit	Sample	Adj. proportions		Logit
Country	Survey	(Phase)	n/N	%	(95% CI)	p-value	n/N	%	(95% CI)	p-value
Ethiopia	DHS 2000	(4)	158/2336	6.54	(5.17-8.25)		141/15352	1.03	(0.75-1.4)	
	DHS 2005	(4)	176/5467	2.36	(1.87-2.98)	<0.0001	15/8579	0.12	(0.06-0.26)	<0.0001
	DHS 2011	(6)	562/12846	3.47	(2.97-4.05)	0.0069	73/16491	0.35	(0.22-0.54)	0.0171
	DHS 2016	(7)	443/11577	3.37	(2.81-4.04)	0.5497	47/15673	0.28	(0.18-0.44)	0.5199
Kenya	DHS 2003	(4)	381/3345	11.71	(10.44-13.12)		130/8170	1.74	(1.43-2.13)	
	DHS 2008/09	(5)	340/3249	9.33	(8.08-10.76)	0.0123	102/8423	1.18	(0.91-1.55)	0.0239
	DHS 2014	(6)	1382/11997	12.70	(11.86-13.59)	0.0002	182/17124	1.21	(0.97-1.51)	0.8990
Lesotho	DHS 2004	(4)	527/2486	21.01	(18.96-23.22)		581/7090	7.71	(6.98-8.51)	
	DHS 2009	(5)	660/2954	22.25	(20.29-24.34)	0.4107	493/7534	6.44	(5.71-7.25)	0.0304
	DHS 2014	(6)	666/2583	26.97	(24.43-29.68)	0.0056	430/6571	6.63	(5.87-7.49)	0.7730
Malawi	DHS 2000	(4)	424/2918	14.56	(13.00-16.28)		121/13220	0.78	(0.62-0.98)	
	DHS 2004	(4)	300/3081	9.13	(7.90-10.52)	<0.0001	97/11696	0.84	(0.67-1.04)	0.6971
	DHS 2010	(5)	651/6790	9.22	(8.40-10.11)	0.8528	132/22986	0.65	(0.53-0.81)	0.1216
	DHS 2015/16	(7)	969/7138	12.87	(11.85-13.97)	<0.0001	285/24558	1.24	(1.07-1.45)	0.0000
Mozambique	DHS 2003	(4)	821/2497	30.24	(27.75-32.85)		603/12405	4.92	(4.32-5.60)	
	AIS 2009	(5)	840/4141	19.62	(17.40-22.05)	<0.0001	180/5645	2.96	(2.39-3.67)	0.0002
	DHS 2011	(6)	1174/3498	29.25	(27.45-31.11)	<0.0001	443/12897	2.93	(2.61-3.28)	0.8027
	AIS 2015	(7)	1028/4722	20.57	(18.72-22.56)	<0.0001	222/6943	2.92	(2.33-3.64)	0.9884
Namibia	DHS 2000	(4)	480/2706	16.39	(13.96-19.15)		156/6701	1.96	(1.60-2.40)	
	DHS 2006/07	(5)	415/3881	11.30	(9.94-12.82)	0.0004	144/9710	1.68	(1.31-2.15)	0.3505
	DHS 2013	(6)	388/3926	10.51	(9.25-11.92)	0.4319	213/9104	2.23	(1.86-2.67)	0.0543
Rwanda	DHS 2000	(4)	66/2531	2.35	(1.76-3.14)		31/10410	0.27	(0.18-0.41)	
	DHS 2005	(4)	119/4410	2.75	(2.23-3.38)	0.4584	37/11319	0.31	(0.22-0.44)	0.6082
	DHS 2010	(6)	227/5694	3.92	(3.45-4.46)	0.0013	79/13667	0.59	(0.47-0.75)	0.0028
	DHS 2014/15	(6)	252/5580	4.51	(3.96-5.13)	0.2372	99/13492	0.70	(0.56-0.87)	0.3078
Tanzania	AIS 2003/04	(5)	1114/5650	20.05	(18.74-21.42)		327/6863	4.71	(4.13-5.36)	
	DHS 2004/05	(4)	542/2629	22.59	(20.40-24.94)	0.0511	296/10326	3.31	(2.86-3.82)	0.0004
	AIS 2007/08	(5)	1061/6958	17.92	(16.74-19.18)	0.0001	196/9329	2.64	(2.25-3.08)	0.0348
	DHS 2010	(5)	472/2525	20.63	(18.54-22.89)	0.0360	291/10139	3.52	(2.99-4.15)	0.0119
	AIS 2011/12	(6)	1585/8334	20.78	(19.39-22.25)	0.9796	365/10943	3.78	(3.31-4.32)	0.5096
Uganda	DHS 2000/01	(4)	363/1882	18.01	(15.93-20.30)		137/7244	1.60	(1.29-1.99)	
	DHS 2006	(5)	498/2383	20.50	(18.58-22.55)	0.1085	141/8516	1.77	(1.46-2.14)	0.5078
	DHS 2011	(6)	412/2190	18.61	(16.62-20.78)	0.1931	142/8658	1.60	(1.29-1.98)	0.4983
	DHS 2016	(7)	1063/5043	20.50	(19.16-21.90)	0.0953	430/18506	2.30	(2.04-2.60)	0.0037
Zambia	DHS 2001/02	(4)	407/1976	21.30	(19.29-23.45)		146/7656	2.07	(1.73-2.48)	
	DHS 2007	(5)	909/5988	14.43	(13.33-15.61)	<0.0001	95/7142	1.20	(0.96-1.50)	0.0003
	DHS 2013/14	(6)	2101/13519	15.65	(14.78-16.57)	0.1525	263/16397	1.68	(1.42-1.99)	0.0184
Zimbabwe	DHS 1999	(4)	344/2487	12.92	(11.46-14.54)		103/5902	1.66	(1.33-2.06)	
	DHS 2005/06	(5)	580/6840	8.99	(8.14-9.92)	<0.0001	77/8902	0.88	(0.67-1.14)	0.0003
	DHS 2010/11	(6)	718/7019	10.66	(9.73-11.66)	0.0214	101/9055	1.11	(0.89-1.38)	0.1704
	DHS 2015	(7)	1163/8014	14.19	(13.28-15.16)	<0.0001	127/9900	1.10	(0.88-1.38)	0.9754

Sample sizes (n/N) refer to unadjusted numbers of people reporting multiple sexual partnerships (n) among everyone (including those who have not had sex before) (N). Proportions (%) and 95% confidence intervals (95% CI) are adjusted for survey design and sampling weights. The p-values refer to results from logistic regressions with odds ratios of multiple sexual partnerships calculated for one survey compared with the preceding one, adjusted for age. These are also adjusted for survey design and sampling weights.

Table S13: Non-regular sexual partnerships (including those who have never had sex before), eastern and southern Africa.

			Males				Females			
			**Sample**	**Adj. proportions**		**Logit**	**Sample**	**Adj. proportions**		**Logit**
**Country**	**Survey**	**(Phase)**	**n/N**	**%**	**(95% CI)**	**p-value**	**n/N**	**%**	**(95% CI)**	**p-value**
**Ethiopia**	DHS 2000	(4)	424/2336	15.83	(13.37-18.65)		613/15344	3.04	(2.56-3.60)	
	DHS 2005	(4)	444/5470	4.90	(4.16-5.76)	0.0000	170/8593	1.45	(1.14-1.83)	<0.0001
	DHS 2011	(6)	1106/12863	5.67	(5.06-6.36)	0.1327	543/16507	2.60	(2.21-3.06)	<0.0001
	DHS 2016	(7)	1129/11578	6.88	(6.02-7.85)	0.0153	483/15683	2.40	(2.01-2.88)	0.5670
**Kenya**	DHS 2003	(4)	895/3343	27.87	(25.88-29.95)		1002/8181	12.29	(11.40-13.23)	
	DHS 2008/09	(5)	821/3256	24.98	(22.99-27.07)	0.1209	1018/8438	12.83	(11.63-14.13)	0.3921
	DHS 2014	(6)	3294/12007	30.21	(28.80-31.66)	<0.0001	1810/17112	12.29	(11.52-13.11)	0.3197
**Lesotho**	DHS 2004	(4)	1128/2492	44.06	(41.66-46.49)		1838/7084	25.41	(24.17-26.70)	
	DHS 2009	(5)	1477/2988	48.84	(46.69-50.99)	0.0034	1861/7624	24.71	(23.44-26.02)	0.4539
	DHS 2014	(6)	1389/2626	53.44	(51.13-55.74)	0.0022	1736/6621	26.50	(25.03-28.03)	0.0739
**Malawi**	DHS 2000	(4)	838/2913	28.04	(25.91-30.27)		1024/13217	7.23	(6.64-7.88)	
	DHS 2004	(4)	632/3081	19.99	(18.21-21.91)	<0.0001	767/11686	6.46	(5.82-7.17)	0.0828
	DHS 2010	(5)	1370/6799	19.92	(18.53-21.39)	0.5262	1472/23007	6.57	(6.08-7.08)	0.6690
	DHS 2015/16	(7)	1968/7138	26.82	(25.39-28.29)	<0.0001	2455/24562	9.93	(9.38-10.51)	<0.0001
**Mozambique**	DHS 2003	(4)	1158/2502	43.57	(40.36-46.84)		2339/12393	17.30	(16.12-18.54)	
	AIS 2009	(5)	1604/4158	34.58	(31.67-37.62)	0.0004	1049/5642	15.38	(13.88-17.02)	0.1383
	DHS 2011	(6)	1770/3514	45.97	(43.74-48.22)	<0.0001	2624/13745	16.36	(15.39-17.38)	0.5965
	AIS 2015	(7)	2007/4733	38.67	(36.42-40.97)	<0.0001	1512/6938	18.13	(16.62-19.74)	0.0495
**Namibia**	DHS 2000	(4)	1266/2700	47.25	(43.70-50.83)		2494/6680	37.26	(34.90-39.68)	
	DHS 2006/07	(5)	1625/3872	42.03	(39.77-44.32)	0.0284	3158/9642	33.20	(31.77-34.67)	0.0043
	DHS 2013	(6)	1837/3922	48.99	(46.71-51.27)	<0.0001	3597/9092	41.09	(39.69-42.50)	<0.0001
**Rwanda**	DHS 2000	(4)	225/2529	7.38	(6.36-8.56)		379/10405	3.35	(2.95-3.81)	
	DHS 2005	(4)	401/4412	8.79	(7.83-9.85)	0.0629	504/11318	4.26	(3.87-4.68)	0.0033
	DHS 2010	(6)	583/5694	9.91	(9.05-10.84)	0.1135	742/13668	5.27	(4.84-5.72)	0.0010
	DHS 2014/15	(6)	656/5581	11.37	(10.38-12.44)	0.0222	1035/13489	7.41	(6.94-7.92)	<0.0001
**Tanzania**	AIS 2003/04	(5)	1899/5655	34.12	(32.41-35.88)		1220/6863	17.81	(16.62-19.07)	
	DHS 2004/05	(4)	775/2634	33.72	(31.28-36.25)	0.7700	1328/10327	14.84	(13.75-16.00)	0.0006
	AIS 2007/08	(5)	1677/6972	29.25	(27.72-30.82)	0.0030	1181/9341	15.92	(14.75-17.16)	0.1611
	DHS 2010	(5)	733/2527	32.89	(30.40-35.49)	0.0133	1501/10137	17.87	(16.82-18.97)	0.0173
	AIS 2011/12	(6)	2476/8342	32.78	(31.27-34.33)	0.9912	1628/10961	16.90	(15.90-17.95)	0.2259
**Uganda**	DHS 2000/01	(4)	438/1881	21.26	(18.95-23.78)		864/7236	10.75	(9.62-11.99)	
	DHS 2006	(5)	606/2384	25.53	(23.54-27.62)	0.0086	980/8527	11.79	(10.93-12.72)	0.1263
	DHS 2011	(6)	533/2190	23.34	(21.00-25.84)	0.1739	967/8664	10.94	(10.18-11.74)	0.1385
	DHS 2016	(7)	1483/5043	30.33	(28.73-31.98)	<0.0001	2670/18502	14.83	(14.07-15.62)	<0.0001
**Zambia**	DHS 2001/02	(4)	701/1973	36.19	(33.69-38.76)		1022/7648	13.17	(12.17-14.22)	
	DHS 2007	(5)	1742/6002	27.65	(26.11-29.26)	<0.0001	998/7142	12.59	(11.63-13.62)	0.7003
	DHS 2013/14	(6)	4016/13517	29.28	(28.17-30.42)	0.0528	2521/16383	14.27	(13.45-15.14)	0.0060
**Zimbabwe**	DHS 1999	(4)	752/2489	28.97	(27.03-31.00)		681/5907	9.75	(8.76-10.85)	
	DHS 2005/06	(5)	1500/6843	22.23	(20.96-23.55)	<0.0001	693/8900	7.56	(6.85-8.33)	0.0006
	DHS 2010/11	(6)	1692/7104	22.73	(21.52-23.99)	0.3087	863/9171	8.09	(7.43-8.81)	0.2610
	DHS 2015	(7)	2203/8017	25.60	(24.32-26.92)	0.0006	1172/9955	10.16	(9.30-11.09)	0.0003

Sample sizes (n/N) refer to unadjusted numbers of people reporting non-regular sexual partnerships (n) among everyone (including those who have not had sex before) (N). Proportions (%) and 95% confidence intervals (95% CI) are adjusted for survey design and sampling weights. The p-values refer to results from logistic regressions with odds ratios of non-regular sexual partnerships calculated for one survey compared with the preceding one, adjusted for age. These are also adjusted for survey design and sampling weights.

Table S14: Casual sexual partnerships (including those who have never had sex before), eastern and southern Africa.

			Males				Females			
			Sample	Adj. proportions		Logit	Sample	Adj. proportions		Logit
**Country**	**Survey**	**(Phase)**	**n/N**	**%**	**(95% CI)**	**p-value**	**n/N**	**%**	**(95% CI)**	**p-value**
**Ethiopia**	DHS 2000	(4)	41/2214	2.55	(1.73-3.75)		91/15270	0.78	(0.55-1.11)	
	DHS 2005	(4)	141/5467	1.51	(1.15-1.98)	0.0267	28/8587	0.16	(0.09-0.29)	<0.0001
	DHS 2011	(6)	345/12855	1.82	(1.52-2.18)	0.2531	83/16491	0.41	(0.27-0.63)	0.0103
	DHS 2016	(7)	356/11574	2.00	(1.60-2.49)	0.4614	55/15677	0.36	(0.24-0.55)	0.6982
**Kenya**	DHS 2003	(4)	233/3206	7.53	(6.38-8.86)		54/8081	0.61	(0.44-0.84)	
	DHS 2008/09	(5)	152/3254	4.68	(3.77-5.80)	0.0007	65/8407	0.90	(0.60-1.36)	0.1465
	DHS 2014	(6)	743/11973	7.28	(6.58-8.05)	0.0002	147/17084	0.87	(0.70-1.09)	0.9964
**Lesotho**	DHS 2004	(4)	72/2447	3.18	(2.41-4.20)		45/7003	0.54	(0.37-0.80)	
	DHS 2009	(5)	256/2988	8.47	(7.39-9.69)	<0.0001	139/7613	2.07	(1.69-2.54)	<0.0001
	DHS 2014	(6)	274/2625	10.73	(9.16-12.54)	0.0334	175/6616	2.62	(2.19-3.13)	0.0942
**Malawi**	DHS 2000	(4)	189/2845	6.99	(5.85-8.32)		47/13192	0.33	(0.23-0.48)	
	DHS 2004	(4)	147/3022	4.96	(4.09-6.01)	0.0118	47/11665	0.34	(0.24-0.50)	0.8995
	DHS 2010	(5)	213/6783	3.45	(2.91-4.08)	0.0029	43/22955	0.21	(0.14-0.33)	0.0961
	DHS 2015/16	(7)	349/7133	4.89	(4.31-5.54)	0.0010	118/24556	0.46	(0.36-0.61)	0.0038
**Mozambique**	DHS 2003	(4)	443/2279	19.98	(17.48-22.73)		1009/12386	8.13	(7.38-8.96)	
	AIS 2009	(5)	477/4110	10.08	(8.71-11.63)	<0.0001	144/5577	2.31	(1.88-2.83)	<0.0001
	DHS 2011	(6)	960/3510	22.47	(20.91-24.10)	<0.0001	605/13697	4.13	(3.73-4.56)	<0.0001
	AIS 2015	(7)	759/4731	14.24	(12.47-16.22)	<0.0001	334/6933	4.22	(3.52-5.05)	0.8277
**Namibia**	DHS 2000	(4)	342/2507	10.71	(8.13-13.99)		60/6639	0.95	(0.65-1.38)	
	DHS 2006/07	(5)	513/3868	13.25	(11.58-15.12)	0.1572	76/9632	0.83	(0.62-1.11)	0.5756
	DHS 2013	(6)	442/3917	10.82	(9.55-12.24)	0.0326	93/9082	0.84	(0.65-1.10)	0.9435
**Rwanda**	DHS 2000	(4)	98/2467	3.34	(2.64-4.22)		71/10262	0.64	(0.49-0.84)	
	DHS 2005	(4)	91/4344	1.97	(1.60-2.43)	0.0012	40/11278	0.33	(0.24-0.46)	0.0019
	DHS 2010	(6)	141/5687	2.37	(2.00-2.81)	0.1849	53/13620	0.41	(0.32-0.54)	0.2903
	DHS 2014/15	(6)	121/5581	2.09	(1.69-2.59)	0.3737	62/13455	0.41	(0.31-0.56)	0.9788
**Tanzania**	AIS 2003/04	(5)	892/5643	15.18	(14.03-16.41)		180/6853	2.57	(2.19-3.02)	
	DHS 2004/05	(4)	413/2633	18.29	(16.50-20.21)	0.0049	150/10311	1.82	(1.50-2.20)	0.0071
	AIS 2007/08	(5)	525/6969	9.61	(8.75-10.56)	<0.0001	117/9334	1.56	(1.27-1.90)	0.2988
	DHS 2010	(5)	173/2521	8.47	(7.15-10.00)	0.1992	81/10119	0.89	(0.68-1.17)	0.0011
	AIS 2011/12	(6)	603/8334	8.02	(7.27-8.84)	0.6129	129/10953	1.17	(0.95-1.45)	0.1123
**Uganda**	DHS 2000/01	(4)	68/1807	3.12	(2.30-4.22)		33/7153	0.42	(0.26-0.67)	
	DHS 2006	(5)	155/2378	6.77	(5.43-8.42)	<0.0001	78/8495	1.09	(0.84-1.40)	0.0004
	DHS 2011	(6)	110/2186	5.06	(3.98-6.41)	0.0764	102/8653	1.31	(1.02-1.67)	0.3018
	DHS 2016	(7)	413/5043	8.35	(7.47-9.32)	0.0002	250/18485	1.40	(1.21-1.63)	0.6349
**Zambia**	DHS 2001/02	(4)	196/1879	11.08	(9.47-12.91)		42/7600	0.56	(0.41-0.76)	
	DHS 2007	(5)	342/5995	5.52	(4.88-6.24)	<0.0001	54/7116	0.67	(0.49-0.93)	0.3595
	DHS 2013/14	(6)	498/13505	3.82	(3.32-4.40)	0.0002	66/16354	0.41	(0.30-0.56)	0.0307
**Zimbabwe**	DHS 1999	(4)	80/2376	4.17	(3.24-5.35)		57/5835	1.08	(0.76-1.52)	
	DHS 2005/06	(5)	200/6832	3.03	(2.57-3.57)	0.0359	28/8886	0.30	(0.20-0.45)	<0.0001
	DHS 2010/11	(6)	241/7100	3.46	(2.98-4.02)	0.2257	45/9157	0.39	(0.28-0.55)	0.3618
	DHS 2015	(7)	391/8015	4.56	(4.02-5.18)	0.0058	67/9951	0.53	(0.36-0.77)	0.2507

Sample sizes (n/N) refer to unadjusted numbers of people reporting casual sexual partnerships (n) among everyone (including those who have not had sex before) (N). Proportions (%) and 95% confidence intervals (95% CI) are adjusted for survey design and sampling weights. The p-values refer to results from logistic regressions with odds ratios of casual sexual partnerships calculated for one survey compared with the preceding one, adjusted for age. These are also adjusted for survey design and sampling weights.

Table S15: Age at first sex before the age of 18 years (including those who have never had sex before), eastern and southern Africa.

			Males				Females			
			Sample	Adj. proportions		Logit	Sample	Adj. proportions		Logit
**Country**	**Survey**	**(Phase)**	**n/N**	**%**	**(95% CI)**	**p-value**	**n/N**	**%**	**(95% CI)**	**p-value**
**Ethiopia**	DHS 2000	(4)	192/786	22.13	(18.17-26.66)		2802/5560	53.10	(50.27-55.91)	
	DHS 2005	(4)	351/1869	14.97	(12.93-17.28)	0.0020	2626/5174	54.87	(52.28-57.44)	0.3191
	DHS 2011	(6)	833/4604	15.36	(13.57-17.33)	0.8978	2946/6207	49.25	(46.83-51.68)	0.0008
	DHS 2016	(7)	656/3999	14.11	(12.40-16.02)	0.2919	2690/5748	47.97	(45.16-50.79)	0.4353
**Kenya**	DHS 1998	(3)	735/1054	68.39	(64.88-71.69)		1651/2886	59.06	(56.65-61.43)	
	DHS 2003	(4)	657/1188	58.48	(55.24-61.64)	<0.0001	1462/3110	49.19	(46.49-51.88)	<0.0001
	DHS 2008/09	(5)	635/1108	55.72	(51.08-60.26)	0.3303	1519/3167	46.46	(42.94-50.02)	0.2299
	DHS 2014	(6)	2233/3923	58.74	(56.54-60.90)	0.2533	5898/11344	47.90	(46.15-49.66)	0.4980
**Lesotho**	DHS 2004	(4)	392/875	44.42	(40.66-48.24)		963/2482	37.67	(35.42-39.96)	<0.0001
	DHS 2009	(5)	619/1093	56.64	(52.90-60.30)	<0.0001	1230/2759	43.66	(41.29-46.06)	0.0003
	DHS 2014	(6)	547/928	59.17	(55.36-62.87)	0.3318	1025/2372	41.57	(38.78-44.41)	0.2629
**Malawi**	DHS 2000	(4)	598/1128	50.23	(46.74-53.72)		3421/5356	62.48	(60.50-64.42)	
	DHS 2004	(4)	550/1200	43.64	(40.51-46.82)	0.0091	2958/4960	56.42	(54.54-58.27)	<0.0001
	DHS 2010	(5)	1057/2281	47.11	(44.28-49.95)	0.1394	5306/8705	59.65	(57.99-61.29)	0.0086
	DHS 2015/16	(7)	1142/2410	46.91	(44.32-49.53)	0.7862	5369/9070	58.87	(57.39-60.34)	0.5204
**Mozambique**	DHS 1997	(3)	366/631	49.78	(43.51-56.06)		2432/3277	78.41	(75.90-80.72)	
	DHS 2003	(4)	532/815	64.82	(60.52-68.89)	0.0001	3556/4659	77.98	(76.34-79.54)	0.7836
	AIS 2009	(5)	852/1388	61.04	(56.84-65.09)	0.2223	1508/2093	73.45	(70.45-76.24)	0.0047
	DHS 2011	(6)	815/1196	67.58	(64.06-70.91)	0.0201	3760/4808	78.59	(76.97-80.13)	0.0015
	AIS 2015	(7)	1066/1633	66.67	(63.48-69.71)	0.6625	1786/2470	74.54	(72.18-76.77)	0.0033
**Namibia**	DHS 2000	(4)	608/1003	61.89	(57.85-65.76)		1044/2426	41.15	(38.14-44.22)	
	DHS 2006/07	(5)	798/1421	57.10	(53.80-60.35)	0.1045	1480/3438	41.22	(39.08-43.41)	0.9161
	DHS 2013	(6)	755/1384	54.24	(51.17-57.28)	0.1894	1352/3215	38.37	(36.41-40.38)	0.0471
**Rwanda**	DHS 2000	(4)	202/824	26.13	(22.84-29.71)		845/3530	25.00	(23.25-26.84)	
	DHS 2005	(4)	412/1598	25.58	(23.25-28.06)	0.6766	882/4101	21.39	(20.05-22.80)	0.0016
	DHS 2010	(6)	474/2205	22.02	(20.22-23.93)	0.0634	876/5187	17.08	(15.96-18.27)	<0.0001
	DHS 2014/15	(6)	376/1963	18.99	(17.12-21.01)	0.0306	798/4792	16.74	(15.56-17.99)	0.6888
**Tanzania**	DHS 1996	(3)	364/668	53.93	(49.75-58.06)		1836/3109	59.57	(56.84-62.24)	
	DHS 1999	(3)	530/1080	55.14	(49.97-60.20)	0.6864	930/1524	65.70	(61.58-69.60)	0.0156
	AIS 2003/04	(5)	754/1922	39.87	(37.24-42.57)	<0.0001	1467/2647	54.78	(52.27-57.25)	<0.0001
	DHS 2004/05	(4)	344/856	44.49	(40.52-48.53)	0.0611	2140/3790	60.98	(58.59-63.32)	0.0004
	AIS 2007/08	(5)	713/2082	41.25	(38.33-44.24)	0.1995	1646/3190	57.53	(55.12-59.91)	0.0443
	DHS 2010	(5)	281/763	43.25	(38.98-47.62)	0.4530	1792/3473	58.10	(55.67-60.50)	0.7421
	AIS 2011/12	(6)	928/2646	39.84	(37.24-42.50)	0.1855	1802/3744	50.23	(47.59-52.87)	<0.0001
	DHS 2015/16	(7)	474/1118	46.18	(42.62-49.78)	0.0049	2469/4577	57.48	(55.37-59.57)	<0.0001
**Uganda**	DHS 1995	(3)	444/760	56.34	(52.23-60.36)		2040/2890	71.79	(69.38-74.08)	
	DHS 2000/01	(4)	230/652	36.08	(31.98-40.39)	<0.0001	1857/2868	66.88	(64.63-69.06)	0.0032
	DHS 2006	(5)	355/748	47.65	(43.49-51.83)	0.0002	1977/3072	65.73	(63.45-67.94)	0.4733
	DHS 2011	(6)	279/705	40.07	(35.86-44.42)	0.0182	1859/3284	59.77	(57.48-62.01)	0.0002
	DHS 2016	(7)	804/1684	47.06	(44.20-49.94)	0.0150	3973/6796	56.99	(55.25-58.72)	0.0680
**Zambia**	DHS 1996	(3)	465/648	69.86	(65.83-73.61)		2222/3103	69.20	(67.06-71.27)	
	DHS 2001/02	(4)	418/699	58.28	(53.66-62.76)	0.0002	1959/3009	62.79	(60.35-65.17)	0.0001
	DHS 2007	(5)	1094/2052	50.78	(47.96-53.59)	0.0064	1679/2779	59.12	(56.46-61.72)	0.0432
	DHS 2013/14	(6)	2081/4240	46.47	(44.45-48.51)	0.0139	3275/5829	55.45	(53.58-57.31)	0.0275
**Zimbabwe**	DHS 1994	(3)	263/675	36.49	(32.43-40.75)		947/2142	41.32	(38.64-44.07)	
	DHS 1999	(4)	289/882	30.58	(26.89-34.54)	0.0423	832/2243	34.21	(31.56-36.95)	0.0003
	DHS 2005/06	(5)	662/2470	25.89	(23.97-27.91)	0.0253	1295/3384	37.54	(35.54-39.58)	0.0538
	DHS 2010/11	(6)	592/2518	22.27	(20.47-24.19)	0.0126	1330/3511	36.40	(34.45-38.39)	0.4443
	DHS 2015	(7)	705/2542	26.80	(24.66-29.06)	0.0021	1353/3438	40.97	(38.61-43.36)	0.0035

Sample sizes (n/N) refer to unadjusted numbers of people reporting an age at first sex before the age of 18 years (n) among everyone (including those who have not had sex before) who was aged 20-29 years (N). Proportions (%) and 95% confidence intervals (95% CI) are adjusted for survey design and sampling weights. The p-values refer to results from logistic regressions with odds ratios of age at first sex before 18 years calculated for one survey compared with the preceding one, adjusted for age. These are also adjusted for survey design and sampling weights.

7. References

1. StataCorp. STATA Survey Data Reference Manual Release 14. College Station, TX: StataCorp LLC; 2015.
2. Ministry of Health Federal Democratic Republic of Ethiopia. National Guidelines for Comprehensive HIV Prevention, Care and Treatment. Addis Ababa: Ministry of Health Federal Democratic Republic of Ethiopia; 2014.
3. UNAIDS. AIDSinfo [Website]. 2018. Available from: http://aidsinfo.unaids.org/ [Accessed 18 April 2018].
4. World Health Organization. Summary Country Profile for HIV/AIDS Treatment Scale-Up: Kenya. Geneva: World Health Organization; 2005.
5. World Health Organization. Summary Country Profile for HIV/AIDS Treatment Scale-Up: Lesotho. Geneva: World Health Organization; 2005.
6. Ministry of Health Lesotho. Global AIDS Response Progress Report 2015: Lesotho Country Report. Maseru: Ministry of Health Lesotho; 2015.
7. World Health Organization. Summary Country Profile for HIV/AIDS Treatment Scale-Up: Malawi. Geneva: World Health Organization; 2005.
8. Council of Ministers. National Strategic HIV and AIDS Response Plan 2010 – 2014 Maputo: National AIDS Council (Conselho Nacional de Combate ao SIDA), Republic of Mozambique; 2010.
9. World Health Organization. Summary Country Profile for HIV/AIDS Treatment Scale-Up: Namibia. Geneva: World Health Organization; 2005.
10. Ministry of Health and Social Services Namibia. United Nations General Assembly Special Session (UNGASS) Country Report: Reporting Period 2008 - 2009. Windhoek: Ministry of Health and Social Services Namibia,; 2010.
11. World Health Organization. Summary Country Profile for HIV/AIDS Treatment Scale-Up: Rwanda. Geneva: World Health Organization; 2005.
12. Rwanda Biomedical Center. National HIV Annual Report 2013-2014. Kigali: Ministry of Health Rwanda; 2014.
13. World Health Organization. Summary Country Profile for HIV/AIDS Treatment Scale-Up: United Republic of Tanzania. Geneva: World Health Organization; 2005.
14. World Health Organization. Summary Country Profile for HIV/AIDS Treatment Scale-Up: Uganda. Geneva: World Health Organization; 2005.
15. World Health Organization. Summary Country Profile for HIV/AIDS Treatment Scale-Up: Zambia. Geneva: World Health Organization; 2005.
16. National HIV/AIDS/STI/TB Council Zambia. National AIDS Strategic Framework 2011-2015. Lusaka: Ministry of Health Zambia; 2010.
17. World Health Organization. Summary Country Profile for HIV/AIDS Treatment Scale-Up: Zimbabwe. Geneva: World Health Organization; 2005.
18. Ministry of Health Zimbabwe. Global AIDS Response Progress Report 2012: Zimbabwe Country Report. Harare: Ministry of Health Zimbabwe; 2012.
